# Sequence Diversity, Locus Structure, and Evolutionary History of the *SpTransformer* Genes in the Sea Urchin Genome

**DOI:** 10.3389/fimmu.2021.744783

**Published:** 2021-11-15

**Authors:** Megan A. Barela Hudgell, L. Courtney Smith

**Affiliations:** Department of Biological Sciences, George Washington University, Washington, DC, United States

**Keywords:** sea urchin, invertebrate immunity, *Strongylocentrotus purpuratus*, gene family evolution, large gene families

## Abstract

The generation of large immune gene families is often driven by evolutionary pressure exerted on host genomes by their pathogens, which has been described as the immunological arms race. The *SpTransformer* (*SpTrf*) gene family from the California purple sea urchin, *Strongylocentrotus purpuratus*, is upregulated upon immune challenge and encodes the SpTrf proteins that interact with pathogens during an immune response. Native SpTrf proteins bind both bacteria and yeast, and augment phagocytosis of a marine *Vibrio*, while a recombinant SpTrf protein (rSpTrf-E1) binds a subset of pathogens and a range of pathogen associated molecular patterns. In the sequenced sea urchin genome, there are four *SpTrf* gene clusters for a total of 17 genes. Here, we report an in-depth analysis of these genes to understand the sequence complexities of this family, its genomic structure, and to derive a putative evolutionary history for the formation of the gene clusters. We report a detailed characterization of gene structure including the intron type and UTRs with conserved transcriptional start sites, the start codon and multiple stop codons, and locations of polyadenylation signals. Phylogenetic and percent mismatch analyses of the genes and the intergenic regions allowed us to predict the last common ancestral *SpTrf* gene and a theoretical evolutionary history of the gene family. The appearance of the gene clusters from the theoretical ancestral gene may have been driven by multiple duplication and deletion events of regions containing *SpTrf* genes. Duplications and ectopic insertion events, indels, and point mutations in the exons likely resulted in the extant genes and family structure. This theoretical evolutionary history is consistent with the involvement of these genes in the arms race in responses to pathogens and suggests that the diversification of these genes and their encoded proteins have been selected for based on the survival benefits of pathogen binding and host protection.

## Introduction

Large, expanded immune gene families in echinoids were first identified in the genome sequence of the purple sea urchin, *Strongylocentrotus purpuratus* (1, 2). They include the Toll-like receptor (*TLR*) gene family that is composed of 253 members (3), the nucleotide oligomerization domain (*NOD*) and the NACHT leucine-rich repeat and PYD containing (*NALP*) gene families (1, 2), the cysteine rich scavenger receptor gene family (1, 4, 5), the *IL-17* cytokine genes (6), and the *SpTransformer* (*SpTrf*) genes of which 15 have been reported previously but whose copy number is likely to vary among individual sea urchins (7, 8). Most of the expanded gene families in the *S. purpuratus* genome encode, or are predicted to encode, proteins with immune function based on i) homologous genes in other species (9), ii) upregulation upon immune challenge (6, 10–13), or iii) patterns of expression and expected markers of gene sequence evolution (see below). The *SpTrf* gene family is upregulated swiftly upon immune challenge in sea urchin immune cells, called coelomocytes (10–13), although expression is restricted to the phagocyte subclass of coelomocytes in adults (13, 14) and the blastocoelar cells in larvae (15). As genes that encode proteins with immune function, native SpTrf proteins opsonize bacteria and augment phagocytosis (16), and one recombinant protein, rSpTrf-E1, binds to Gram negative bacteria, yeast, and several PAMPs (17, 18). The *SpTrf* genes consist of two exons with exon 2 composed 25-27 blocks of sequence called elements, which are present in a mosaic pattern and whose mosaicism makes up the 51 known element patterns (12, 19) that result in a wide range of sequences in exon 2 (12). The *SpTrf* genes also display allelic polymorphism (7) that increases the diversity of the family in individual animals and in the population (20). Allelic polymorphisms impart important diversity in small immune gene families such as those associated with allorecognition including the major histocompatibility complex (MHC) locus in higher vertebrates (21) and the fusion/histocompatibility (Fu/HC) locus in tunicates [reviewed in (22)]. Allelic polymorphism is also observed in large gene families such as the disease resistance (*R*) genes in plants (23). Differences between alleles at specific loci contribute to variation in the immune genes that improves fitness of the host to block and/or survive pathogen infection.

Large gene families can be generated through several processes of genome diversification [reviewed in (24)] that include duplications of large genomic regions, single or tandem duplications that can include coding regions, duplications that result in ectopic insertions as have been reported for *R* genes in plants [reviewed in (25–27)], inversions, meiotic mispairing of clustered genes with similar sequence, unequal crossing over of both intergenic and intragenic regions, and gene conversion in which a sequence from one gene is copied into a nonallelic gene of similar sequence (28). These processes that produce large gene families are the outcome of, and are promoted by, genomic instability (9). These traits are observable in genes under pathogen pressure based on the hypothesis that they are beneficial for maintaining the diversity in immune gene families to optimize fitness in response to pressure from pathogen interactions. In keeping with genomic instability, each *SpTrf* gene is flanked by GA short tandem repeats (STRs) that often includes GAT STRs plus long streatches of GA STR islands that flank two of the clusters (7). Furthermore, there are six different types of imperfect repeats in exon 2, which make up the mosaic pattern within the coding region of these genes and was the basis of the repeat-based alignement (see below) (29). While repeats are common in the sea urchin genome, the placement of the STRs around the genes in this gene family is unique and have been proposed to promote *SpTrf* gene duplication or deletion (7, 30). STRs are known to be highly unstable based on mutation rates that are up to 10 times greater than non-STR genomic DNA, which leads to genomic instability (31–34) and is likely a factor of strand-slipage, unequal crossing over, and/or conversion (35, 36).

The process of maintaining duplicated and altered immune genes is thought to be a response to pathogen pressure followed by selection for improved host fitness. However, the pathogen also responds with counter measures selected to avoid or defeat these new or modified host immune genes and that provide the benefits of successful infection and survival [reviewed in (37, 38)]. Both the host and the pathogen exert fitness pressure in a co-evolutionary arms race, which is known as the Red Queen hypothesis (39). Like the race between Alice and the Red Queen in Luis Carroll’s Through the Looking Glass (40) where the two run a long and hard race only to stay in the same place, infers that a host can survive pathogen pressure only by rapidly changing genes that influence susceptibility or resistance to pathogen infection [(41), reviewed in (42)]. The pressure imposed on the host and the pathogen often leads to genomic regions with large expansive gene families (9, 20, 23, 24). Characteristics of genes involved in an arms race typically show signatures of positive selection, gene multiplicity, elevated recombination rates, and sequence variation that appear as elevated polymorphism and increased species level diversity (37, 38, 43–45). These processes can lead to the generation of complex and highly variable gene families that have the potential benefit of a greater range of pathogen recognition [e.g., (20)]. Some of the more common examples are the human killer cell immunoglobulin-like receptor (*KIR*) genes (46), fibrinogen-related protein genes (*FREP*s) in snails (47), variable region-containing chitin-binding protein (*VCBP*) genes in marine protochordates [(48), reviewed in (49)], *R* genes in higher plants [reviewed in (23)], and NOD-like receptor genes (*NLR*s) in animals (50). This phenomenon of multigene families is also common in other types of receptors, most notably the G-protein coupled receptors (GPCRs), which are mounted on the surface of cells and detect diverse types of external stimuli. These include chemoreceptors (51) such as olfactory receptors that are the largest multigene family in vertebrates (52, 53), some of the taste receptors (54, 55), and other GPCRs that identify large numbers of environmental molecules and trigger signaling pathways (51–55).

Here we present an in-depth bioinformatic and phylogenetic analysis of the sequence diversity of the *SpTrf* gene family that is encoded in the *S. purpuratus* genome sequence. We report an additional cluster of the *SpTrf* genes and describe details of both the coding and flanking regions of the genes. The results enable a proposed theoretical evolutionary history for these genes originating from a last common ancestral (LCA) *SpTrf* gene, which subsequently underwent a number of tandem duplications, ectopic insertions, inversions, and intergenic indels and point mutations to generate the extant clustered genes in the genome sequence. While the genes identified in the sea urchin genome sequence are limited to a single animal, the analysis of these genes can be used as a basis for further work to understand genomic instability in the *SpTrf* gene loci in other *S. purpuratus* individuals that have different genotypes and different numbers of the *SpTrf* genes. These initial results suggest that genomic instability may be a key mechanism to promote diversification of immune gene families in echinoids that are locked in arms races with their pathogens.

## Materials and Methods

### Bacterial Artificial Chromosome Clones

The sea urchin BAC library that was used to generate the genome assembly was the source of the BACs used in this analysis (56). They included BAC 10B1 (GenBank accession number KU668451; 157472 nt), BAC 10K9 (GenBank accession number KU668453; 144627 nt), BAC 10M18 (GenBank accession number KU668450; 74402 nt), and BAC 3104P4 (GenBank accession number KU668454; 118584 nt) (7). The identification of *SpTrf* genes in BAC insert sequences, plus the characterization of element patterns, untranslated regions, introns, and open reading frames were carried out according to Oren et al. (7). GenePalette^1^, a universal software tool for genome sequence visualization and analysis (57), was used to identify the locations of individual *SpTrf* genes within each BAC insert sequence based on the locations of the *SpTrf* primer sequences (R1, F2, F5, F6, R9; see **Supplementary Table S1**). The 5ʹ and 3ʹ ends of the genes were identified using conserved primer sequences [5ʹUTR and 3ʹUTR; **Supplementary Table S1**; and see (7)]. *SpTrf* genes identified in the BAC insert sequences were added to a pre-aligned set of 121 unique *SpTrf* genes and 689 cDNAs with known and identified element patterns as previously reported (11, 19). The deduced amino acid sequences were aligned by hand in BioEdit (ver 7.2.5) (58) to identify the exons and to produce a repeat-based alignment and a cDNA-based alignment as previously reported (12, 19). The exons and the elements were identified and labeled for each *SpTrf* gene and verified based on previously reported genes. Introns were identified for each gene using the repeat-based alignment in BioEdit in which the 3′ end of exon 1 was used to identify the conserved GT splice signal that was approximately 54 nucleotides (nt) from the start codon and the conserved AG splice signal that was located approximately 550 nt from the start codon [see (59)]. Introns were removed from genes to determine whether all genes had open reading frames using NBCI Open Reading Frame Finder^2^.

The cDNA sequence of *Sp0273* [GenBank accession number CK828488.1 (10)] was used to identify the 5′UTR and the TATA box, and the *Sp0065* cDNA sequence [GenBank accession number CK828780 (10)] was used to identify the poly adenylation sites. GenePalette was used to identify additional polyadenylation sites in the 3ʹUTR region of the genes. The 5ʹUTR and 3ʹUTR sequences were identified in GenePalette and verified from partial cDNA sequence data (10).

### PRANK Analysis

Computational alignments of the deduced SpTrf proteins were done using GUIDANCE2^3^ (60–62). Codons were aligned using the multiple sequence alignment (MSA) algorithm in PRANK^4^ (63), an alignment-based software program that processes and identifies the placements of indels. The program was set to trust insertions (F+). Bootstrap guide-trees of 100 iterations were generated, which were further used to calculate 400 alternative alignments using PRANK with F+ before the GUIDANCE2 score was calculated to display whether the alignment was optimal. GUIDANCE scores were analyzed, however because the majority of sequences, columns, and amino acids with low GUIDANCE scores (here defined as >0.8) were associated with the outgroup sequences, the alignments were left unmodified prior to further analysis (data not shown). The deduced translated sequences for the 5ʹ and 3ʹ flanking regions (FRs), introns, and intergenic regions (IGRs) were also aligned with GUIDANCE2 using PRANK with the same parameters. The edges of the FRs included the 5ʹ and 3ʹ UTRs and extended to the location of the flanking GA STRs.

### Sequence Similarity and Percent Mismatch Analysis of *SpTrf* Genes

Sequence similarity among genes with the same or relatively similar element patterns was evaluated with three approaches. i) Percent coverage and percent identity values were established using the basic local alignment search tool (BLAST^5^). ii) Sequence identity matrices were calculated in BioEdit (ver 7.0.5.3) based on the alignment of the deduced proteins. The number of identical residues were calculated while treating gaps as a fifth state to evaluate the similarities among the deduced proteins. iii) A pairwise distance matrix was calculated with Molecular Evolutionary Genetics Analysis [MEGA7^6^, ver 7.0 for larger datasets (64)] using the codon alignment generated in PRANK with preset parameters. All three analyses were run on six regions of the genes that included the 5ʹFR, exon 1, the intron, exon 2, and the 3ʹFR, in addition to the IGRs. Percent mismatches were calculated according to the equation [pairwise distance/Ln^2^], in which the results were generated from the average pairwise distance matrix data for each gene compared to every other gene, divided by Ln^2^, in which the superscript 2 indicates the number of sequences that were compared. A graphical representation of these values was generated using Excel (Microsoft).

### Phylogenetic Trees

MEGA7 was used to generate phylogenetic trees from the PRANK alignments of the 5ʹFR, exon 2, the intron, and the 3ʹFR (~400 nt for the FRs) to determine the evolutionary relatedness among the sequences. Representative *Trf* sequences were selected from the sea urchin, *Heliocidaris erythrogramma* [*HeTrf;* GenBank accession numbers JQ780171-JQ780321; 29 genes; 39 introns (65)], which were used as the outgroup for phylogenetic analyses of both exon 2 and the intron of the *SpTrf* genes. Additional *SpTrf* genes (121 genes, 22 introns) were acquired from Buckley and Smith (19) and used to generate intron and expanded exon 2 trees (**Supplementary Figures S1, S2**). A single *Trf* gene identified from the *Lytechinus variegatus* genome sequence^7^ [*LvTrf; Lv=185/333B3d;* NCBI Accession GCA_000239495.1; Scaffold 232, 80220 to 85000 (66)] was acquired and included 2500 nt on each side of the gene. The 5ʹFR and 3ʹFR (~400 nt) of the *LvTrf* gene were used as the outgroup in the FR alignment of the *SpTrf* genes. Maximum likelihood, neighbor joining, and maximum parsimony with pre-set parameters were used to generate phylogenetic trees. Bootstrap iterations were set to 500 and nodes with a bootstrap value of <50 were collapsed. All trees resulted in similar structure (**Supplementary Figures S3, S4**).

### Dot Plots

Dot plots were generated using YASS^8^ genomic similarity search tool to identify repeats and regions of similarity among genes within and among the four gene clusters. The e-value threshold ranged from 10,000 to e^-30^ as was optimal for different analyses (67). Dot plots from YASS were used to evaluate the sequence variations between allelic BAC 10B1 and BAC 10K9.

### Analysis of Intergenic Regions Among Non-Duplicated Genes

Dot plots were generated using the e-value threshold set to e^-20^. The IGRs between different genes were compared, which consisted of 3 kb flanking the 5ʹ and 3ʹ ends of the allelic *A2* and *A2*a (*A2*/a) genes, the entire 6.9 kb IGRs between the *E2*/a and the *E2*b/*01* genes, 3 kb to the 3ʹ side of the *E2*b/*01* genes, IGRs between the *D1*b/e and *E2*/a genes, and the IGRs between the *D1*h/f genes and the GAT STRs.

### Verification of Allelic BACs

Sequence variations between allelic BAC 10M18 and BAC 3104P4 were analyzed using GenePalette in which GA and GAT STRs were mapped using the sequences GAGAGA and GATGATGAT, respectively, while allowing for a single mismatch. Primers GA1F-GA3F and GA1R-GA3R (**Supplementary Table S1**) were designed to amplify large regions of STRs to evaluate variations in STR lengths using PrimeSTAR GLX high fidelity DNA Polymerase (Takara) to ensure as little polymerase slippage as possible. The PrimeSTAR GLX protocol was 1X PrimeSTAR GXL buffer, 200 μM of each dNTP, 10-15 pmol of each primer, 10 ng BAC DNA, 0.5 U of PrimeSTAR polymerase in a final volume of 20 μL. The PCR program was 95°C for 5 min, followed by 25 cycles of 95°C for 30 sec, 60°C for 30 sec, and 72°C for 4 min with a final extension of 72°C for 7 min and a hold at 4°C. Amplicons were separated on a 0.75% agarose gel with Tris-acetate-EDTA buffer (TAE: 40 mM Tris-acetate pH 8.0, 1.0 mM EDTA).

### Synonymous *vs*. Nonsynonymous Nucleotide Changes

Exon 2 of *SpTrf* genes with the same element pattern were compared to identify synonymous *vs*. nonsynonymous single nucleotide polymorphisms (SNPs). SNPs were catalogued by eye from the alignments and verified with Synonymous Non-synonymous Analysis Program (SNAP^9^; ver 2.1.1) (68) and Single-Likelihood Ancestor Counting (SLAC) (69) in datamonkey^10^ (70–72). The dN/dS value for each gene was calculated based on the Jukes-Cantor corrections (73–75). SNAP was used as an alternative method to evaluate the dN/dS and the number of synonymous *vs*. nonsynonymous substitutions because SNAP was capable of calculating dN/dS values between two genes rather than a group of genes required by SLAC. SLAC was used to confirm results for the seven *D1* genes. Purifying selection was defined as dN/dS of < 1, whereas diversifying selection was defined as > 1. Because the *D1*f and *D1*h gene sequences were identical, they were combined and noted as *D1*f/h for comparisons to the other *D1* genes.

## Results

### Pairs of BAC Inserts Are Likely Allelic Rather Than Clones That Cover Identical Genomic Regions

#### Clusters 3 and 4 Are Allelic

**Figure 1:**
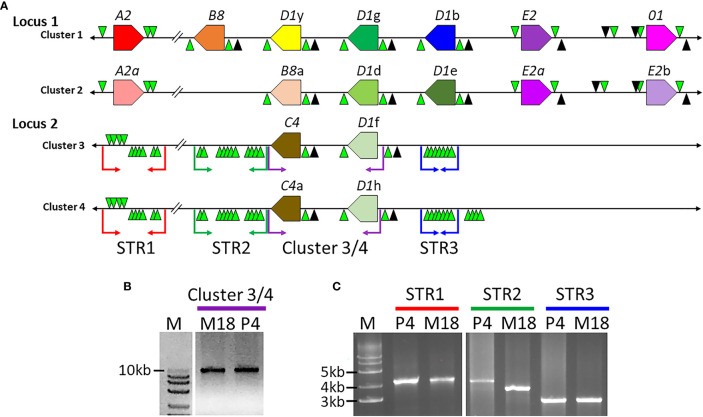
The structure of the *SpTrf* gene loci. **(A)** A representative map of the *SpTrf* loci. Locus 1 has allelic regions with unequal numbers of genes. Although Clusters 3 and 4 in Locus 2 appear identical, the different sizes of the flanking STR islands indicate that these two clusters are allelic. The colored polygons indicate the *SpTrf* genes located in the clusters with the pointed end of the polygon indicating the transcription direction. GA STRs (green triangles) and GAT STRs (black triangles) flank each gene and large GA STR islands flank Clusters 3 and 4 in Locus 2. The black horizontal line indicates the DNA extending from the 5ʹ and 3ʹ ends of the clusters. The colored angle-arrows in Locus 2 indicate the regions amplified by PCR and correspond to the colored bars over lanes in the DNA gels shown in **(B, C)**. **(B)** Clusters 3 and 4 in Locus 2 are the same size. The BAC templates for PCR are indicated above the lanes as M18 (BAC 10M18; Cluster 3) or P4 (BAC 3104P4; Cluster 4). Amplicons of Clusters 3 (BAC 10M18) and 4 (BAC 3104P4) indicate identical size. **(C)** Clusters 3 and 4 in Locus 2 have varying sizes of large GA STR islands. PCR was carried out for the P4 and M18 BAC clones to amplify the GA STR islands. M indicates the all-purpose Hi-Lo DNA marker (BioNexus), and sizes of the relevant bands are indicated to the left in **(B, C)**.

Previous work defined three *SpTrf* gene clusters from BAC insert sequence analysis, of which Clusters 1 and 2 were defined as allelic based on the nearly identical sequences of the genomic regions that flank these two *SpTrf* gene clusters (7). The allelic region for Cluster 3 was not reported because the two tightly linked *SpTrf* genes in Cluster 3 were present in both BAC 10M18 and BAC 3104P4 and were reported as replicates of the same region of the genome (7). This assumption was feasible given the 25X coverage of the BAC library (56). To verify whether these two BAC inserts were identical or allelic, the sequences were re-evaluated by dot plot comparison followed by verification using PCR amplification of the gene clusters and three large flanking STRs. The two *SpTrf* genes on BACs 10M18 and 3104P4 encompassed about 10 kb, which was verified by PCR, and had identical sequences based on comparisons using GenePallete (**Figure 1A**, purple angle arrows; **Figure 1B**). Three large GA STR islands were associated with the gene clusters based on GenePallete (**Figure 1A** and **Table 1**). PCR amplicons of the STRs indicated different sizes for the STR2 amplicon for the two BACs (**Figure 1C**). This suggested that BAC 10M18 (Cluster 3) and BAC 3104P4 (Cluster 4) were likely allelic and were identified as Locus 2 for the *SpTrf* gene family in the sea urchin genome.

**Table 1:** The second STR island in Locus 2 alleles are different lengths^1^.

BAC	Cluster	Size (nt)^2^		
		STR1	STR2	STR3
**10M18 (M18)^3^**	3	4293	3869	2633
**3104P4 (P4)**	4	4314	3998	2635

^1^These are results from sequence comparisons using GenePallet.

^2^The locations of the STR islands are shown in **Figure 1A**.

^3^Abbreviations for BAC numbers in parentheses correlate with **Figure 1C**.

#### Cluster 1 and Cluster 2 Have an Intergenic Region of Dissimilarity

**Figure 2:**
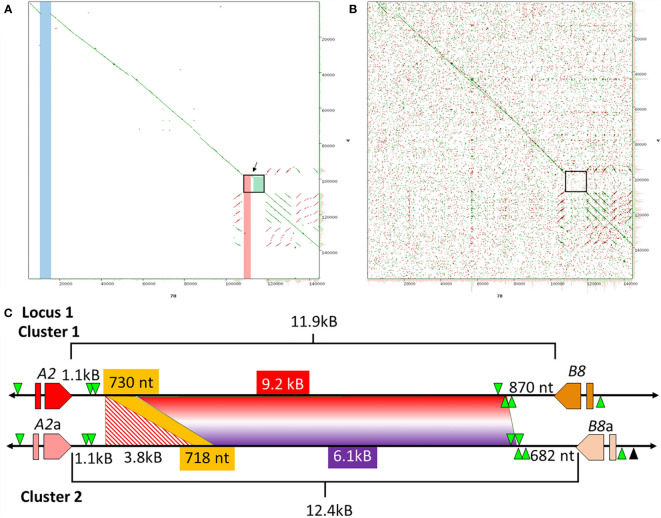
The IGRs of Clusters 1 and 2 in Locus 1 include non-matching sequences. **(A, B)** Dot plots show the comparison between Cluster 1 and Cluster 2. The dot plot in **(A)** employed a preset e-value threshold of 10 whereas the dot plot in **(B)** employed an e-value threshold of 10,000. The central diagonal in the dot plots indicate the mostly identical sequence of the allelic regions, while lines offset from the central diagonal indicate repeats that are highly similar in either a tandem (green) or inverted (red) orientation. Highlighted, colored vertical bars in **(A)** indicate the locations of mismatched sequences between the two clusters. The blue and red bars show the locations of sequences in Cluster 2 that are absent from Cluster 1 and the green box indicates a region of complete dissimilarity. The arrow between the red bar and green box in **(A)** indicates a region of similarity that is located between the two regions of dissimilarity. The black boxes in **(A, B)** are expanded in **(C)** to show details. YASS^8^ was used to generate dot plots with standard parameters (scoring matrix = +5, -4, -3 -4: composition bias correction: gap costs = -16, -4: X-drop threshold = 30). **(C)** The IGRs located between the *A2/*a and *B8/*a genes in Clusters 1 and 2 are a mixture of similar and dissimilar sequences. The red and orange polygons indicate the *SpTrf* genes, *A2*/a and *B8*/a genes, that flank these IGRs. GA STRs (green triangles) surround each gene. The horizontal black line indicates the DNA that extends from the 5ʹ and 3ʹ ends of each gene. The lengths of the IGRs between the *A2/*a and *B8/*a genes are indicated by upper and lower brackets. The sizes of the areas within the IGRs are indicated by colors that are coordinated when similar. The red and white striped region is a sequence that is only present in Cluster 2 and corresponds to the red bar in **(A)**. The yellow region is a short area of similarity, and the area of complete dissimilarity is shown as a polygon of a red and purple gradient. This figure in not drawn to scale.

Previous analysis of Clusters 1 and 2 of Locus 1 (**Figure 1A**) report different numbers of *SpTrf* genes, of which some genes are unique to a particular cluster based on different element patterns (7). Cluster 1 (BAC 10B1) has seven *SpTrf* genes while Cluster 2 (BAC 10K9) has six (7, 30). However, the flanking regions of these two allelic regions show approximately 99% sequence identity, which was the basis for reporting their allelic relationship rather than as two different loci (7). Dot plots of the BAC inserts for Locus 1 verified their allelic status, but also identified regions with significant sequence variations in the intergenic regions (IGRs) and in the flanking regions that surround the clusters (**Figure 2**). Although most of the region flanking the clusters generally aligned, Cluster 2 had a large deletion (**Figure 2A**, blue bar), in agreement with the previous report (7). The IGRs between the *A2* and *B8* genes in Clusters 1 and 2 were different in size and sequence that remained evident after increasing e-value threshold for the dot plot (67) (**Figures 2A, B**). The sequence identity of this region of dissimilarity in the two clusters was 42% to 48.1% based on BLAST and BioEdit analysis, respectively. When these sequences were used to search for other matches in the sea urchin genome in the NCBI database only poor matches were identified with percent mismatches of ~45.7% identity (based on results using MEGA7). A more detailed analysis of the IGRs between the *A2* an *B8* genes showed that there were two discrete areas of variation (**Figure 2C**). The first was 3.8 kb that was only present in Cluster 2, which was flanked on both sides by regions of high similarity with Cluster 1 (**Figures 2A, C**; red stripes). At the 5ʹ end of the IGR near the *A2* genes in each cluster was 1.1 kb of non-coding sequence that included the GA STRs. In the 3ʹ direction was 718 nt in Cluster 2 that matched with 96% identity to 730 nt in Cluster 1 (**Figure 2C**, yellow). The second region of dissimilarity (<40% identity) was 6.1 kb (Cluster 2) and 9.2 kb (Cluster 1) and extended from the 718/730 nt region of similarity to the *B8* genes (**Figure 2C**). While Clusters 1 and 2 of Locus 1 show similarities within flanking regions, there were also large regions of dissimilarity outside and within the cluster, the largest variation being the IGRs between the *A2* and *B8* genes.

### Stop Codons and Untranslated Regions in the *SpTrf* Genes

**Figure 3:**
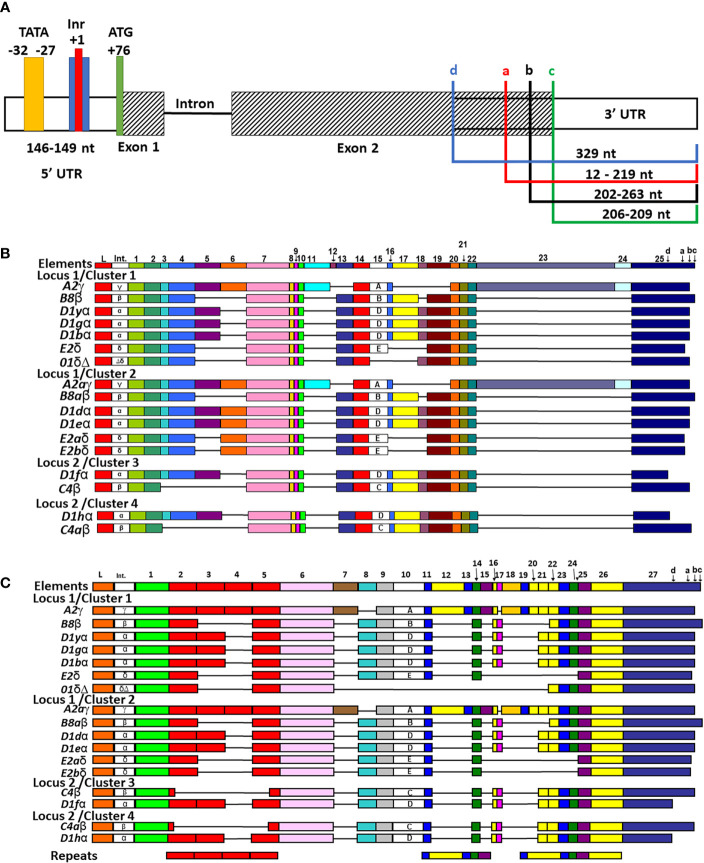
All *SpTrf* genes are in frame, have identifiable TATA, Inr sites, one or more stop codons, and most can be aligned with the previously established repeat-based and cDNA alignments. **(A)** A representative map of the genes shows the 5ʹ UTR, exons, intron, and 3ʹ UTR. The 5ʹ and 3ʹ UTRs are indicated by white rectangles, the two exons are indicated as striped rectangles, and the intron is indicated by a solid black line. The range in lengths of the 5ʹ UTR among genes is indicated. The four colored boxes in 5ʹ UTR indicate putative 5ʹ regulatory elements and their locations + or – of the conserved +1 A of the start transcription site (red). The TATA box (yellow), the Inr (blue), and the ATG translation start (green) are indicated. The 3ʹ UTR is variable in length among genes and is indicated by colored brackets showing the four possible locations of stop codons, which are labeled in lowercase ‘a’-‘d’. **(B)** The cDNA alignment of genes from the four clusters. The manual alignment was done in BioEdit by adding the genes in the clusters to a pre-aligned set of cDNAs and genes according to previous publications (12, 19). All possible elements are numbered at the top and the four possible stop codons are indicated in element 26. The leader (L), the intron (Int), elements (colored rectangles), and gaps (horizontal lines) are indicated for each gene. Intron type and subtype of element 15 are labeled within each respective rectangle. **(C)** The repeat-based alignment of the genes from the three clusters. The manual alignment was done as in **(B)** according to Buckley and Smith (19). All possible elements are numbered at the top and the four stop codons are indicated in element 27. The leader, intron, intron type, elements, subtype of element 10, and gaps are indicated as in **(B)**. The six types of repeats in the gene sequence are indicated by rectangles of identical color at the bottom.

The locations of the TATA box and polyadenylation site were reported previously for six of the *SpTrf* genes in Cluster 1 (30) except for the *01* gene, which was identified as part of Cluster 1 in a subsequent report (7). Those initial reports plus a set of partial *SpTrf* cDNA sequences (10) were used to identify or verify the transcriptional start and stop sites and the sizes of the untranslated regions for all of the *SpTrf* genes in the BAC insert sequences. Results showed that the 5ʹUTR ranged in size from 146 nt to 149 nt for 16 of 17 genes with the TATA box positioned 101 nt to 111 nt from the start codon within the 5ʹUTR, in agreement with the TATA box positioning described in Miller et al. (30) (**Supplementary Figure S5**). However, the TATA box for the *D1*g, which was reported to have a point mutation of TATACA was not verified. Rather, the *D1*g TATA box had a TATAAA sequence that was similar to the other genes, with the exception of *D1*d with a sequence of TATATA. No other conserved TATA box sites were identified within the proximity of the 5ʹ end of the UTR (the next nearest was distant by 1.3 kb). A putative initiator (Inr) (76, 77) was identified in all genes and located 27 nt to the 3ʹ side of the TATA box with the degenerate sequence of T(CA)A(+1)GTT in which the +1 A was conserved (**Figure 3A** and **Supplementary Figure S5**). This sequence is similar to the Inr sequence in *Drosophila* genes (76) and is considered a core promotor similar to the TATA box that can enhance binding affinity to a promotor element for either RNA polymerase or a transcription factor and, in some cases, can direct transcription without a functional TATA box (78).

3′UTRs are defined by the location of the stop codon and the polyadenylation sequence. Three stop codons have been reported for the *SpTrf* genes (19) and cDNAs (11, 12) and are present in the last element of exon 2 (**Figure 3A**, indicated as a, b, and c). Analysis of the genes in Clusters 3 and 4 identified a fourth stop codon in the *D1*f and *D1*h genes, in which a SNP at nucleotide 955 changed a tryptophan codon to a stop (**Figure 3A**, identified as d; **Supplementary Figure S6**). This increased the size of the 3ʹ UTR by 116 nt and decreased the length of exon 2 shortening the protein by 38 amino acids (aa) relative to the other *D1* genes. Two types of polyadenylation sequences were identified, AATAAA and ATTAAA, of which most genes [13 of 17] had both (**Table 2**). Overall, the 3′UTR varied in length from 195 nt to 357 nt primarily based on the positions of the stop codons among the genes (**Figure 3A**). All of the *SpTrf* genes appeared to be functional with short UTRs, although the *D1*d gene in Cluster 2 had different sequences for transcription initiation and for the location of transcript trimming prior to polyadenylation. These results suggested that these genes have the minimal requirements for expression, although the regulatory regions for these genes have not been evaluated.

**Table 2:** The 3ʹand 5ʹUTRs for the *SpTrf* genes are short and all have conserved and identifiable transcription elements.

Gene	Full length transcript (nt)	5’UTR (nt)	Exon 1 (nt)	Intron (nt)	Exon 2 (nt)	3’UTR (nt)	Poly-A site^1^	Poly-A site variant^2^
***A2***	1893	148	54	413	1418	273	257	12
***B8***	1422	149	54	415	1023	196	206	N/A^3^
***D1*y**	1503	148	51	411	1080	224	202	251
***D1*g**	1511	148	51	412	1080	232	N/A	259
***D1*b**	1511	148	51	411	1080	232	N/A	259
***E2***	1313	147	54	408	822	290	219	267
***01***	1197	148	51	348	771	227	208	167
***A2*a**	1898	147	54	407	1431	266	248	13
***B8*a**	1421	149	54	413	1023	195	209	N/A
***D1*d**	1522	148	51	412	1080	243	263	214
***D1*e**	1522	148	51	410	1080	243	215	264
***E2*a**	1260	147	54	406	822	237	218	266
***E2*b**	1237	148	51	407	810	228	208	167
***C4***	1725	149	54	383	909	230	205	N/A
***D1*f**	1931	146	54	411	963	357	329	378
***C4*a**	1725	149	54	383	909	230	205	N/A
***D1*h**	1931	146	54	411	963	357	329	378

^1^Nucleotide position of Poly-A site sequence (AATAAA) relative to the stop codon.

^2^Nucleotide position of Poly-A site sequence (ATTAAA) relative to the stop codon.

^3^N/A, not applicable, no Poly-A site or Poly-A site variant were found.

### Exon 1 Is Conserved Whereas Exon 2 Is Highly Variable Among the Genes

Exon 1 in all *SpTrf* genes are either 51 or 54 nt in length and encode a conserved signal sequence of 18 or 19 aa (12, 19, 30). The difference is the presence or absence of the second codon for glutamic acid (**Supplementary Figure S7**), which has been reported previously (11, 12, 19). Eight additional variations in exon 1 were identified among the 17 genes in the four clusters, all of which were nonsynonymous polymorphisms that maintained the non-polar characteristic of the encoded leader. Although the function of the leader has not been tested formally, it is predicted to have characteristic hydrophobic and alpha helical structure (12, 18), which is consistent with secretion of the SpTrf proteins (16) and/or their localization to the plasma membrane (13). Overall, exon 1 of the genes in the four clusters was highly conserved both in sequence and hydrophobicity and did not show extensive sequence variation.

**Figure 4:**
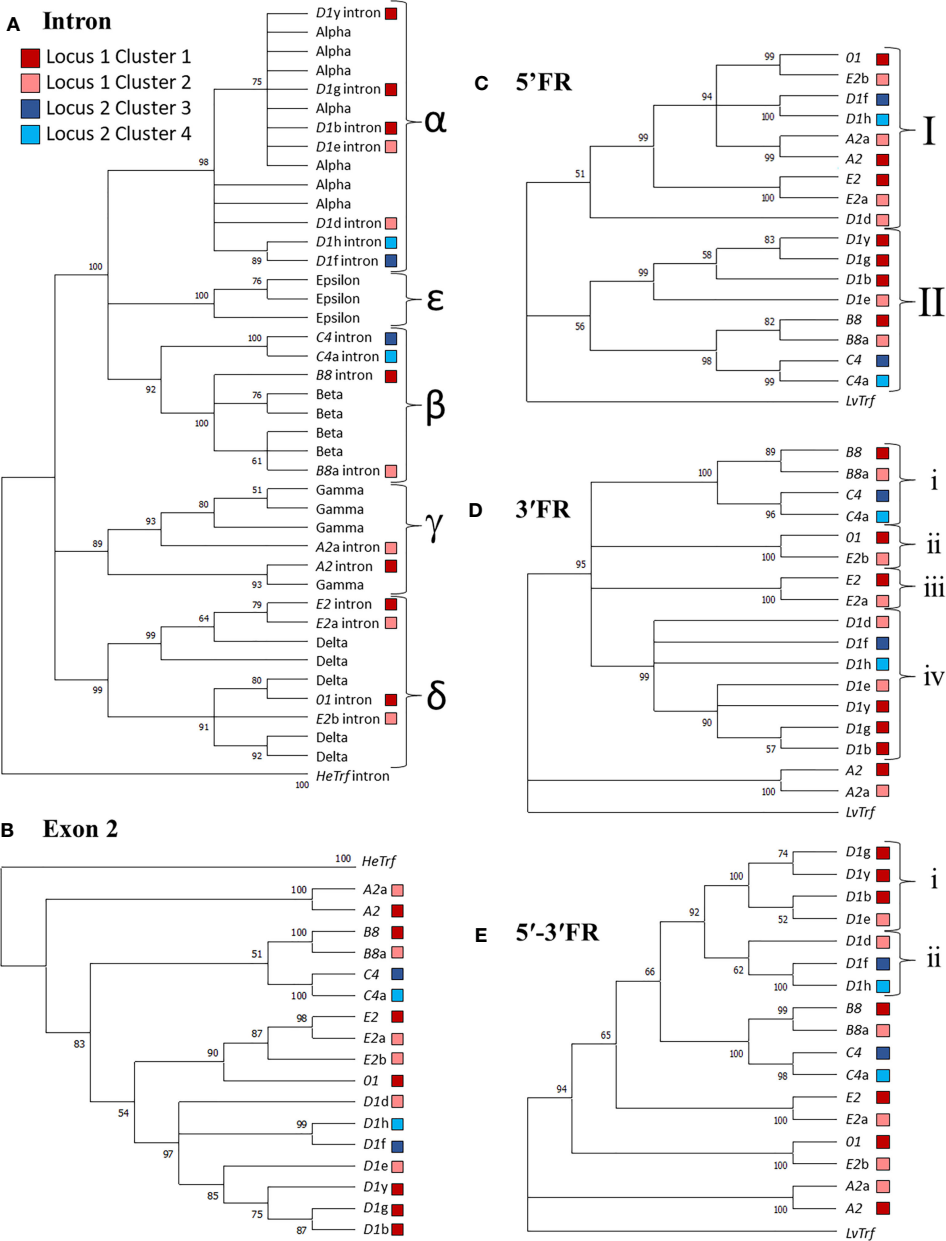
Sequence similarities among the *SpTrf* genes and their putative evolutionary relationships are revealed by similar structures of maximum likelihood trees. Alignments were performed with PRANK, and Phylogenetic analysis was completed in MEGA7. Phylogenetic trees were generated using three approaches: neighbor joining, maximum parsimony (see **Supplementary Figures S1–S4**), and maximum likelihood (shown), all of which resulted in similar tree structure. Colored boxes shown in the legend indicate the cluster in which the gene is located. Bootstrap values from 500 iterations are indicated for each tree. **(A)** The intron tree. The intron types (indicated by α-ϵ labels for separate clades) for each gene was identified using a previously published alignment of introns (19) with the introns from *HeTrf* genes defined as the outgroup. **(B)** The exon 2 tree. Exon 2 from each gene was aligned and the exon 2 sequences from *HeTrf* genes were defined as the outgroup. **(C)** The 5ʹFR tree. The 5ʹFR for each gene was selected using GenePallete and corresponded to 400 nt upstream of the start codon. The 5ʹFR of the *LvTrf* gene was used as the outgroup. **(D)** The 3ʹFR tree. The 3ʹFR for each gene was selected using GenePallete and corresponded to 400 nt downstream of the stop codon. The 3ʹFR of the *LvTrf* gene was used as the outgroup. **(E)** The concatenated 5ʹ-3ʹFR tree. The 5ʹFRs and 3ʹFRs used in **(C, D)** were aligned and then concatenated prior to phylogenetic analysis. The concatenated 5ʹ-3ʹ FR of the *LvTrf* gene was used as the outgroup.

Manual alignments of exon 2 have been used previously because of the large gaps required to optimize the alignments, and these efforts have generated two possible alignment outcomes denoted as the cDNA alignment and the repeat-based alignment that are feasible because of the variety of repeats in exon 2 (**Figures 3B, C**) (11, 12, 19). To evaluate exon 2 for the 17 genes in the four clusters, the sequences were added to previously published alignments to understand how the genes in the clusters were related to the other *SpTrf* sequences including their element patterns (**Figures 3B, C** and **Supplementary Figure S6**) and intron types (**Figure 4A** and **Supplementary Figure S8**) (7, 12, 19, 30).

#### The *A2* Genes

The *A2* and *A2*a genes in Locus 1, as reported previously (7, 30), have 25 of 27 elements according to the repeat-based alignment and are only missing elements 8 and 17 that encode histidine rich regions of the proteins (**Figure 3C**). Sequence comparison of exon 2 for the *A2* genes showed that they were not identical (93% identical, 100% coverage; **Table 3**) because of an indel of 15 nt starting at nucleotide 950 of the A2 gene alignment (**Supplementary Figure S9**). Additional differences in the coding region for the *A2* genes were due to 12 SNPs, of which 10 changed the amino acid and seven changed either the charge or pI of the amino acid (**Supplementary Table S2**). The percent identity of the full-length *A2* genes, including the intron was 88%, in agreement with the minimum percent identities among all genes (19). Exon 2 had a 98% identity, and exon 1 had a 95% identity between the *A2* and *A2*a genes indicating that the majority of the sequence differences were in the introns (**Supplementary Tables S3, S4**). The *A2* and *A2*a genes had moderately dissimilar (88% identity) γ type introns that were positioned in different sister subclades for γ introns in the phylogenetic tree of introns (**Figure 4A**). Differences in the introns were due to one or two nt indels in addition to a region of significant variation from nt 354 to the 3′ end of the intron (**Supplementary Figure S9**). The sequence variation among the *A2* γ introns was greater than the introns in most other *SpTrf* genes with the same element pattern and same intron type. This is not consistent with introns from genes that shared the same intron type, which tended to have highly similar introns (93% to 100% identical; **Supplementary Table S3**). Overall, the *A2* genes were highly similar but not identical, with most of their sequence variations located in the intron.

**Table 3:** Percent identity and coverage of the *SpTrf* genes of the same element pattern are highly similar*.

Genes compared		Coverage	Identity
***A2***	*A2*a	100	98
***B8*a**	*B8*	100	99
***C4***	*C4*a	100	100
***D1*f**	*D1*d	99	99
***D1*f**	*D1*e	99	98
***D1*f**	*D1*y	99	99
***D1*f**	*D1*g	99	98
***D1*f**	*D1*b	99	98
***D1*f**	*D1*h	100	100
***D1*h**	*D1*d	99	99
***D1*h**	*D1*e	99	98
***D1*h**	*D1*y	99	99
***D1*h**	*D1*g	99	98
***D1*h**	*D1*b	99	98
***D1*d**	*D1*e	100	98
***D1*d**	*D1*y	100	99
***D1*d**	*D1*g	100	98
***D1*d**	*D1*b	100	98
***D1*e**	*D1*y	100	99
***D1*e**	*D1*g	100	99
***D1*e**	*D1*b	100	99
***D1*y**	*D1*g	100	99
***D1*y**	*D1*b	100	99
***D1*g**	*D1*b	100	99
***E2***	*E2*a	100	99
***E2***	*E2*b	97	96
***E2*a**	*E2*b	97	96
***01***	*E2*	82	96
***01***	*E2*a	82	96
***01***	*E2*b	85	97

*These data were generated using NCBI BLAST.

#### The *B8* Genes

*SpTrf* gene sequence analysis from 10 sea urchins indicates that the genes with a *B* element pattern are likely common in the population (19) and that the gene copy number estimate for *B* genes in individual animals ranges from one to six (7). Two *B8* genes are present in the BAC insert sequences; *B8* in Cluster 1 (30) and *B8*a in Cluster 2 (7) based on the elements defined by the repeat-based and cDNA-based alignments (**Figures 3B, C** and **Supplementary Figure S6**). The *B8* genes did not show new variation in their element pattern relative to previous reports of other *B8* cDNAs and genes (11, 12, 19). The full-length gene sequences for *B8* and *B8*a were 99% identical including the β introns (**Figure 4A**, **Table 3** and **Supplementary Tables S3, S4**), in agreement with previous results (30). Differences between the sequences showed 10 SNPs of which five were located in the exons and four altered the charge or pI of the encoded amino acid (**Supplementary Table S2**). A single nt indel was located in the intron at position 71 (**Supplementary Figure S10**), and both *B8* genes had a single stop codon in the ‘c’ position in element 27 (**Figure 3A**). Overall, the *B8* genes in Cluster 1 and 2 were highly similar with a few differences in SNPs and were consistent with *B8* sequences previously reported.

#### The *C4* Genes

Two *C* genes were identified in Locus 2, *C4* in Cluster 3 (7) and *C4*a in Cluster 4 (**Figure 1A**). The *C* genes are not common in genomic DNA from individual sea urchins (19), however an estimate of gene copy number suggests 1 to >5 in 9 of 10 sea urchins (7). The *C4* genes had identical sequences (**Table 3**, **Supplementary Tables S3, S4** and **Supplementary Figure S11**) and matched with 97% identity over a 92% coverage to *Sp0376* cDNA [GenBank accession number DQ183179.1 (12)], which is the only *C4* sequence in the *SpTrf* sequence database (**Supplementary Figure S12**). The *C4* genes contained a distinguishing deletion in the type 1 repeat region of exon 2, which made them distinct from genes with the *C2*, *C3*, and *C5* element patterns (12, 19). This deletion brought together the first 10 nt of element 2 and the last 35 nt of element 5 and maintained the reading frame (**Figure 3C**). The stop codons for both *C4* genes were in the ‘b’ position in element 27 (**Figure 3A**). Although all *C* genes previously sequenced from genomic DNA had α introns (19), the *C4* and *C4*a genes on Clusters 3 and 4 had β introns based on the phylogenetic intron analysis (**Figure 4A**). The intron alignment and the intron phylogenetic tree indicated that the *C4* intron sequence shared similarity with the *B8* intron from the 5′ end of the intron to nt 285 and from nt 373 to 450 at the 3′ end. However, the central region, from nt 286 to 372, shared similarity with the γ intron in the *A2*a gene, although it had an indel of 32 nt (**Supplementary Figure S13**). The fragments of shared intron sequence between the *C4* genes and a gene with a different element pattern was unique among the *SpTrf* genes in the genomic clusters. The *C4* genes in Locus 2 of the *SpTrf* gene family were distinct from the other *C* genes based on both exon sequence and intron type.

#### The *D1* Genes

There are three *D1* genes in Cluster 1 known as *D1-*yellow (*D1*y*)*, *D1*-green (*D1*g), and *D1*-blue (*D1*b) (30), two *D1* genes in Cluster 2 known as *D1*d and *D1*e, and in Cluster 3 as *D1*f (7). Here, we report the *D1*h gene in Cluster 4 (**Figure 1A** and **Supplementary Figure S14**). All of the *D1* genes were highly similar (95% to 100% identical) in both the coding regions and the α introns with most of the differences identified as SNPs throughout the sequences (**Figure 4A**, **Table 3** and **Supplementary Figure S14** and **Supplementary Tables S3, S4**). The *D1*f and *D1*h genes had more SNPs compared to the *D1* genes in Locus 1, including a stop codon at nt 955 in position ‘d’ (**Figure 3A** and **Supplementary Figure S6**). The *D1* genes made up the largest group of genes in the *SpTrf* gene family (19) and were the most common element pattern in the sequenced BAC inserts as reported here and previously (7, 30).

#### The *E2* and *01* Genes

The *E* genes are as abundant as the *B* genes based on gene sequences identified from genomic DNA of individual sea urchins (19), and all sea urchins have at least one *E* gene copy with most predicted to have two to four and some as many as six copies (7). The *E* genes are the most highly expressed of the *SpTrf* gene family composing 546 of 689 cDNAs reported previously (11, 12). One *E* gene is present in Cluster 1, and two, *E2a* and *E2b*, are in Cluster 2 (7, 30). It is noteworthy that the allele position corresponding to *E2b* in Cluster 2 is the *01* gene in Cluster 1 rather than an *E2* gene. All *0* genes that have been identified from cDNA and gene sequences are named such because of a deletion of the key element used for naming (element 15 in the cDNA alignment or element 10 in the repeat-based alignment; **Figures 3B, C** and **Supplementary Figure S6**, blue box) (12). Hence, the allelic positioning of *E2*b and *01* has been noted as unusual. The alignment of the *E2* and the *E2a* genes indicated 99% sequence identity with a 100% coverage. In comparison, *E2*b was 96% identical to the other *E2* genes over a 97% coverage (**Table 3**). The decreased percent identity for *E2*b was due to a gap of 12 nt in the first type 1 repeat (element 2 as defined by the repeat-based alignment), and another of 15 nt in element 27 at the 3ʹ end of exon 2 (**Figure 3C** and **Supplementary Figure S15**). Strikingly, the second gap in *E2*b matched to an identical gap in the *01* gene on Cluster 1. Because of this sequence similarity and because the *01* gene was positioned in the same allelic location as *E2*b (**Figure 1A**), analysis of the *01* gene was included in the comparison among the *E2* genes. The *01* gene had a 96% to 97% identity (85% and 82% coverage, an outcome of the deletion described above) with the *E2* genes (**Table 3**). The element pattern of the *01* gene was similar to the *E2* genes and shared elements 1 to 6, however, unlike the *E2* genes, *01* shared elements 22, 23, and 24 with all of the other genes in both loci based on the repeat-based alignment (**Figure 3C**). An alignment of the *E2* and *01* genes showed that the only differences among the four genes was a region of 32 nt that was preceded by a gap of 90 nt (**Supplementary Figure S15**, yellow highlights). The *E2* and *01* genes all had δ introns (**Figure 4A**), although the *01* intron had a deletion of 60 nt making it the shortest intron among the *SpTrf* genes (**Supplementary Figure S15**, yellow highlights). The *E2* genes all had stop codons in the ‘a’ position, while *01* had a stop codon in the ‘b’ position (**Figure 3A**). Overall, the *E2* genes showed sequence similarity not only to each other but also to the *01* gene. In turn, the *01* gene had the highest level of similarity with the *E2*b gene, with which it appears to be allelic.

### The Majority of SNPs and Other Nucleotide Changes in Exon 2 Are Non-Synonymous

The *SpTrf* genes are expressed during sea urchin immune responses (10–12) and the encoded native proteins function as opsonins and augment phagocytosis (16). Genes that encode pathogen binding proteins are often under strong evolutionary pressure and selection from pathogen contact to optimize pathogen binding either to diversifying pathogens or to non-variable PAMPs. To determine whether the genes in the four clusters were diversifying at different rates relative to each other, the dN/dS scores were calculated among genes with the same element pattern (12). Comparisons among genes in these subsets of element patterns indicated both diversifying (dN/dS > 1) and purifying (dN/dS < 1) selection, although results did not typically vary by more than ±0.7 (**Table 4** and **Supplementary Table S5**). The two *A2* genes and the two *B8* genes had scores indicating purifying selection relative to each other suggesting that these alleles had not undergone much divergence. The average dN/dS value obtained for the *D1* genes (n = 7) varied depending on the analytic approach and was inconclusive (1.10565 from SLAC^10^ and 0.9402 from SNAP^9^) (**Supplementary Table S5**). dN/dS values calculated in SNAP suggested that each of the *D1* genes was diversifying differently, and when pairs of *D1* genes were compared, results showed that some were undergoing diversifying selection (dN/dS > 1; *D1*f, *D1*h, and *D1*g) while others were undergoing purifying selection (dN/dS < 1; *D1*d, *D1*e, *D1*y, and *D1*b) (**Table 4** and **Supplementary Table S5**). When nonsynonymous and synonymous nucleotide changes were identified from an alignment of exon 2 from genes with the same element pattern they showed a variety of SNPs with the majority resulting in nonsynonymous changes in exon 2 that changed the encoded amino acid by either charge or pI (**Supplementary Table S2**). These results suggested that the genes were diversifying or evolving, but at different rates.

**Table 4:** dN/dS values for genes with the same element pattern show that some are undergoing positive selection while others are undergoing purifying selection.

*Genes compared*		S	*N*	*aa^1^*	*Sd*	*Sn*	*dN/dS^2^*
*A2*	*A2*a	6	10	7	6	10	0.445545
*B8*	*B8*a	3	5	4	3	5	0.463235
*C4*	*C4*a	0	0	0	0	0	N/A^3^
*D1f*	*D1*d	2	12	8	3	13	1.248227
*D1f*	*D1*e	3	16	13	3.5	16.5	1.365854
*D1f*	*D1*y	3	12	10	3	12	1.148936
*D1f*	*D1*g	4	15	11	4	15	1.074074
*D1f*	*D1*b	5	12	9	5	12	0.686441
*D1f*	*D1*h	0	0	0	0	0	N/A
*D1h*	*D1*d	2	12	8	3	13	1.248227
*D1h*	*D1*e	3	16	13	3.5	16.5	1.365854
*D1h*	*D1*y	3	12	10	3	12	1.148936
*D1h*	*D1*g	4	15	11	4	15	1.074074
*D1h*	*D1*b	5	12	9	5	12	0.686441
*D1d*	*D1*e	5	17	13	5.5	18.5	0.961373
*D1d*	*D1*y	5	12	8	5	13	0.735849
*D1d*	*D1*g	6	16	11	6	17	0.803922
*D1d*	*D1*b	7	12	9	7	13	0.52349
*D1e*	*D1*y	2	7	5	2.5	7.5	0.857143
*D1e*	*D1*g	3	11	6	3.5	11.5	0.932432
*D1e*	*D1*b	4	7	4	4.5	7.5	0.473684
*D1y*	*D1*g	1	6	3	1	6	1.714286
*D1y*	*D1*b	2	2	1	2	2	0.285714
*D1g*	*D1*b	1	4	2	1	4	1.142857
*E2*	*E2*a	3	3	3	3	3	0.283871
*E2*	*E2*b	3	7	6	5	9	0.522556
*E2a*	*E2*b	2	9	8	4	11	0.801887

^1^The number of amino acid (aa) changes that encode either a change in polarity or pI.

^2^dN/dS values were generated for each gene both manually (first three columns) and by SNAP (last three columns). See **Supplementary Table S5** for more detailed calculations of these values.

^3^N/A, dN/dS values cannot be calculated.

### Phylogenetic Analysis Suggests Evolutionary Relationships Among the *SpTrf* Genes

Immune genes are often duplicated (reviewed in 37, 79) and the *SpTrf* gene family is no exception; duplicated genes are tightly clustered in discrete regions of the genome (11, 12, 19, 30). Given the nature of these genes and their function in sea urchin immune responses (16–18), attempts have been made to understand their theoretical evolutionary history (29). The previous analysis was limited to the exons and introns of the genes, and the six internal repeats in exon 2 because the sequences of the UTRs and IGRs were unavailable at the time. To address the question of *SpTrf* gene family evolution with the currently available sequence data, phylogenetic analyses were conducted for the *SpTrf* genes in the four clusters to evaluate the relationships among the 5ʹFR, the intron, exon 2, and the 3ʹFR. FRs were defined as sequences flanking both sides of the coding region that extended to the surrounding GA repeats and included the 5ʹ or 3ʹ UTRs. Sequences of the *Trf* genes from the sea urchin, *Heliocidaris erythrogramma* (*HeTrf*) (65), were used as the outgroup for analysis of exon 2 and the intron, while the 5′FR and 3′FR of the *Trf* sequences from the sea urchin, *Lytechinus variegatus* (*LvTrf*), were used as the outgroup for the 3ʹFR and 5ʹFR analysis. To date, *Trf* genes have been identified in six sea urchin species (65, 80–82), all of which are members of the Camarodonta order of euechinoids (83). Of these species, *Lytechinus* [LCA ~60 MYA (84)] and *Heliocidaris* [LCA ~75 MYA (84)] are not members of the Strongylocentrotid family (85) and therefore were appropriate choices as outgroups. The initial phylogenetic analysis of exon 2 from 138 *SpTrf* genes including those from the two genomic loci described here resulted in a polytomic tree structure that was an outcome of the large gaps required for optimal alignments (**Supplementary Figure S16**). Although this type of tree structure has been noted previously because of the mosaic element structure of exon 2, the structure was uninformative with regard to inferring evolutionary relationships among the *SpTrf* genes. Therefore, the dataset for exon 2 was decreased to only the genes in the clusters in an alternative approach to parse out putative relatedness among these genes. The resulting phylogenetic tree showed three major clades in which the earliest branch was composed of the *A2* genes, plus two sister clades that included a weakly supported cluster of the *B8* and *C4* genes, and a weakly supported cluster of the *E2*/*01* and *D1* genes (**Figure 4B**). Overall, the phylogenetic analysis of exon 2 suggested possible evolutionary relatedness among the genes. However, given the blocks of elements in exon 2, it was necessary to conduct additional detailed phylogenetic analyses to verify the pattern of the exon 2 tree. The phylogenetic tree for the introns initially employed to identify the intron types [see above (19)] was used to evaluate sequence similarities among introns from the four clusters with 39 introns from *HeTrf* genes (65) employed as an outgroup. The structure of the intron tree was composed of three strongly supported clades composed of δ introns, γ introns, plus a mixed clade of α, β, and ϵ introns (**Figure 4A** and **Supplementary Figures S2, S4**). The intron tree clearly identified the intron types for the *SpTrf* genes in the four clusters, which was in agreement with the previous report (19). The phylogenetic tree of the introns replicated the general structure of the tree for exon 2 (**Figures 4A, B**) strongly supporting the notion that genes with the same element pattern in exon 2 also share the same intron type.

The coding regions of immune genes are often poorly conserved either in sub-regions or throughout the coding regions because of host-pathogen arms race that drives selection for sequence diversification (9, 38, 45). Therefore, exon 2 may not be the optimal sequence to evaluate the relatedness among these genes. As an alternative approach to this problem, the FRs associated with the *SpTrf* coding regions were used in a phylogenetic analysis to avoid the variations in sequence and length for exon 2. This approach has been reported previously to understand the phylogeny of mini-genes encoding microRNAs (86). Phylogenetic trees of the 3ʹFRs and 5ʹFRs were generated in MEGA7 using PRANK alignments with the *LvTrf* 5ʹFR and 3ʹFR as the outgroup sequences. The 5ʹFR tree had two major clades in which Clade I consisted of the 5ʹFRs from the *01* gene, the *A2* genes, the *E2* genes, and three of the *D1* genes (**Figure 4C**). Clade II was composed of two sister sub-clades of which one contained the remaining *D1* 5ʹFRs and the second included the 5ʹFRs from the *B8* and *C4* genes. Although the bootstrap value was low for the node separating these two sister groups, this 5ʹFR tree structure was consistent with the structure of the intron tree (**Figure 4A**). The 3ʹFR tree showed good support for two major clades composed of the 3ʹFRs from the *A2* genes in one clade, and the 3ʹFRs from the other genes in the second clade. The 3′FRs from genes with similar element patterns clustered into four sub-clades composed of i) the *B8* and *C4* genes, ii) the *E2*b and *01* genes, iii) the *E2* and *E2*a genes, iv) and the *D1* genes (**Figure 4D**). Similarities among the structures of the phylogenetic trees for exon 2, and both of the FR trees indicated that FR sequences surrounding genes of the same element pattern were also similar and sufficiently different from those associated with genes of different element patterns to result in structural agreement among phylogenetic trees (**Figures 4A, C, D**).

Because of the variation in the structures of the 5ʹFR and 3ʹFR trees, a third assessment was carried out. The 5ʹFRs and 3ʹFRs (both ~ 400 nt in length) were aligned and then concatenated for each gene to generate the 5ʹ-3ʹFR alignment and tree (**Figure 4E**). Alternatively, the 5ʹ-3ʹFR sequences were concatenated and then aligned which gave tree structures that were essentially the same (data not shown). This was done to understand the possible evolutionary relationships among the genes without the coding and intron regions that may have affected or driven the outcome of tree topographies due to both length and sequence complexity of those regions of the genes relative to the short sequences of 5′FR and 3′FR. By analyzing the longer, concatenated 5ʹ-3ʹFR sequences, each nucleotide and each difference was weighted less in the final tree calculations. The 5ʹ-3ʹFR tree generated a more robust and definitive tree with regard to the sequence relationships among the genes (**Figure 4E**). Results showed that the 5′-3′FRs from the *D1* genes formed a single clade with two sub-clades (**Figures 4E, i, ii**) composed of i) the 5ʹ-3ʹFRs from the *D1*g, *D1*y, *D1*b, and *D1*e genes and ii) the *D1*d, *D1*f, and *D1*h genes. Unexpectedly, the 5ʹ-3ʹFRs from the *D1*e and *D1*d genes from Cluster 2 were separated into different sub-clades (**Figure 4E**, light red boxes). Furthermore, the 5ʹ-3ʹFR from *D1*d clustered with the 5′-3′FRs from *D1* genes in Locus 2 (**Figure 4E**, light red *vs*. blue boxes). The 5′-3′FRs from the *B8* and *C4* genes clustered together consistently and were sister to the *D1* clade, and the 5ʹ-3ʹFRs from the *E2* and *E2*a genes also clustered together. The overall structure of the 5ʹ-3ʹFR tree showed two sister clades with a ladderlike structure for the rest of the tree. The similarities in the structures of the three FR trees (**Figures 4C–E**) indicated that the *SpTrf* genes could be separated into two major groups in which the *D1, B8*, and *C4* genes may have had a shared evolutionary history, while the *E2, 01*, and *A2* genes may have undergone a separate evolutionary history.

### Percent Mismatches Highlight Sequence Similarities Among Genes of Different Element Patterns

**Figure 5:**
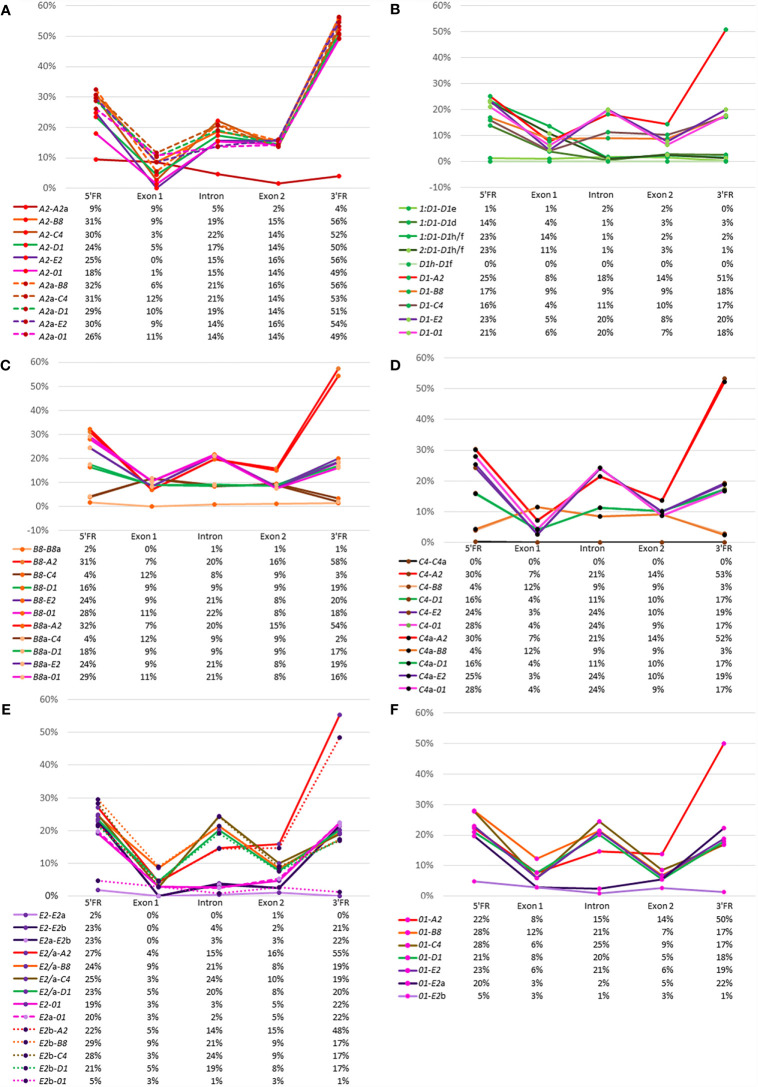
Relatedness among the genes can be inferred from percent mismatch scores for the 5ʹFR, exon 1, the intron, exon 2, and the 3ʹFR. Pairwise comparisons among all genes are shown for the FRs, exons, and the intron. The X axis in the graphs indicates the calculated percent mismatch score for each pair of genes and the Y axis indicates the region in the gene. Solid lines, dashed lines, and dotted lines are used to identify the pairs of genes compared. The color of the line corresponds to the color of the genes shown in **Figure 1A** with the exception of the *D1* genes, which are all shown as green lines. Below each graph is a table that gives the percent mismatch scores for each region graphed above. **(A)** The *A2* genes *vs*. other *SpTrf* genes. **(B)** The average percent mismatch of *D1* genes *vs*. other *SpTrf* genes. **(C)** The *B8* genes *vs*. other *SpTrf* genes. **(D)** The *C4* genes *vs*. other *SpTrf* genes. **(E)** The *E2* genes *vs*. other *SpTrf* genes. **(F)** The *01* genes *vs*. other *SpTrf* genes. Percent mismatch [pairwise distance/Ln^2^] was calculated from the pairwise distance matrix scores generated with MEGA7 using the PRANK codon alignment.

A complementary approach to using phylogenetic trees to derive evolutionary relationships among the *SpTrf* genes is to calculate the percent mismatch between pairs of genes. These values give a general view of gene sequence similarities and whether those similarities may be due to random chance or to true similarity. A similar analysis was reported using a pairwise distance matrix for the full-length genes that included the introns and four flanking regions [see Figure 9 in (30)]. Here, we used the same approach to analyze the 5ʹFR, exon 1, the intron, exon 2, and the 3ʹFR to reveal the relatedness between each gene with every other gene based on the pairwise distance scores (**Supplementary Table S6**). The results are presented as percent mismatch scores for easier visualization (**Figure 5**). The *A2* genes showed low percent mismatch scores against each other for the 5ʹFR, the intron, exon 2, and the 3ʹFR, with the 5ʹFR showing the greatest mismatch (**Figure 5A**, red line). Although the 5ʹFRs of the two *A2* genes showed higher mismatches of 18% to 30%, exon 1 showed a percent mismatch that was within the range of scores against the other *SpTrf* genes, which was consistent with the sequence conservation of this exon. The percent mismatch scores for the *A2* introns *vs*. other *SpTrf* introns (14-22%) were similar to the percent mismatch scores for exon 2 (15% ± 1%), however the mismatch scores for the 3ʹFR were much higher (48-57%) (**Figure 5A**). These data verified that the *A2* genes were similar to one another and were equally dissimilar to all the other *SpTrf* genes.

The *D1* genes in each of the four clusters had nearly identical percent mismatch scores among them. Hence, the percent mismatch scores were averaged for those *D1* genes in the two clusters in Locus 1, which reduced the complexity of the data. The two *D1* genes in Locus 2 were identical and analyzed as a single sequence termed *D1*h/f. Pairwise comparisons among the *D1* gene sequences showed very low percent mismatches for the intron, exon 2, and the 3ʹFR, whereas the mismatches for the 5ʹFR and exon 1 had greater variation (**Figure 5B**, green lines; **Supplementary Table S7**). The two *D1* genes in Cluster 2, *D1*e and *D1*d, had different percent mismatches for the 5ʹFR compared to the set of *D1* genes in Cluster 1, indicating sequence differences between the *D1* genes in the two clusters of Locus 1. Furthermore, the mismatches for the 5ʹFR among *D1* genes from different loci and mismatches with genes of different element patterns showed a similar range of variation (**Figure 5B**). When the *D1* genes were compared to genes with different element patterns, the percent mismatch scores varied among regions and element patterns. The *E2* and *01* genes (**Figure 5B**, purple and pink lines) showed relatively high percent mismatches against the *D1* genes for the 5ʹFR and the intron but intermediate scores for exon 2 and the 3ʹFR. Comparisons between the *D1* genes and the *B8* genes (**Figure 5B**, orange line) and the *C4* genes (**Figure 5B**, brown line) showed intermediate percent mismatch scores for the intron with scores for exon 2 and the 3ʹFR that were similar to the scores for the *D1* genes *vs.* the *E2* and *01* genes (**Figure 5B**).

The comparison between *B8* and *B8*a showed nearly identical low mismatch scores for all regions (**Figure 5C**), similar to results for the *D1* genes. The percent mismatch scores for exon 1 between the *B8* genes and the other genes were within the same range (7% to 12%). There were two outcomes for the percent mismatches for the introns of the *B8* genes compared to introns from the other genes, with a relatively high mismatch scores against the *A2*, *01*, and *E2* genes, and low scores against the *D1* and *C4* introns (**Figure 5C**). Interestingly, the percent mismatches for both *B8* genes compared to the *C4* gene were low for the 5ʹFR (**Figure 5C**, green and brown lines) along with the 5ʹFR against the *D1-*y, g, b, e genes (**Supplementary Table S7**). The mismatch scores for the 5ʹFR of the *B8* genes against the *A2, E2, 01*, and *D1*f/h/d ranged from 20% to 33%, whereas the percent mismatch scores for the 3ʹFR were lower for all genes (16% to 20%) except between the allelic *B8* genes and the *C4* genes (**Supplementary Table S7**). The percent mismatch scores for the *C4* genes compared to the other *SpTrf* genes showed similar results as that for the *B8* genes (**Figures 5C, D**). The lowest percent mismatch scores for the *C4* genes across all regions was against the allelic *C4* followed by the *B8* genes and the *D1* genes (**Figure 5D**). These results were in agreement with the phylogenetic tree results, which suggested that the *D1, B8*, and *C4* genes shared greater sequence similarity with each other than with the *E2, A2* and *01* genes.

The comparison between the *E2* and *E2*a genes showed low mismatch scores throughout the sequences of these alleles (**Figure 5E**, lavender line), and although the scores against the *E2*b gene were low for exon 1, the intron, and exon 2, higher mismatch scores were noted for both FRs (**Figure 5E**, dark purple lines). When the three *E2* genes were compared to the other *SpTrf* genes, all showed much higher percent mismatch scores for the intron and exon 2, except in the case of the *01* gene, which had low mismatch scores for exon 1, the intron, and exon 2 (**Figure 5E**, pink lines). Similar results were obtained when the regions of the *01* gene were compared to the other *SpTrf* genes (**Figure 5F**). The *01* gene had low percent mismatch scores at the 5ʹFR and the 3ʹFR against the same regions in the *E2*b gene but had much higher percent mismatches compared to the *E2* and *E2*a genes (**Figure 5F**, light purple *vs.* dark purple lines). These scores were comparable to scores for the 5FRʹ and 3ʹFR of the *E2* genes and *01* genes against the 5ʹFR and 3ʹFR for the other *SpTrf* genes (**Figure 5F**). The percent mismatch scores were consistent with the clustering of the *01* and *E2* genes, specifically with the *E2*b gene, in the phylogenetic trees (**Figure 4**). Overall, these results indicated sequence similarity between the *D1, B8*, and *C4* genes in all regions, similarity between the *E2* and *01* genes, and indicated that the *A2* genes were equally dissimilar to the other *SpTrf* genes in these clusters.

### A Modified Hypothesis for the Edges of the Segmental Duplications in the *SpTrf* Gene Clusters

**Figure 6:**
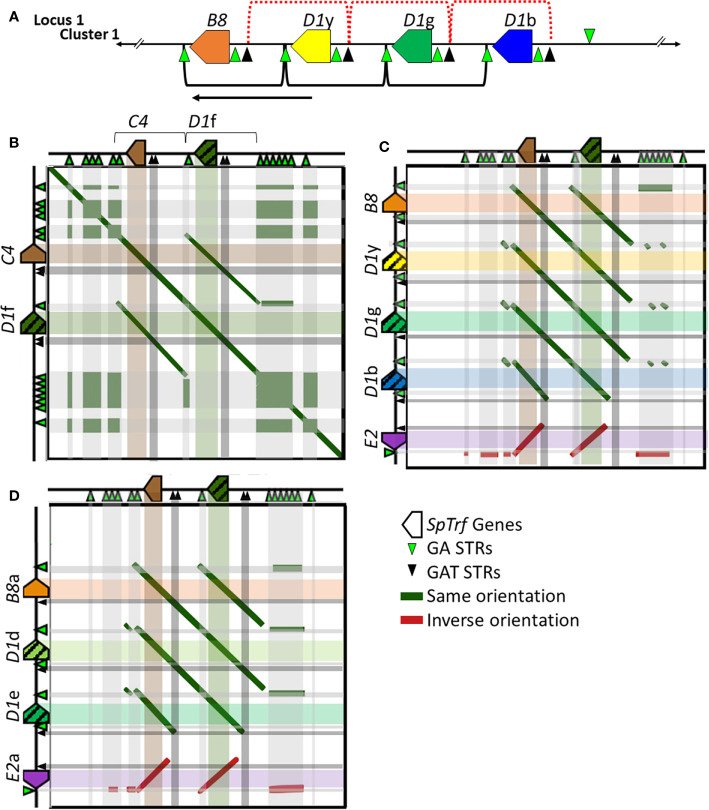
Representative dot plots of Cluster 3 compared to other *SpTrf* clusters indicates that the GA STRs are the likely edges of the segmental duplications in both loci. **(A)** A portion of Cluster 1 shows the region with the segmental duplications and the *D1* genes. The previously reported *D1* segmental duplications are indicated at the top of the figure [red dotted brackets (30)] and the revision to the proposed segmental duplications are indicated on the bottom (black brackets). The arrow indicates the directional shift of the proposed edges of the segmental duplications. **(B–D)** Representative images of a gene cluster or portion of a gene cluster are located to the left and top of each dot plot. Colored polygons indicate genes and transcriptional direction. Green triangles represent GA STRs and black triangles represent GAT STRs. The central diagonal in **(A)** shows the main alignment of cluster 3 against itself, while lines that are offset from the central diagonal in all dot plots indicate the locations of repeats or highly similar regions. Diagonal dark green lines indicate similar regions in the same orientation whereas, dark red solid lines indicate regions of inverse orientation. The highlighted horizontal and vertical lines of multiple colors (matching to the genes at the top or side) are added to the dot plots to illustrate the location of matched sequences. Dark green areas indicate the locations of GA STRs and dark gray areas indicate the locations of the GAT STRs. **(B)** Cluster 3 *vs*. Cluster 3. **(C)** Cluster 3 *vs*. a subset of genes in Cluster 1. **(D)** Cluster 3 *vs*. a subset of genes in Cluster 2. YASS^8^ was used to generate dot plots with standard parameters (scoring matrix = +5, -4, -3 -4: composition bias correction: gap costs = -16, -4: e-value threshold = 10: X-drop threshold = 30).

Tandem segmental duplications have been noted in the *SpTrf* gene clusters in Locus 1 based on dot plot analysis, phylogenetic analysis of intergenic segments, and calculations of pairwise sequence diversity between pairs of genes (7, 30). Previous reports based on dot plots indicate that the edges of the segmental duplications are the GAT STRs that surround and are positioned near the 3′ end of the *D1* and *E2* genes [**Figure 6A**, red brackets (30)]. However, with the addition of the *SpTrf* genes in Locus 2 (Clusters 3 and 4), the placement of the edges of the segmental duplications did not match the previously published results for Cluster 1 (30). Dot plot analysis of Cluster 3 compared to itself indicated that the two genes, *C4* and *D1*f, plus their flanking regions were very similar, suggesting a 2.7 kb segmental duplication (**Figure 6B** and **Supplementary Figure S17**, offset diagonals) in agreement with a previous report for the *D1* genes in Cluster 1 (30). Dot plots for the *C4*a and *D1*h genes in Cluster 4 showed identical results (data not shown). However, unlike the previous report, the 5ʹ end of the *D1*f/h segmental duplications were located at the large GA STR island (**Figure 6B**; see also **Figure 1A**, STR 2) and the 3ʹ end was located at the short GA STR near the 3ʹFR of the *D1*f/h genes. Similarly, the *C4*/a segmental duplications of 2.8 kb were positioned between the short GA STR near the 3′ side of the *D1*f/h genes and the 3ʹ end of the duplications were positioned near the large GA STR islands (**Figure 6B**, brackets and offset diagonals; see also **Figure 1**, STR 3). In these segmental duplications the GAT STRs (**Figure 6B**, black triangles and associated dark gray bars) were located in the center of the offset diagonals and therefore in the center of the segmental duplication rather than at the edges. These results suggested that the GA STRs rather than the GAT STRs defined the edges of the segmental duplications in Locus 2.

**Figure 7:**
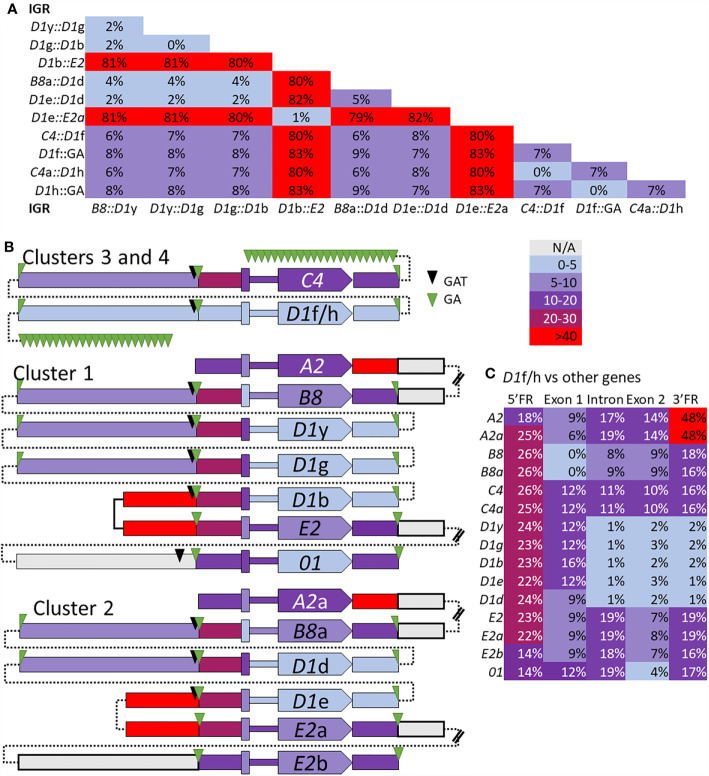
The percent mismatch between regions of the *SpTrf* genes suggests that the segmental duplications include the *B8* and *C4* genes with the *D1* genes. Alignments of the *B8*/a::*D1*y/d-IGRs, the *D1* IGRs, the *D1*b/e::*E2*/a-IGRs, and the *C4*/a::*D1*h/f-IGRs, plus the alignment of the 5ʹFR, exon 1, the intron, exon 2, and the 3ʹFR for all *SpTrf* genes were done with PRANK. **(A)** The pair-wise percent mismatch scores for IGRs indicate the level of sequence similarity. Percent mismatches were calculated from pairwise diversity scores in MEGA7 and are indicated with the color gradient legend. There are no mismatch scores between 30-40%. **(B)** A graphical representation shows levels of sequence similarities among genes and IGRs based on percent mismatch scores against the *D1*f/h genes. All genes are oriented in the same direction as indicated by the pointed polygon labeled with the gene name. From left to right across the figure are blocks that represent the IGRs, GA and/or GAT STRs, the 5ʹFR, the exon 1, the intron (narrow region), the exon 2 with the gene name, and the 3ʹFR. The thin dotted lines indicate how the sequences are linked together in their respective clusters and do not indicate sequence. The double bars in some IGRs indicate sequence that was not analyzed and is not shown. Percent mismatches for all blocks are color coded based on the gradient key. **(C)** The percent mismatch values for the regions of all *SpTrf* genes compared to the *D1*f/h indicates regions of similarity and dissimilarity. Results are color coded according to the gradient key.

The edges of the segmental duplications in Cluster 2 have been assumed to be the same as those in Cluster 1 based on the allelic status of these clusters (7). However, when dot plots were used to compare Locus 2 to Locus 1, a different outcome was identified relative to previous reports (7, 30). The dot plots of Cluster 3 compared to Clusters 1 or 2 indicated that the GA STRs were the most likely edges of the segmental duplications rather than the GAT STRs (**Figures 6C, D**). This redefined the edges of the segmental duplications for the *D1* genes in Locus 1 as GA STRs and indicated that they were the same size as reported previously (~4.5 kb). The new location of the duplications was a shift of 3 kb towards the end of the clusters in which the *A2* genes were positioned (**Figure 6A**, black brackets). The exception to this revised positioning of the segmental duplications in Locus 1 was the IGRs between *E2* and *D1*b in Cluster 1 and *E2*a and *D1*e in Cluster 2. The dot plot results indicated that the duplications terminated at the GAT STR located 5ʹ of the *D1*b and *D1*e genes (**Figures 6C, D** and **Supplementary Figure S17**), reducing the size of these particular duplications. To confirm the edges of the segmental duplications, alignments of the IGRs between linked genes was done using PRANK (IGRs were located between *B8*/a::*D1*y/d, the linked *D1* genes, *D1*b/e::*E2*/a, and *C4*/a::*D1*f/h) and percent mismatch scores were calculated. Results were ≤10% mismatch for the *B8*/a::*D1*y/d-IGRs, the *C4*/a::*D1*f/h-IGRs, and for all the IGRs between the linked *D1* genes (**Figure 7**, light blue and light purple). In comparison, the *D1*b/e::*E2*/a-IGRs in Locus 1 had ≥79% mismatch compared to the other IGRs indicating that they were not part of discernable segmental duplications (**Figure 7A**, red). Representative results for the percent mismatches between *C4*/a::*D1*f/h-IGRs and the other IGRs illustrated putative segmental duplications based on the results in the Locus 2 dot plots in which the edges of the duplication events were positioned at the GA STRs rather than the GAT STRs (**Figures 7B, C**, green *vs*. black triangles). These data suggested an alternative interpretation of the segmental duplications for this gene family and included the *B8* and *C4* genes in the duplication events with the *D1* genes, which had not been recognized previously.

### The Intergenic Regions Show Isolated Regions of Sequence Similarity

#### Small Regions of Shared Sequence Similarity Exist Among the IGRs Between the *A2*/a, *01*, and *D1* Genes

**Figure 8:**
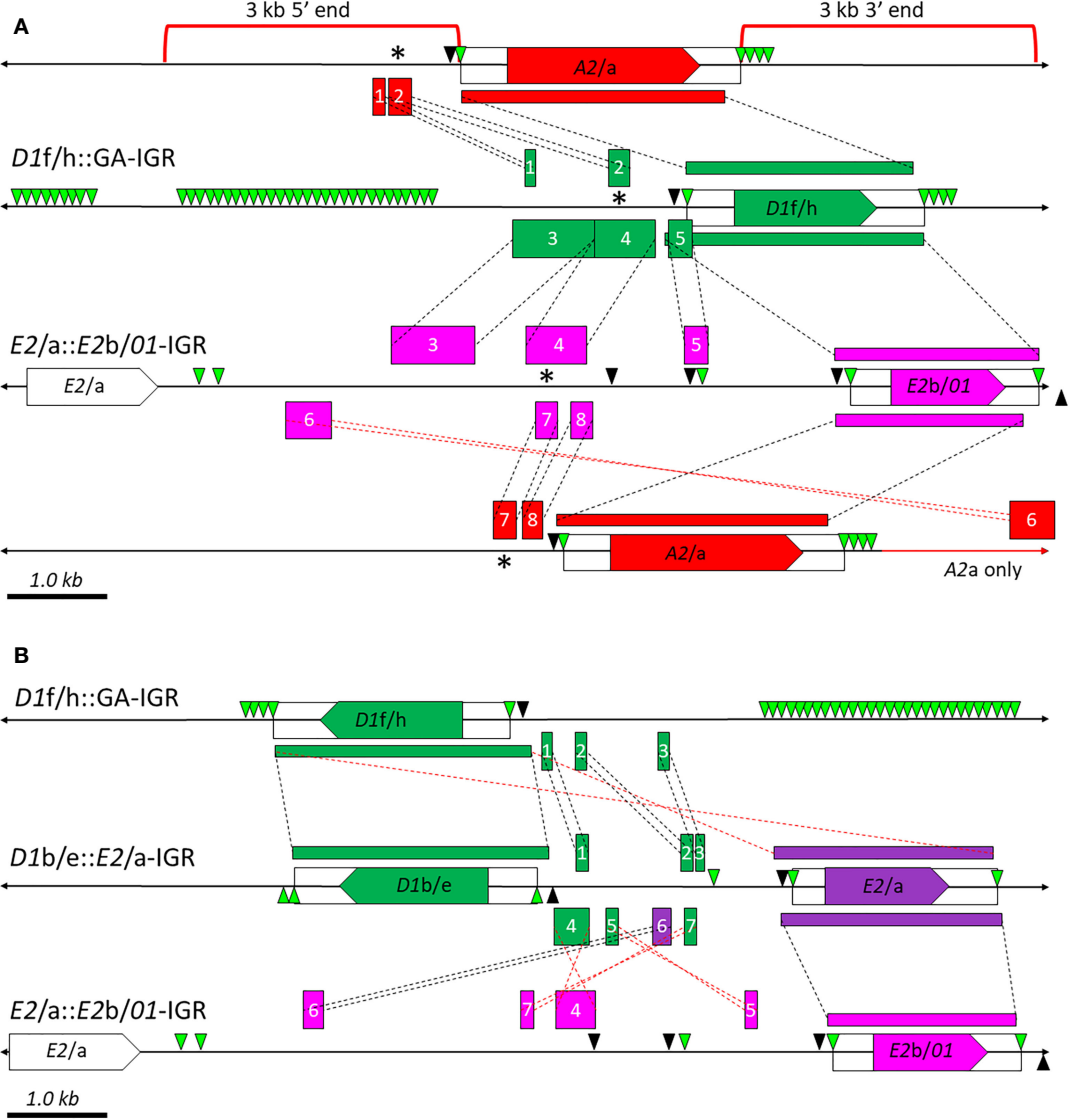
Comparisons the IGRs between the *A2*, *E2*, *01*, and *D1*f/h genes identify short regions of similarity. **(A)** The *D1*f/h::GA-IGRs and *E2*/a::*E2*b/*01*-IGRs are compared to each other, and both are compared to the 5ʹ and 3ʹ ends of the *A2*/a genes (indicated by red brackets). **(B)** The *D1*f/h::GA-IGRs, *D1*d/e::*E2*/a-IGRs, and the *E2*/a::*E2*b/*01*-IGRs are compared. Genes are indicated by the polygon labeled with the gene name and are colored according to **Figure 1A** and are flanked by UTRs (open boxes). The genomic DNA is indicated by horizontal black lines that passes behind the genes and includes the IGRs and flanking regions. Genes without color were not included in the analysis and are shown for orientation and comparison to **Figure 1A**. GA (green triangles) and GAT (black triangles) STRs are indicated. The colored boxes above and below the black horizontal line indicate regions of similarity as identified from dot plots from the YASS genomic similarity search tool set to a threshold of e^-20^. Areas of shared sequence among IGRs are numbered for clarity; see text for detailed description. Dotted lines connect the regions of similarity between IGRs including regions in the same (black lines) and inverted (red lines) orientation. This figure is drawn to scale. * indicates regions of similarity among all three alignments in **(A)**.

While the results presented above suggest an evolutionary relationship between the *D1*, *B8*, and *C4* genes and between the *E2* and *01* genes, there was little to suggest any sequence similarity outside of the coding regions between these two subsets of segmental duplications or with the *A2* genes in this gene family. To understand the evolutionary relationship between these two subsets of *SpTrf* genes and the *A2* genes, a region of 3 kb upstream of the 5ʹFRs and downstream of the 3ʹFRs of the *A2*/a genes (**Figure 8A**, red brackets) were compared to the i) IGRs between the GA STR islands and *D1*h/f genes (*D1*f/h::GA-IGRs) and ii) the IGRs between the *E2*/a genes and *E2*b/*01* genes (*E2*/a::*E2*b/*01*-IGRs) (**Figure 8A** and **Supplementary Figures S18A–D**). Dot plot analysis identified a 700 nt region in the 5ʹ end of the *A2*/a genes that contained two fragments (**Figure 8A**, red boxes 1 and 2) with sequence similarity to two separated regions in the *D1*f/h::GA-IGRs in which fragment 1 was positioned 1.4 kb from the 5ʹ end of the *D1*f/h genes (**Figure 8A**, green boxes 1 and 2). Fragment 2 was located 300 nt from the 5ʹ end of the *D1*f/h genes, similar to its location of 350 nt from the 5ʹ end of the *A2*/a genes. Fragment 1 was separated from fragment 2 by 730 nt in the *D1*f/h::GA-IGRs but was separated by only 30 nt in the 5ʹ end of the *A2*/a genes. Fragments 7 and 8 in the 5ʹ end of the *A2*/a genes were also identified in the *E2*/a::*E2*b/*01*-IGRs but were absent from the *D1*f/h::GA-IGRs (**Figure 8A**, red boxes 7 and 8). Fragments 7 and 8 were 3 kb from the 5ʹ end of the *E2*b/*01* genes and separated by 130 nt (**Figure 8A**, pink *vs*. red boxes 7 and 8). There were three regions of similarity between the *D1*f/h::GA-IGRs and the *E2*/a::*E2*b/*01*-IGRs (**Figure 8A**, green *vs*. pink boxes 3-5). Fragments 3-5 were larger than fragments 1 and 2 associated with the *A2*/a genes and together composed lengths of 1456 nt to 1483 nt. Fragments 3 and 4 were positioned next to each other in the *D1*f/h::GA-IGRs but were separated by 520 nt in the *E2*/a::*E2*b/*01*-IGRs (**Figure 8A**, green *vs*. pink boxes 3 and 4). Fragment 5 was 170 nt to 213 nt in length depending on the number of repeats in the GA/GAT STRs. This region was positioned within the *E2*/a::*E2*b/*01*-IGRs and matched to the GA/GAT STRs that made up the boundary of the 5ʹFR of the *D1*f/h genes. Fragment 5, which was associated with the *D1*f/h 5ʹFRs, also matched to the GA/GAT STRs that were located closer to the *E2*b/*01* genes and constituted the boundary of the 5ʹFRs. Only one region matched across all three regions (**Figure 8A**, indicated with an asterisk), which was fragment 2 or 7 in the 5ʹ end of the *A2*/a genes that was also identified within fragment 4 associated with the *E2*/a::*E2*b/*01*-IGRs and the *D1*f/h::GA-IGRs. No regions of similarity were identified to the 3ʹ side of the *E2*b/*01* genes compared to the other IGRs (not shown in **Figure 8**). However fragment 6 (**Figure 8A**, red box 6) was identified on the 3ʹ end of the *A2*a gene, which matched to a sequence located within the *E2*/a::*E2*b/*01*-IGRs and was positioned 730 nt from the 3ʹFRs of the *E2*/a genes. Fragment 6 was located 1350 nt from the 3ʹFR of the *A2*a gene and was inverted relative to fragment 6 associated with *E2*/a::*E2*b/*01*-IGRs. Fragment 6 was only identified in the 3ʹ end of the *A2*a gene and was missing from the 3ʹ end of the *A2* gene because this was a region of dissimilarity relative to the *A2* IGR (**Figure 2C**, red and white striped triangle). While the 5′ end of the *A2* gene and the *E2*/a::*E2*b/*01*-IGRs were not identical to the *D1*f/h::GA-IGRs that were indicative of duplications, there were small fragments of shared sequence. These short fragments of sequence confirmed that there was sequence similarity outside of the coding regions of these genes that linked the *D1*, *B8*, and *C4* segmental duplications with the *E2* and *01* duplications and with the *A2* genes.

#### There Are Fragmented Regions of Shared Sequence Similarity in the *D1*b/e::*E2*/a-IGRs

The shared sequence fragments in the 5ʹ and 3ʹ ends of the *A2*/a genes, in the *E2*/a::*E2*b/*01*-IGRs, and in the *D1*f/h::GA-IGRs suggested that shared sequences may also be identified for the IGRs between the *E2*/a genes and the *D1*b/e genes (*D1*b/e::*E2/*a-IGRs). These IGRs were of interest because the *D1*b/e genes were missing the 5ʹ end of the proposed *D1*/*B8*/*C4* segmental duplications based on results of dot plot comparisons to Cluster 3 (**Figure 6**), and because these IGRs were short (3.4 kb) (**Figure 8B**) and located to the 5ʹ side of the *D1*b/e genes and the *E2/*a genes. To understand the complexity of these IGRs, the *D1*f/h::GA-IGRs and the *E2*/a::*E2*b/*01*-IGRs were compared to the *D1*b/e::*E2/*a-IGRs (**Figure 8B**; **Supplementary Figures S18E, F**). Results from the dot plots of the *D1*b/e::*E2/*a-IGRs indicated three short fragments of similarity, 1 - 3, that were present in the corresponding *D1*f/h::GA-IGRs (**Figure 8B**, green boxes 1 - 3). These fragments were in the same orientation in both loci relative to the local *D1* gene. There were four short fragments of similarity, 4 - 7, located in the *E2*/a::*E2*b/*01*-IGRs and the *D1*b/e::*E2/*a-IGRs (**Figure 8B**, green and purple boxes 4 - 7). Of these four fragments, all but fragment 6 were in the same orientation as the local *D1*b/e genes, whereas fragment 6 was oriented the same orientation as the local *E2*b*/01* genes. This result, in addition to the dot plots (**Figure 6**) indicated that fragments 4, 5, and 7 were likely associated with the *D1* rather than the *E2* gene given that they were oriented in the same direction. However, the fragments 4, 5, and 7, which were in the same orientation as the *D1*b/e genes, were not positioned in the same order in the *E2*/a::*E2*b/*01*-IGRs indicating a possible sequence scrambling in this region. Taken together these data indicated that the regions between the *D1*b/e and the *E2*/a genes contained small fragments of sequence similarity in the IGRs of the *D1* genes and one small fragment that might be attributed to the *E2* genes. This was similar to the results for the *A2*/a analysis (**Figure 8A**). These results illustrated that, while the 5ʹ IGRs of these gene were not identical, there were short fragments of sequence similarity shared among them that would be consistent with genomic instability for both of the loci that harbor the *SpTrf* gene clusters. These shared regions may have implications not only to the relatedness among the genes but also among the IGRs.

## Discussion

### A Hypothetical Evolutionary History of the *SpTrf* Gene Family in the Sequenced Sea Urchin Genome

**Figure 9:**
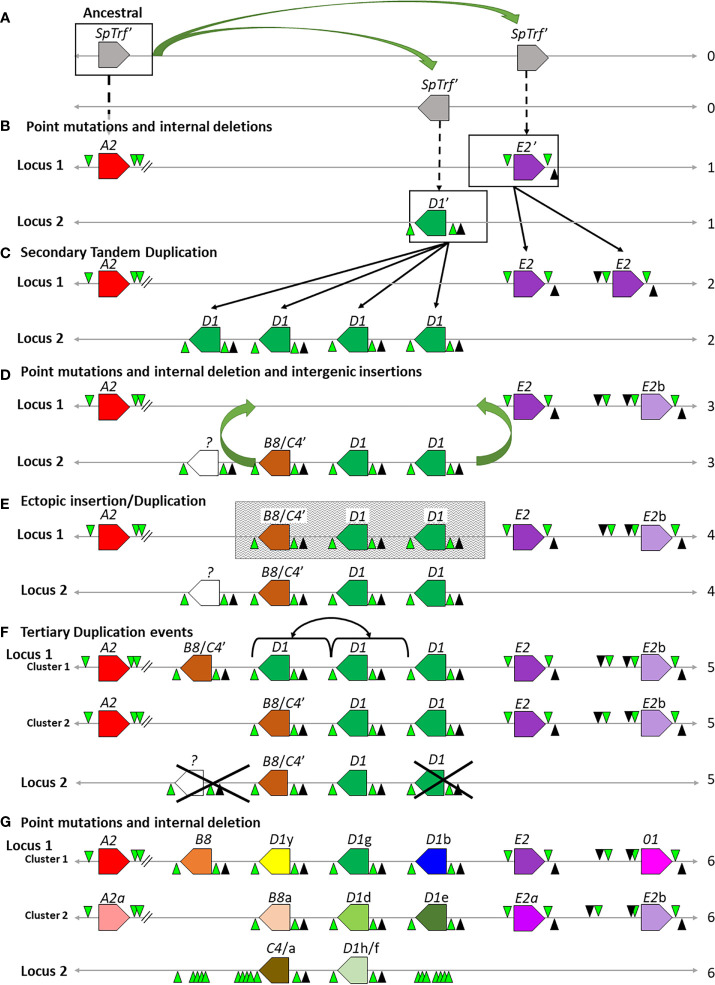
A model for the theoretical evolutionary history of the *SpTrf* gene clusters in the sequenced genome based on gene duplications, ectopic insertions, and deletions. Each step in this theoretical evolutionary history of the gene clusters is indicated on the right with numbers and labeled on the left **(A–G)**. Genes and their direction are indicated by colored polygons and labeled with the gene name. The prime (ʹ) associated with a gene name indicates a hypothetical LCA version of the gene. Gene polygons without color indicate genes that are proposed to exist but whose element pattern cannot be determined. Variations in or changes to gene colors indicate internal point mutations/insertions/deletions during the lineage of a particular gene. GA (green triangles) and GAT (black triangles) STRs are shown. The horizontal gray line indicates the IGRs that flank the genes. Open boxes surrounding the genes in **(A, B)** indicate edges of proposed duplication regions. Curved green arrows indicate duplications and ectopic insertions, dotted black arrows indicated the transition from an ancestral *SpTrf* gene to specific *SpTrf* gene lineages, straight black arrows indicate duplications of genes over time. The shaded box shows the ectopically inserted region in Locus 1. Brackets connected with double ended arrows indicate a recent duplication event. Large black Xs indicate gene deletions. This figure is not drawn to scale..

The necessity for diverse and constantly diversifying genes in the face of a broad array of pathogens leads not only to the generation of complex immune systems but to complex immune gene families. Duplications, insertions, inversions, meiotic mispairing, unequal crossing over, and gene conversion all have the potential to result in large and diverse immune gene families encoding proteins that keep pace in the arms race with the pathogens (20, 23, 24, 37, 38, 87). Based on the sequence relationships among the genes in the four clusters including their FRs and IGRs, we propose a hypothetical evolutionary history of how the *SpTrf* gene clusters were generated. The LCA *SpTrf*′ gene plus a portion of its 5ʹ and 3ʹ flanking regions is the starting sequence for this evolutionary history. *SpTrf*′ underwent initial duplications and ectopic insertions into the same locus and into a different region of the genome to establish a second locus (**Figure 9A**). These two loci subsequently underwent gene diversification to generate the ancestral *D1ʹ*, *E2ʹ*, and the *A2* genes (**Figure 9B**). The two loci containing the ancestral *D1ʹ* or *E2ʹ* genes underwent independent secondary duplication events, generating several tandem genes of the same element pattern and forming the initial clusters (**Figure 9C**). These gene duplicates acquired internal SNPs and indels thereby continuing sequence diversification (**Figure 9D**). One outcome was the sequence variation among the *D1* genes and the appearance of the ancestral *B8*/*C4ʹ* gene from *D1* duplications in Locus 2 (**Figures 9C, D**). The other outcome was the diversification of the *E2* genes to generate the *E2b* gene on Locus 1 (**Figures 9C, D**). Next, a larger duplication and ectopic insertion moved at least two *D1* genes plus the ancestral *B8*/*C4*ʹ gene from Locus 2 into Locus 1 that was positioned between the *A2* and *E2* genes (**Figures 9D, E**). This may have been the ancestral change that resulted in genes facing in both directions in Locus 1 and which scrambled the IGR sequences between the *D1* and the *E2* genes. The mismatch in the number of *D1* genes between Clusters 1 and 2 in Locus 1 is likely due to tertiary duplications that generated the *D1*y/g genes, which may have occurred either by a direct duplication of the *D1*y/g genes in Cluster 1 (**Figure 9F**) (30), or by an ectopic insertion from the allele in Cluster 2 (not shown). Finally, the individual *SpTrf* genes underwent further internal indels and SNPs generating the individual sequence variation among the genes, including the generation of the *01* gene from the *E2*b gene and the *B8* and *C4* genes from the *B8/C4*ʹ ancestor. The final outcome is the extant clusters and loci in the sequenced sea urchin genome (**Figure 9G**).

### Supporting Evidence for the Evolutionary History of the Extant *SpTrf* Family

The evolutionary history of the *SpTrf* gene family is based on the results presented herein. We speculate that the sequence of the LCA *SpTrf*′ gene had the majority of elements and the maximum number of repeats in exon 2 (**Figure 3C**), which subsequently underwent at least two duplications and ectopic insertion events (**Figure 9A**). This is based on alignments of the IGRs of the extant genes, which reveal a number of small regions of sequence similarity across all extant *SpTrf* genes reported here. We also hypothesize that an *SpTrf*′ gene with the maximum number of repeats in exon 2 would be the most parsimonious candidate gene sequence to generate other *SpTrf* genes, which are short genes with fewer elements, through deletions rather than vice versa through element or repeat duplication and diversification. The *A2* genes are an exception to this as previous research has proposed that *A2* genes have undergone a large duplication event in exon 2 that increased their size and gave them the designation of long genes (29). We hypothesize that the *A2* genes underwent a separate evolutionary history compared to the *E2*ʹ and *D1*ʹ genes after the duplication and ectopic insertions of the *SpTrf*′ (**Figures 9A, B**). The separate evolutionary history of the *A2*/a genes is based on the early branching position of the *A2*/a genes in the phylogenetic trees that infers a later divergence of the short *SpTrf* genes, and is based on the distant location of the *A2*/a genes in Locus 1 that are separated by non-conserved IGRs. This notion is consistent with a previous report speculating that long genes have unique type 1 repeats (see **Figure 3C**, type 1 repeats are shown as red rectangles) that underwent a separate evolutionary history from the type 1 repeats in the short *SpTrf* genes (29).

The similarities between the *E2*/a::*E2*b/*01*-IGRs and the *D1*f/h::GA-IGRs support the idea of a shared evolutionary history among the genes, which extends beyond the similarities of the coding regions and into the 3ʹ ends of the genes. The *E2*/a::*E2*b/*01*-IGRs in Locus 1 contain large regions that match to sequences in the *D1*f/h::GA-IGRs in Locus 2 that are also present in most of the *D1* segmental duplications. These matching regions are dispersed within the large *E2*/a::*E2*b/*01*-IGRs but are relatively contiguous in the *D1*f/h::GA-IGRs. This suggests that the *E2*/a::*E2*b/*01*-IGRs may have originally been similar in size to the *D1*f/h::GA-IGRs and underwent a number of insertion events to separate the regions of sequence similarity and elongate the IGRs to their current size (**Figures 9C, D**). On the other hand, when the *D1*f/h::GA-IGRs are compared to the *D1*b/e::*E2/*a-IGRs only short, fragmented regions of sequence similarity are identified. These short regions may have been the outcome of the proposed ectopic insertion of the *D1*/*B8*/*C4* region from Locus 2 into Locus 1 (see below; **Figures 9D, E**). This evolutionary history suggests that both the *D1* and the *E2* genes were both products of the *SpTrf*′ ancestral gene duplication that subsequently underwent separate evolutionary histories to generate the two subsets of *E2*/*01* and *D1*/*B8*/*C4* genes (**Figure 9**).

The sequence diversification of the *D1* genes, which are present in segmental duplications, are based on sequence analysis of the *D1* genes and their flanking regions. In agreement with Miller et al. (30), the *D1* genes appear to be a product of multiple recent duplication events that is also supported by our phylogenetic analysis and percent mismatch scores, which includes similarities among the FRs. However, based on our analyses, we hypothesize that the ancestral *D1*ʹ gene was most similar to the *D1* genes in Cluster 1 plus *D1*e in Cluster 2 (**Figures 9C–G**) because these genes are more similar to each other than to the remaining *D1* genes in either locus. This result is also consistent with purifying selection detected for the *D1*y/b/e genes and for diversifying selection for the *D1*f/h genes. Although the identity between *D1*f and *D1*h could be based on their location in Locus 2, a more in-depth analysis suggests a specific evolutionary relationship among the *D1* genes in the two loci, which is based on two levels of results. First is a hypothesized evolutionary relationship among the *D1* genes with the *B8* and *C4* genes. This is based on the sequence similarity among these genes along with the updated edges of the *D1* segmental duplications to include the *C4* and *B8* genes. Both the *B8* and *C4* genes may have once initially been a product of a *D1*ʹ gene that underwent diversification events in Locus 2 to generate a descendant LCA *B8/C4*ʹ gene (**Figures 9C, D**), along with duplications of an unknown number of additional *D1* genes, that would later go on to become the extant *C4* and *B8* genes. Although the number of duplicated *D1* genes that may have been present in Locus 2 is unknown, the large islands of GA STRs associated with this gene cluster may be the remnants of gene deletions (7). Secondly, there are indications that the *B8* genes and several *D1* genes in Locus 1 may have been the product of a duplication and ectopic insertion event from Locus 2 (**Figures 9D, E**). This idea is supported by the sequence similarity between the *B8* and *C4* genes, which are located in allelic positions in the two extant loci. A recent evolutionary history between the *B8* and *C4* genes is supported by phylogenetic analysis, percent mismatch scores, and dot plot analysis. The duplication of the *D1*ʹ and *B8*/*C4*ʹ genes in Locus 2 and the location of their insertion in Locus 1 (**Figures 9D, E**) is supported by the IGR sequences on either side of the *B8* and *D1* genes, which are either highly dissimilar (*A2*/a::*B8*/a-IGRs) or show signatures of sequence scrambling (*D1*b/e::*E2/*a-IGRs) (**Figures 9D, E**). The outcome of the ectopic insertion is a heterogeneous cluster of genes in Locus 1 the include both *D1* derived genes and *E2* derived genes that are present in opposite orientations (**Figure 9F**).

The appearance of the *E2* and *01* genes is proposed to have originated with the *E2*ʹ gene (**Figure 9B**). *E2*ʹ initially underwent a tandem duplication to form two *E2* genes in Locus 1 (**Figures 9B, C**). This was followed by sequence diversification of one of the *E2* genes into *E2*a and *E2*b in Cluster 1 and Cluster 2, respectively (**Figures 9C, D**). The *E2*b allele in Cluster 1 subsequently acquired multiple deletions that resulted in the *01* gene (**Figure 9G**) including a large deletion in exon 2 that maintained the reading frame either fortuitously or through unknown repair mechanisms (88). The evolutionary relationship between the *E2* and *01* genes is noteworthy because the sequence similarity between the *01* gene and the three *E2* genes has not been reported previously.

## Conclusion

Overall, the evolutionary history of this gene family suggests a number of duplications, deletions, insertions, conversions, and point mutations, all of which lead to the distinct clustering and sequence similarity among the members of this gene family (**Figure 9**). It must be noted, however, that this hypothetical evolutionary history is based on genes from a single sea urchin and that different sea urchins have been proposed to contain different repertoires of this gene family (8, 12, 19). Variations among *SpTrf* gene repertoires can be considered as a population level immunological benefit in an environment with many potential pathogens. Additional gene sequence data and cluster structure from other individual sea urchins will either clarify and verify this history or will expand the numbers of genes and their sequence variations to further illuminate the evolution of this gene family.

## Data Availability Statement

The datasets presented in this study can be found in online repositories. The names of the repository/repositories and accession number(s) can be found in the article/**Supplementary Material**.

## Author Contributions

MABH conceived of the project, generated the data, and wrote the paper. LCS acquired the funding and wrote the paper. All authors contributed to the article and approved the submitted version.

## Funding

This project was supported by funding from the Wilber V. Harlan Trust through the Department of Biological Sciences at GWU to MABH, and from the US National Science Foundation (IOS-1146124, IOS-1550474, IOS-1855747) to LCS.

## Conflict of Interest

The authors declare that the research was conducted in the absence of any commercial or financial relationships that could be construed as a potential conflict of interest.

## Publisher’s Note

All claims expressed in this article are solely those of the authors and do not necessarily represent those of their affiliated organizations, or those of the publisher, the editors and the reviewers. Any product that may be evaluated in this article, or claim that may be made by its manufacturer, is not guaranteed or endorsed by the publisher.

## Footnotes

1
http://www.genepalette.org

2
http://www.ncbi.nlm.nih.gov/orffinder

3
http://guidance.tau.ac.il/ver2/

4
http://wasabiapp.org/software/prank/

5
https://blast.ncbi.nlm.nih.gov/Blast.cgi

6
http://www.megasoftware.net

7
http://whis.caltech.edu/Echinobase/LvAbout

8
http://bioinfo.lifl.fr/yass/index.php

9
https://www.hiv.lanl.gov/content/sequence/SNAP/SNAP.html

10
http://classic.datamonkey.org/dataupload.php
