# Supplementary material for: Sequence Diversity, Locus Structure, and Evolutionary History of the SpTransformer Genes in the Sea Urchin Genome
Source: Front Immunol. 2021 Nov 15;12:744783. doi: 10.3389/fimmu.2021.744783 (PMC8634487; doi:10.3389/fimmu.2021.744783)
Supplement: Supplementary file 1 [file DataSheet_1.pdf]

Supplementary Figures

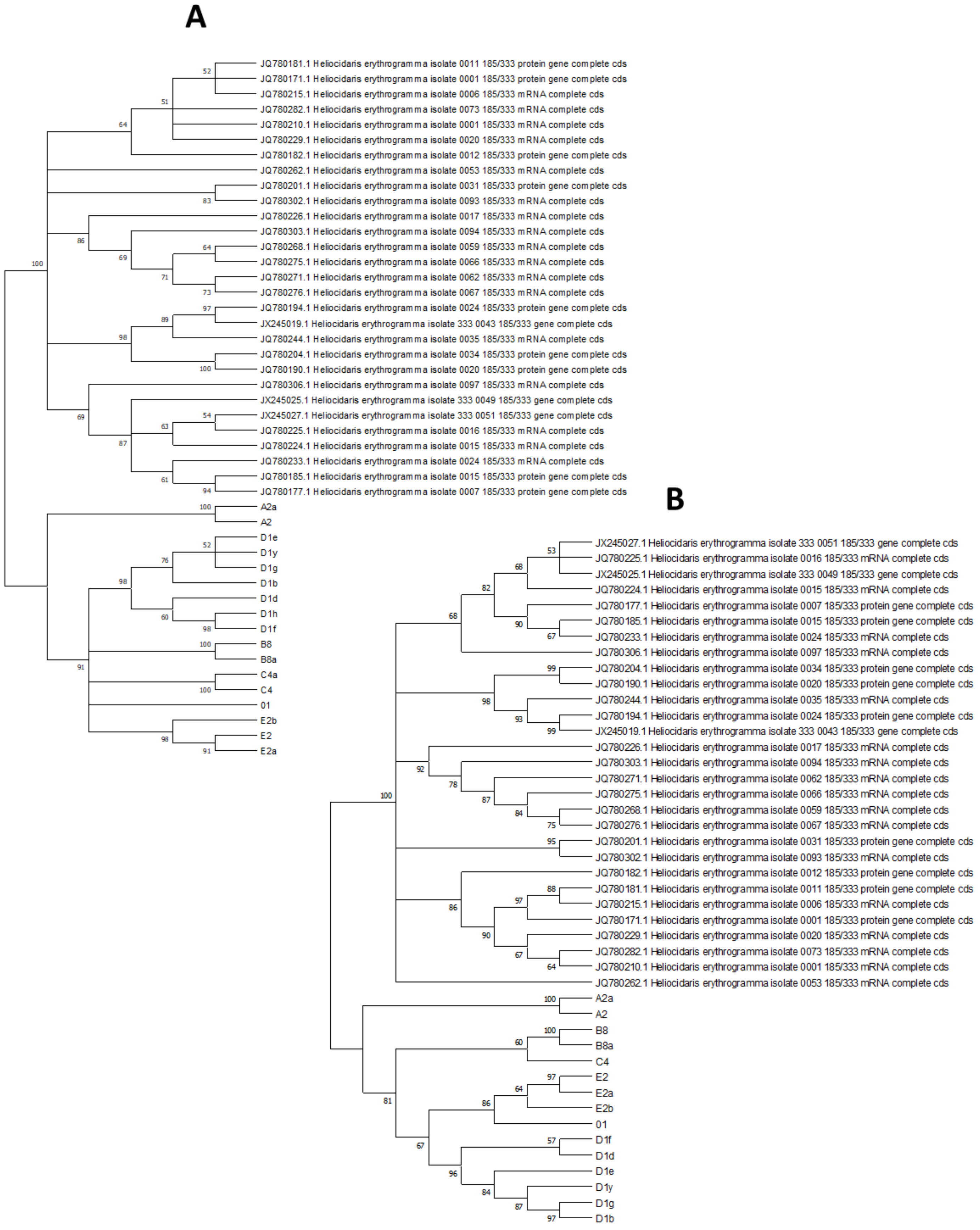
A
JQ780181.1 Heliocidaris erythrogramma isolate 0011 185/333 protein gene complete cds
JQ780171.1 Heliocidaris erythrogramma isolate 0001 185/333 protein gene complete cds
JQ780215.1 Heliocidaris erythrogramma isolate 0006 185/333 mRNA complete cds
JQ780282.1 Heliocidaris erythrogramma isolate 0073 185/333 mRNA complete cds
JQ780210.1 Heliocidaris erythrogramma isolate 0001 185/333 mRNA complete cds
JQ780229.1 Heliocidaris erythrogramma isolate 0020 185/333 mRNA complete cds
JQ780182.1 Heliocidaris erythrogramma isolate 0012 185/333 protein gene complete cds
JQ780262.1 Heliocidaris erythrogramma isolate 0053 185/333 mRNA complete cds
JQ780201.1 Heliocidaris erythrogramma isolate 0031 185/333 protein gene complete cds
JQ780302.1 Heliocidaris erythrogramma isolate 0093 185/333 mRNA complete cds
JQ780226.1 Heliocidaris erythrogramma isolate 0017 185/333 mRNA complete cds
JQ780303.1 Heliocidaris erythrogramma isolate 0094 185/333 mRNA complete cds
JQ780268.1 Heliocidaris erythrogramma isolate 0059 185/333 mRNA complete cds
JQ780275.1 Heliocidaris erythrogramma isolate 0066 185/333 mRNA complete cds
JQ780271.1 Heliocidaris erythrogramma isolate 0062 185/333 mRNA complete cds
JQ780276.1 Heliocidaris erythrogramma isolate 0067 185/333 mRNA complete cds
JQ780194.1 Heliocidaris erythrogramma isolate 0024 185/333 protein gene complete cds
JX245019.1 Heliocidaris erythrogramma isolate 333 0043 185/333 gene complete cds
JQ780244.1 Heliocidaris erythrogramma isolate 0035 185/333 mRNA complete cds
JQ780204.1 Heliocidaris erythrogramma isolate 0034 185/333 protein gene complete cds
JQ780190.1 Heliocidaris erythrogramma isolate 0020 185/333 protein gene complete cds
JQ780306.1 Heliocidaris erythrogramma isolate 0097 185/333 mRNA complete cds
JX245025.1 Heliocidaris erythrogramma isolate 333 0049 185/333 gene complete cds
JX245027.1 Heliocidaris erythrogramma isolate 333 0051 185/333 gene complete cds
JQ780225.1 Heliocidaris erythrogramma isolate 0016 185/333 mRNA complete cds
JQ780224.1 Heliocidaris erythrogramma isolate 0015 185/333 mRNA complete cds
JQ780233.1 Heliocidaris erythrogramma isolate 0024 185/333 mRNA complete cds
JQ780185.1 Heliocidaris erythrogramma isolate 0015 185/333 protein gene complete cds
JQ780177.1 Heliocidaris erythrogramma isolate 0007 185/333 protein gene complete cds
A2a
A2
D1e
D1y
D1g
D1b
D1d
D1h
D1f
B8
B8a
C4a
C4
01
E2b
E2
E2a
B
JX245027.1 Heliocidaris erythrogramma isolate 333 0051 185/333 gene complete cds
JQ780225.1 Heliocidaris erythrogramma isolate 0016 185/333 mRNA complete cds
JX245025.1 Heliocidaris erythrogramma isolate 333 0049 185/333 gene complete cds
JQ780224.1 Heliocidaris erythrogramma isolate 0015 185/333 mRNA complete cds
JQ780177.1 Heliocidaris erythrogramma isolate 0007 185/333 protein gene complete cds
JQ780185.1 Heliocidaris erythrogramma isolate 0015 185/333 protein gene complete cds
JQ780233.1 Heliocidaris erythrogramma isolate 0024 185/333 mRNA complete cds
JQ780306.1 Heliocidaris erythrogramma isolate 0097 185/333 mRNA complete cds
JQ780204.1 Heliocidaris erythrogramma isolate 0034 185/333 protein gene complete cds
JQ780190.1 Heliocidaris erythrogramma isolate 0020 185/333 protein gene complete cds
JQ780244.1 Heliocidaris erythrogramma isolate 0035 185/333 mRNA complete cds
JQ780194.1 Heliocidaris erythrogramma isolate 0024 185/333 protein gene complete cds
JX245019.1 Heliocidaris erythrogramma isolate 333 0043 185/333 gene complete cds
JQ780226.1 Heliocidaris erythrogramma isolate 0017 185/333 mRNA complete cds
JQ780303.1 Heliocidaris erythrogramma isolate 0094 185/333 mRNA complete cds
JQ780271.1 Heliocidaris erythrogramma isolate 0062 185/333 mRNA complete cds
JQ780275.1 Heliocidaris erythrogramma isolate 0066 185/333 mRNA complete cds
JQ780268.1 Heliocidaris erythrogramma isolate 0059 185/333 mRNA complete cds
JQ780276.1 Heliocidaris erythrogramma isolate 0067 185/333 mRNA complete cds
JQ780201.1 Heliocidaris erythrogramma isolate 0031 185/333 protein gene complete cds
JQ780302.1 Heliocidaris erythrogramma isolate 0093 185/333 mRNA complete cds
JQ780182.1 Heliocidaris erythrogramma isolate 0012 185/333 protein gene complete cds
JQ780181.1 Heliocidaris erythrogramma isolate 0011 185/333 protein gene complete cds
JQ780215.1 Heliocidaris erythrogramma isolate 0006 185/333 mRNA complete cds
JQ780171.1 Heliocidaris erythrogramma isolate 0001 185/333 protein gene complete cds
JQ780229.1 Heliocidaris erythrogramma isolate 0020 185/333 mRNA complete cds
JQ780282.1 Heliocidaris erythrogramma isolate 0073 185/333 mRNA complete cds
JQ780210.1 Heliocidaris erythrogramma isolate 0001 185/333 mRNA complete cds
JQ780262.1 Heliocidaris erythrogramma isolate 0053 185/333 mRNA complete cds
A2a
A2
B8
B8a
C4
E2
E2a
E2b
01
D1f
D1d
D1e
D1y
D1g
D1b

Figure S1 | Phylogenetic trees of exon 2 using Neighbor Joining (A) and Maximum Parsimony (B) show the expanded branches of exon 2 from the *HeSpTrf* genes. The phylogenetic trees of exon 2 were carried out using multiple methods in MEGA7. Bootstrap values based on 500 iterations are indicated at each node, and nodes with values below 50 were collapsed. The accession numbers for the *SpTrf* sequences used to generate these trees can be found in the materials and methods section in the main paper. The *HeTrf* sequences are reported in Roth et al. (Ref. 65 in the main paper).

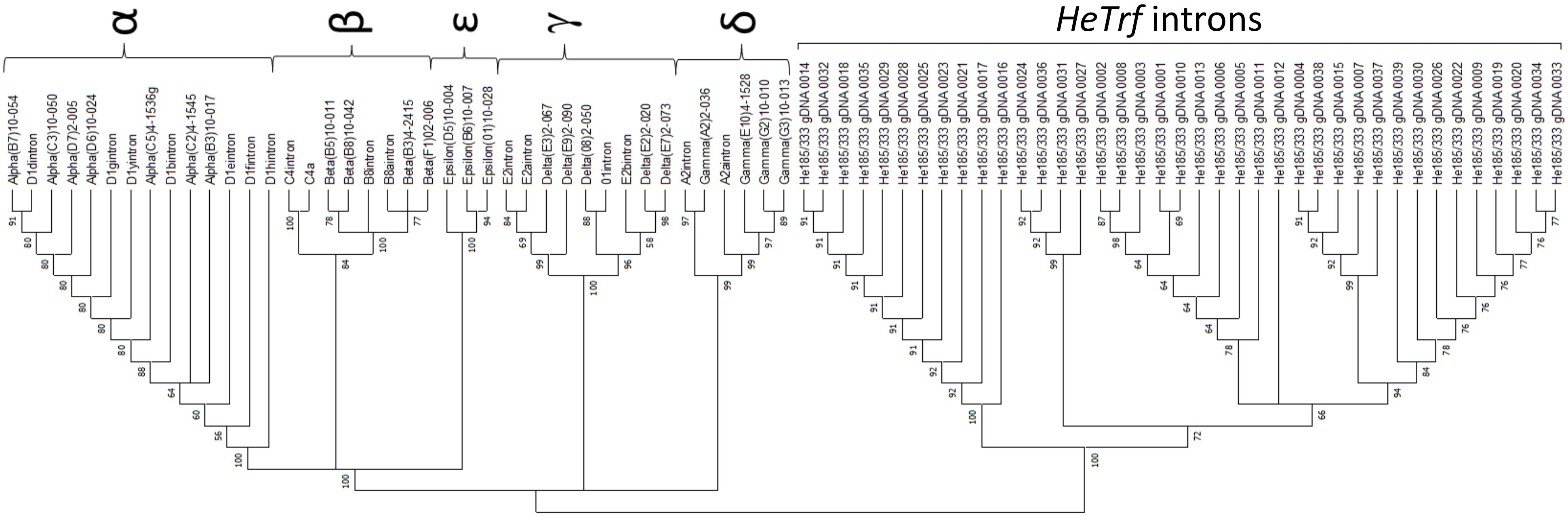

Figure S2 | The maximum likelihood phylogenetic tree of introns from the *SpTrf* genes with *HeTrf* introns as the outgroup. Phylogenetic analysis of *Trf* introns using MEGA7 shows the types of *SpTrf* introns and the details of the *HeSpTrf* intron clade indicated by the bracket. Bootstrap values from 500 iterations are shown at each node, and nodes with values below 50 were collapsed. The accession numbers for the *SpTrf* sequences used to generate these trees can be found in the materials and methods. The *HeTrf* sequences are reported in Roth et al. (Ref. 65 in the main paper).

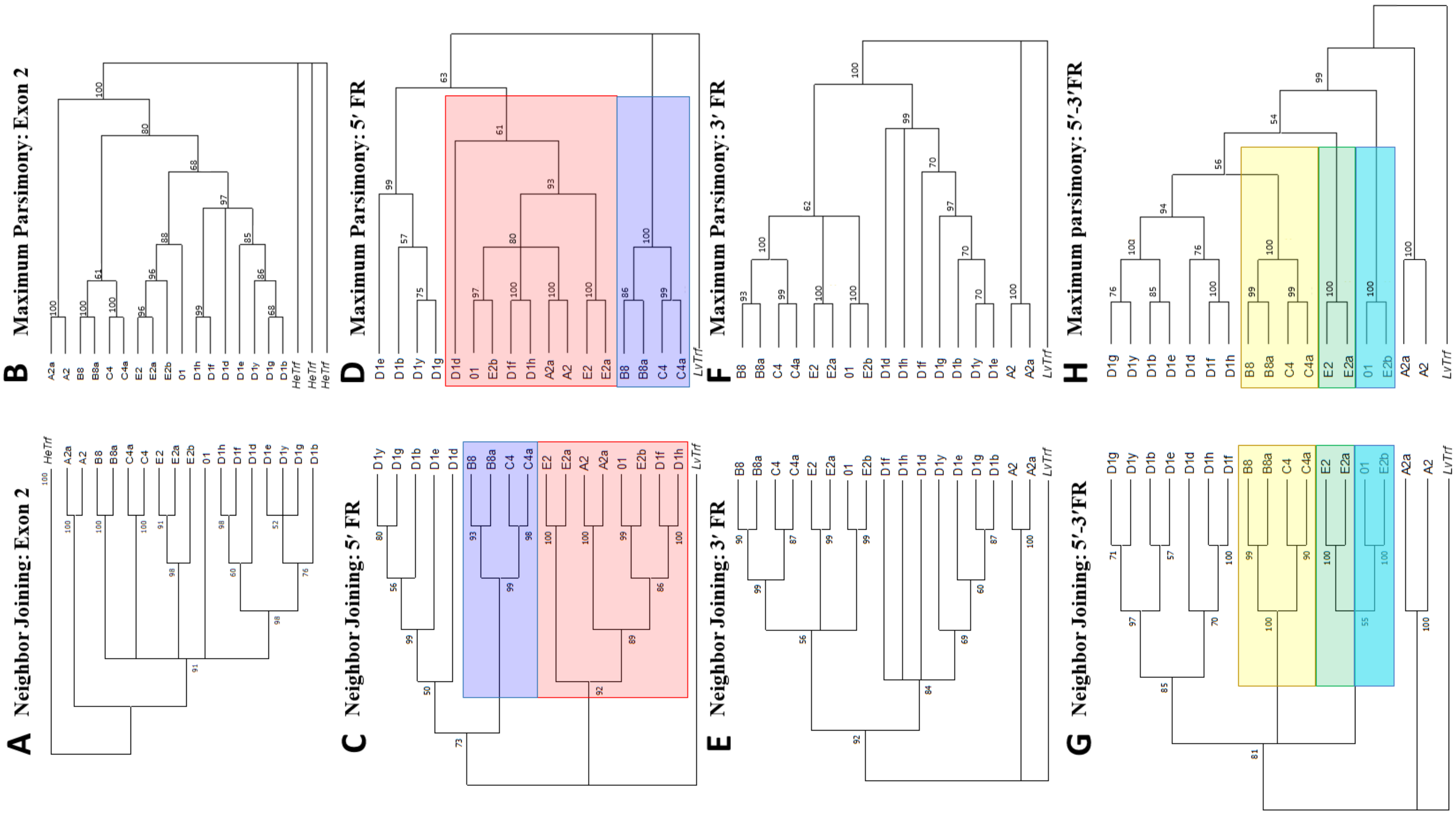

Figure S3 | Phylogenetic Trees using Neighbor Joining and Maximum Parsimony of the 5′FR, 3′FR, and Exon 2 indicate similar tree structure and gene clustering. Phylogenetic analysis of 3′FR, 5′FR, and exon 2 was done in MEGA7. Bootstrap values are based on 500 iterations and are indicated at each node, and nodes with values below 50 were collapsed. Highlighted regions of the same color indicate clades with the same genes. The accession numbers for the sequences used to generate these trees can be found in the materials and methods. The *HeTrf* sequences are reported in Roth et al. (Ref. 65 in the main paper). The *LvTrf* sequences were acquired from the *Lytechinus variegatus* genome (http://whis.caltech.edu/Echinobase/LvAbout; Davidson et al., Ref 66 in the main paper).

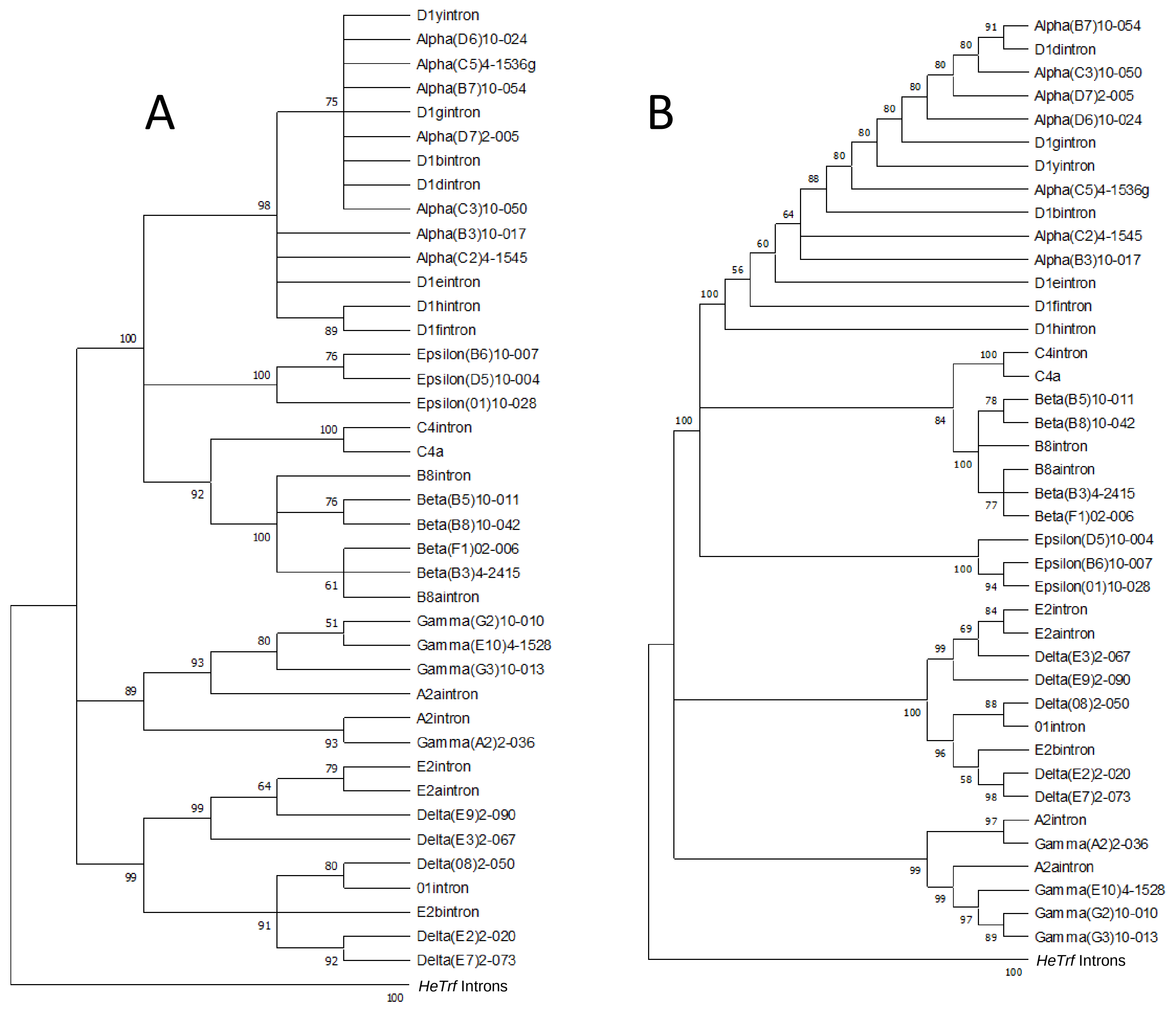
A
D1yintron
Alpha(D6)10-024
Alpha(C5)4-1536g
Alpha(B7)10-054
D1gintron
Alpha(D7)2-005
D1bintron
D1dintron
Alpha(C3)10-050
Alpha(B3)10-017
Alpha(C2)4-1545
D1eintron
D1hintron
D1fintron
Epsilon(B6)10-007
Epsilon(D5)10-004
Epsilon(01)10-028
C4intron
C4a
B8intron
Beta(B5)10-011
Beta(B8)10-042
Beta(F1)02-006
Beta(B3)4-2415
B8aintron
Gamma(G2)10-010
Gamma(E10)4-1528
Gamma(G3)10-013
A2aintron
A2intron
Gamma(A2)2-036
E2intron
E2aintron
Delta(E9)2-090
Delta(E3)2-067
Delta(08)2-050
01intron
E2bintron
Delta(E2)2-020
Delta(E7)2-073
HeTrf Introns
B
Alpha(B7)10-054
D1dintron
Alpha(C3)10-050
Alpha(D7)2-005
Alpha(D6)10-024
D1gintron
D1yintron
Alpha(C5)4-1536g
D1bintron
Alpha(C2)4-1545
Alpha(B3)10-017
D1eintron
D1fintron
D1hintron
C4intron
C4a
Beta(B5)10-011
Beta(B8)10-042
B8intron
B8aintron
Beta(B3)4-2415
Beta(F1)02-006
Epsilon(D5)10-004
Epsilon(B6)10-007
Epsilon(01)10-028
E2intron
E2aintron
Delta(E3)2-067
Delta(E9)2-090
Delta(08)2-050
01intron
E2bintron
Delta(E2)2-020
Delta(E7)2-073
A2intron
Gamma(A2)2-036
A2aintron
Gamma(E10)4-1528
Gamma(G2)10-010
Gamma(G3)10-013
HeTrf Introns

Figure S4 | Phylogenetic Trees of Introns using Neighbor Joining (A) and Maximum Parsimony (B) are used to indicate introns of similar sequence and designation. Phylogenetic analysis of introns used multiple methods in MEGA7. Bootstrap values from 500 iterations are indicated at each node, and nodes with values below 50 were collapsed. The accession numbers for the sequences used to generate these trees can be found in the materials and methods. The *HeTrf* sequences are reported in Roth et al. (Ref. 65 in the main paper).

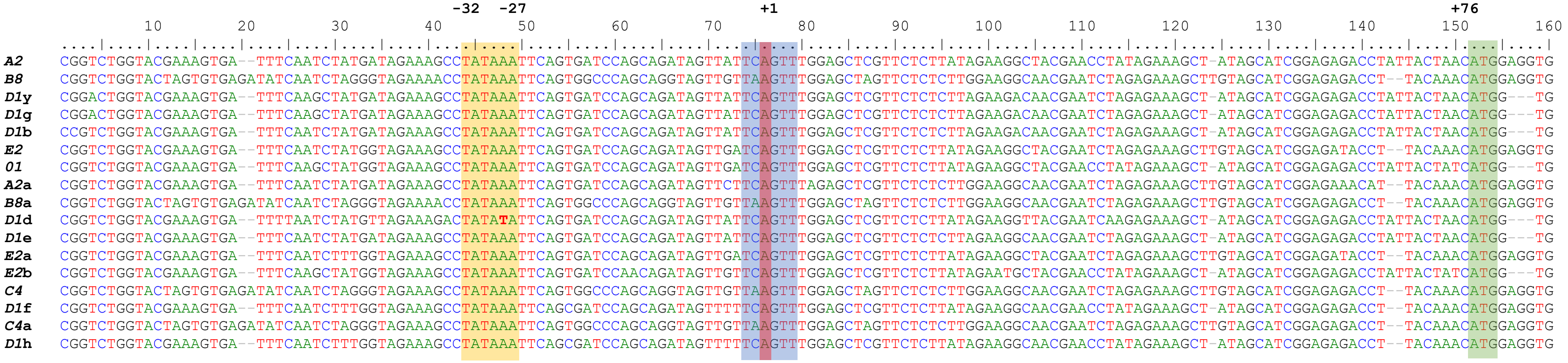

Figure S5 | Sequence elements that bind basal transcription factors in the 5′UTR for initiation of gene expression are conserved in all of the *SpTrf* genes except *D1*d. The Drosophila Inr sequence is TCA(+1)(GT)T(TC), whereas in the *SpTrf* gene sequences it is T(CA)A(+1)GTT (blue). The TATA box (yellow), the conserved +1 A within the Inr (red) and the start codon (green) are shown. Artificially inserted gaps are indicated by dashes. The alignment was done using PRANK.

```
              10        20        30        40        50        60        70        80        90       100
     ....|....|....|....|....|....|....|....|....|....|....|....|....|....|....|....|....|....|....|....|
A2   AHAQSDFNERRGKENGRERGQDRFGGRPDGMQMGGPRQDGGPMGGRRFDGPRFGAPQMGGPRQNGGPMGGRRFDGPGFGAPPMGGPRQDGGPMGGRRFDG
A2a  AHARRDFNELRGKENSRERGQDRFGGRPDGMQMGGPRQDGGPMGGRRFDGPRFGAPQMGGPRQNGGPMGGRRFDGPGFGAPPMGGPRQDGGPMGGRRFDG
B8a  AHARRDFNERRGNENGRERGQGRFGGRPGGMQMGGSRQDGGPMGGRRFDGPGFGAPHMDGRRQNGGPMGGRRFDGPGF~~~~~~~~~~~~~~~~~~~~~~
B8   AHARRDFNERRGNENGRERGQGRFGGRPGGMQMGGSRQDGGPMGGRRFDGPGFGAPHMDGRRQNGGPMGGRRFDGPGF~~~~~~~~~~~~~~~~~~~~~~
C4   AHARRDFNERRGRENGRKRGQGGFGGRPDGMQMGG~~~~~~~~~~RRFDGPGF~~~~~~~~~~~~~~~~~~~~~~~~~~~~~~~~~~~~~~~~~~~~~~~
C4a  AHARRDFNERRGRENGRKRGQGGFGGRPDGMQMGG~~~~~~~~~~RRFDGPGF~~~~~~~~~~~~~~~~~~~~~~~~~~~~~~~~~~~~~~~~~~~~~~~
D1f  AHAQRDYNERRGNENGRERGQGRFGGRPGGMQTGGPRQDGGPMGGRRFDGPDSGAPQMDGRRQ~~~~~~~~~~~~~~~~~~~~~~~~~DGGPMGGRRFDG
D1h  AHAQRDYNERRGNENGRERGQGRFGGRPGGMQTGGPRQDGGPMGGRRFDGPDSGAPQMDGRRQ~~~~~~~~~~~~~~~~~~~~~~~~~DGGPMGGRRFDG
D1d  AHAQRDYNERRGNENGRERGQGRFGGRPGGMQMGGSRQDGGPMGGKRFDGPDSGAPQMDGRRQ~~~~~~~~~~~~~~~~~~~~~~~~~DGGPMGGRRFDG
D1e  AHAQRDYNELRGNKNGRERGQGRFGGRPGGMQMGGSRQDGGPMGGRRFDGPDSGAPQMDGRRQ~~~~~~~~~~~~~~~~~~~~~~~~~DGGPMGGRRFDG
D1y  AHAQRDYNELRGNKNGRERGQGRFGGRPGGMQMGGSRQDGGPMGGRRFDGPDSGAPQMDGRRQ~~~~~~~~~~~~~~~~~~~~~~~~~DGGPMGGRRFDG
D1g  AHAQRDYNELRGNKNGRERGQGRFGGRPGGMQMSGSRQDGGPMGGRRFDGPDSGAPQMDGRRQ~~~~~~~~~~~~~~~~~~~~~~~~~DGGPMGGRRFDG
D1b  AHAQRDYNELRGNKNGRERGQGRFGGRPGGMQMGGSRQDGGPMGGRRFDGPDSGAPQMDGRRQ~~~~~~~~~~~~~~~~~~~~~~~~~DGGPMGGRRFDG
E2   AHAQRDFNERRGKENDTERGQGGFGGRPGGMQMGSPRQDGGQMGGRRFDGPESGAPQMEGRRQ~~~~~~~~~~~~~~~~~~~~~~~~~~~~~~~~~~~~~
E2a  AHAQRDFNERRGKENDTERGQGGFGGRPGGMQMGGPRQDGGPMGGRRFDGPESGAPQMEGRRQ~~~~~~~~~~~~~~~~~~~~~~~~~~~~~~~~~~~~~
E2b  AHARRDFNERRGKENGTERGQGGFGGRPGGMQTGSPRQDGG~~~~RRFDGPESGAPQMDGRRQ~~~~~~~~~~~~~~~~~~~~~~~~~~~~~~~~~~~~~
01   AHARRDFNERRGKENGRERGQGGFGGRPGGMQTGSPRQDGGPMGGMRFDGPESGAPQMDGRRQ~~~~~~~~~~~~~~~~~~~~~~~~~~~~~~~~~~~~~

             110       120       130       140       150       160       170       180       190       200
     ....|....|....|....|....|....|....|....|....|....|....|....|....|....|....|....|....|....|....|....|
A2   PGFGAPQMGGPRQNGGPMGGRRFDGPRFGGSRPDGAGGRPFFGEGGRRGDGEEETDAARQIG~~~~~PGRFDGPGHGHYGHH~~~~~~~~~~~~~~~QGA
A2a  PGFGAPQMGGPRQNGGPMGGRRFDGPGFGGSRPDGAGGRPFFGEGGRRGDGEEETDAARQIGDGLGGPGRFDGPGHGHYGHH~~~~~~~~~~~~~~~QGA
B8a  ~~~~~~~~~~~~~~~~~~~~~~~~~~~~GGSRPDGAGGRPFFGQGGRRGDGEEETDAAQQIGDGLGGRGQFDGHGRRHHGHRQGPPQDRPEEQPFGQ~~~
B8   ~~~~~~~~~~~~~~~~~~~~~~~~~~~~GGSRPDGAGGRPFFGQGGRRGDGEEETDASQQIGDGLGGRGQFDGHGRRHHGHRQGPPQDRPEEQPFGQ~~~
C4   ~~~~~~~~~~~~~~~~~~~~~~~~~~~~GGSRPDGA~GRPFFGQGGKRGDGEEETDAAQQIGDGLGGSDRFDGPRRGHHGHRQGPPQDRPEEQPFGQ~~~
C4a  ~~~~~~~~~~~~~~~~~~~~~~~~~~~~GGSRPDGA~GRPFFGQGGKRGDGEEETDAAQQIGDGLGGSDRFDGPRRGHHGHRQGPPQDRPEEQPFGQ~~~
D1f  PGFGAPEMDGRRQNGGPMGGRRFDGPGFGGSRPVGAGGRPVFGQGGRRGDGEEETDAAQQIGDGLGGPGQFDGPGRRHHGHRQGHPQDQAEEQPFGQ~~~
D1h  PGFGAPEMDGRRQNGGPMGGRRFDGPGFGGSRPVGAGGRPVFGQGGRRGDGEEETDAAQQIGDGLGGPGQFDGPGRRHHGHRQGHPQDQAEEQPFGQ~~~
D1d  PGFGAPEMDGRRQNGGPMGGRRFDGPGFGGSRPVGAGGRPFFGQGGRRGDGEEETDAAQQIGDGLGGPGQFDGPGRRHHGHRQGHPQDQAEEQPFGQ~~~
D1e  PGFGAPEMDGRRQNGGPMGGRRFDGPGFGGSRPDGAGGRPFFGQGGRRGDGEEETDAAQQIGDGLGGRGQFDGHGRRHHGHRQGPPQDRPEEQPFGQ~~~
D1y  PGFGAPEMDGRRQNGGPMGGRRFDGPGFGGSRPDGAGGRPFFGQGGRRGDGEEETDAAQQIGDGLGGPGQFDGPGRRHHGHRQGHPQDQAEEQPFGQ~~~
D1g  PGFGAPEMDGRRQNGGPMGGRRFDGPGFGGSRPDGAGGRPFFGQGGRRGDGEEETDAAQQIGDGLGGPGQFDGPGRRHHGHRQGHPQDQAEEQPFGQ~~~
D1b  PGFGAPEMDGRRQNGGPMGGRRFDGPGFGGSRPDGAGGRPFFGQGGRRGDGEEETDAAQQIGDGLGGPGQFDGPGRRHHGHRQGHPQDQAEEQPFGQ~~~
E2   ~~~~~~~~~~~~~NGGPMGGRRFDGPRFGGSRPDGAGGRPFFGQGGRRGDGEEETDAAQQIGDGLGGRGQFDGHGRGHHGHRQGPPQDRPEEQPFGQ~~~
E2a  ~~~~~~~~~~~~~NGGPMGGRRFDGPRFGGSRPDGAGGRPFFGQGGRRGDGEEETDAAQQIGDGLGGRGQFDGHGRGHHGHRQGPPQDRPEEQPFGQ~~~
E2b  ~~~~~~~~~~~~~NGGPMGGRRFDGPRFGGSRPDGAGGRPFFGQGGRRGDGEEETDAAQQIGDGLGGSGQFDGPRRGHHGHRQGPPQDRPEEQPFGQ~~~
01   ~~~~~~~~~~~~~NGGPMGGRRFDGPGFGGSRPDGAGGRPFFGQGGRRGDGEEETDAAQQIGDGLGGPGQFDGPGRRHHG~~~~~~~~~~~~~~~~~~~~
```

```
              210       220       230       240       250       260       270       280       290       300
     ....|....|....|....|....|....|....|....|....|....|....|....|....|....|....|....|....|....|....|....|
A2   GRPFFGNPPPFNPEQEPRNDSSEEDGRHHRHHDRHHAHHGHHGHHEHHHQHHNHTEGHQ~~~~~~~~~~~~~~~~~~~~~~~~~~~~~~~~~~~~~~~~~
A2a  GRPFFGNPPPFNPEQEPRNDSSEEDGRHHRHHDRHHAHHGHHGHHEHHHHHHNHSEGHQ~~~~~~~~~~~~~~~~~~~~~~~~~~~~~~~~~~~~~~~~~
B8a  ~~~~~~~~~~~~~~~~~RNERNEEDGRPHPHHH~~~GHHGHHGHHHRHH~~~NQTEGHQGHNETGD~~~~~~~~~~~~~QDQDKPNDTRPFRFNHFGRRN
B8   ~~~~~~~~~~~~~~~~~RNERNEEDGRPHPHHH~~~GHHGHQGHHHRHH~~~NQTEGHQGHNETGD~~~~~~~~~~~~~QDQDKPNDTRPFRFNHFGRRN
C4   ~~~~~~~~~~~~~~~~~RNESSEEDGRPHPHHH~~~GHHRHHRHHH~HH~~~NQTEGHQGHNDTGD~~~~~~~~~~~~~QDQDKPNDTRPFRFNHFG~~~
C4a  ~~~~~~~~~~~~~~~~~RNESSEEDGRPHPHHH~~~GHHRHHRHHH~HH~~~NQTEGHQGHNDTGD~~~~~~~~~~~~~QDQDKPNDTRPFRFNHFG~~~
D1f  ~~~~~~~~~~~~~~~~~RNESSEEDGRPHPHHH~~~~~~RHHGHHHRHH~~~NHTEGHQGHNETGD~~~~~~~~~~~~~QDQDKLHDTRPFRYNHFG~~~
D1h  ~~~~~~~~~~~~~~~~~RNESSEEDGRPHPHHH~~~~~~RHHGHHHRHH~~~NHTEGHQGHNETGD~~~~~~~~~~~~~QDQDKLHDTRPFRYNHFG~~~
D1d  ~~~~~~~~~~~~~~~~~RNKSSEEDGRPHPHHH~~~~~~RDHGHHHRHH~~~NHTEGHQGHNETGD~~~~~~~~~~~~~QDQDKLHDTRPFRYNHFG~~~
D1e  ~~~~~~~~~~~~~~~~~RNESSEEDGRPHPHHH~~~~~~RHHGHHHRHH~~~NHTEGHQGHNETGD~~~~~~~~~~~~~QDQDKLHDTRPFRYNHFG~~~
D1y  ~~~~~~~~~~~~~~~~~RNESSEEDGRPHPHHH~~~~~~RHHGHHHRHH~~~NHTEGHQGHNETGD~~~~~~~~~~~~~QDQDKLHDTRPFRYNHFG~~~
D1g  ~~~~~~~~~~~~~~~~~RNESSEEDGRPHPHHH~~~~~~RHHGRHHRHH~~~NHTEGHKGHNETGD~~~~~~~~~~~~~QDQDKLHDTRPFRYNHFG~~~
D1b  ~~~~~~~~~~~~~~~~~RNESSEEDGRPHPHHH~~~~~~RHHGRHHRHH~~~NHTEGHQGHNETGD~~~~~~~~~~~~~QDQDKLHDTRPFRYNHFG~~~
E2   ~~~~~~~~~~~~~~~~~RNESSDEDGRPHPR~~~~~~~~~HHGRHHQHHHR~NHTEGHQGHNETGD~~~~~~~~~~~~~~~~~~~~~~~~~~~~~~~~~~
E2a  ~~~~~~~~~~~~~~~~~RNESSDEDGRPHPR~~~~~~~~~HHGRHHQHHHR~NHTEGHQGHNETGD~~~~~~~~~~~~~~~~~~~~~~~~~~~~~~~~~~
E2b  ~~~~~~~~~~~~~~~~~RNESSDEDGRPHPR~~~~~~~~~HHGRHHQHHHR~NHTEGHQGHNETGD~~~~~~~~~~~~~~~~~~~~~~~~~~~~~~~~~~
O1   ~~~~~~~~~~~~~~~~~~~~~~~~~~~~~~~~~~~~~~~~~~~~~~~~~~~~~~~~~~~~~~~~~~~~~~~~~~~~~~~~~~~~~~~~~~~~~~~~~~~~

              310       320       330       340       350       360       370       380       390       400
     ....|....|....|....|....|....|....|....|....|....|....|....|....|....|....|....|....|....|....|....|
A2   ~~~~~~~~~~~~~~~~~~~~~~~~~DHDRPMFEMRPFRFNPLGRKPFGDHPFGRRNHTEGHQGHNETGDHPHRHHSKNVDGDQDTGHHGHHGHHEHHHHQ
A2a  ~~~~~~~~~~~~~~~~~~~~~~~~~DHDRPMFEMRPFRFNPLGRKPFGDHPFGRRNHTEGHQGHNETGDHPHRHHSKTVDGDQDTGHHGHHGHHEHHHHQ
B8a  HTEGHQGHNETGDHPHRHHNKTGDGDQDRPMFEMRPFWVNPFGRKPFGDRPFDRR~~~~~~~~~~~~~~~~~~~~~~~~~~~~~~~~~~~~~~~~~~~~~
B8   HTEGHQGHNETGDHPHRHHNKTGDGDQDRPMFEMRPFWVNPFGRKPFGDRPFGRR~~~~~~~~~~~~~~~~~~~~~~~~~~~~~~~~~~~~~~~~~~~~~
C4   ~~~~~~~~~~~~~~~~~~~~~~~~~~~~~~~~~~~~~~~~~~~RKPFGDRPFGRRNHTEGHQGHNETGDHPHRHHNQTGDGDQDR~~~~~~~~~~~~~~~
C4a  ~~~~~~~~~~~~~~~~~~~~~~~~~~~~~~~~~~~~~~~~~~~RKPFGDRPFGRRNHTEGHQGHNETGDHPHRHHNQTGDGDQDR~~~~~~~~~~~~~~~
D1f  ~~~~~~~~~~~~~~~~~~~~~~~~~~~~~~~~~~~~~~~~~~~RKPFGDRPFGRRNHTEGHQGHNETGDHPHRHHNKTRDGDQDR~~~~~~~~~~~~~~~
D1h  ~~~~~~~~~~~~~~~~~~~~~~~~~~~~~~~~~~~~~~~~~~~RKPFGDRPFGRRNHTEGHQGHNETGDHPHRHHNKTRDGDQDR~~~~~~~~~~~~~~~
D1d  ~~~~~~~~~~~~~~~~~~~~~~~~~~~~~~~~~~~~~~~~~~~RKPFGDRPFGRRNHTEGHQGHNETGDHPHRHHNKTRDGDQDR~~~~~~~~~~~~~~~
D1e  ~~~~~~~~~~~~~~~~~~~~~~~~~~~~~~~~~~~~~~~~~~~RKPFGDRPFGRRNHTEGHQGHNETGDHPHRHHNKTRDGDQDR~~~~~~~~~~~~~~~
D1y  ~~~~~~~~~~~~~~~~~~~~~~~~~~~~~~~~~~~~~~~~~~~RKPFGDRPFGRRNHTEGHQGHNETGDHPHRHHNKTRDGDQDR~~~~~~~~~~~~~~~
D1g  ~~~~~~~~~~~~~~~~~~~~~~~~~~~~~~~~~~~~~~~~~~~RKPFGDRPFGRRNHTEGHQGHNETGEHPHRHHNKTRDGDQDR~~~~~~~~~~~~~~~
D1b  ~~~~~~~~~~~~~~~~~~~~~~~~~~~~~~~~~~~~~~~~~~~RKPFGDRPFGRRNHTEGHQGHNETGDHPHRHHNKTRDGDQDR~~~~~~~~~~~~~~~
E2   ~~~~~~~~~~~~~HPHRHHNKTGDGDQDRPMFEMRPFRFNPFGRKPFGDRPFGRR~~~~~~~~~~~~~~~~~~~~~~~~~~~~~~~~~~~~~~~~~~~~~
E2a  ~~~~~~~~~~~~~HPHRHHNKTGDGDQDRPMFEMRPFRFNPFGRKPFGDRPFDRR~~~~~~~~~~~~~~~~~~~~~~~~~~~~~~~~~~~~~~~~~~~~~
E2b  ~~~~~~~~~~~~~HPHRHHNKTGDGDQDRPMFEMRPFRFNPFGRKPFGDRPFGRR~~~~~~~~~~~~~~~~~~~~~~~~~~~~~~~~~~~~~~~~~~~~~
O1   ~~~~~~~~~~~~~~~~~~~~~~~~~~~~~~~~~~~~~~~~~~~RKPFGDRPFGRRNHTEGHQGHNETGDHPHRHHNKTRDGDQDR~~~~~~~~~~~~~~~
```

```
             410       420       430       440       450       460       470       480       490       500
    ....|....|....|....|....|....|....|....|....|....|....|....|....|....|....|....|....|....|....|....|
A2  HDHREGHQDHDRPMFEMRPFRFNPLGRKPFGDHPFGRRNHTEGHQGHNETGDHPHRHHSKTGDGDQDRPMFETRPFWVNPFGRKPFGDRPFDRRNGTEEG
A2a HDHREGHQDHDRPMFGMRPFRFNPFGRKPFGDHPFGRRNHTEGHQGHNETGDHPHRHHSKTGDGDQDRPMFETRPFWVNPFGRKPFGDRPFDRRNGTEEG
B8a ~~~~~~~~~~~~~~~~~~~~~~~~~~~~~~~~~~~~~~~~~~~~~~~~~~~~~~~~~~~~~~~~~~~~~~~~~~~~~~~~~~~~~~~~~~~~~NGTEEG
B8  ~~~~~~~~~~~~~~~~~~~~~~~~~~~~~~~~~~~~~~~~~~~~~~~~~~~~~~~~~~~~~~~~~~~~~~~~~~~~~~~~~~~~~~~~~~~~~NRTEEG
C4  ~~~~~~~~~~~~PMFEMRPFRFNPLGRKPFGDRPFGRR~~~~~~~~~~~~~~~~~~~~~~~~~~~~~~~~~~~~~~~~~~~~~~~~~~~~~~~NGTEEG
C4a ~~~~~~~~~~~~PMFEMRPFRFNPLGRKPFGDRPFGRR~~~~~~~~~~~~~~~~~~~~~~~~~~~~~~~~~~~~~~~~~~~~~~~~~~~~~~~~NGTEEG
D1f ~~~~~~~~~~~~PMFEMRPFRFNPLGRKPFGDRPFGRR~~~~~~~~~~~~~~~~~~~~~~~~~~~~~~~~~~~~~~~~~~~~~~~~~~~~~~~~NGTEEG
D1h ~~~~~~~~~~~~PMFEMRPFRFNPLGRKPFGDRPFGRR~~~~~~~~~~~~~~~~~~~~~~~~~~~~~~~~~~~~~~~~~~~~~~~~~~~~~~~~NGTEEG
D1d ~~~~~~~~~~~~PMFESRPFRFNPFGRKPFGDRLFGRR~~~~~~~~~~~~~~~~~~~~~~~~~~~~~~~~~~~~~~~~~~~~~~~~~~~~~~~~NGTEEG
D1e ~~~~~~~~~~~~PMFEMRPFRFNPFGRKPFGGRPFDRR~~~~~~~~~~~~~~~~~~~~~~~~~~~~~~~~~~~~~~~~~~~~~~~~~~~~~~~~NGTEEG
D1y ~~~~~~~~~~~~PMFEMRPFRFNPFRRKPFGGRPFDRR~~~~~~~~~~~~~~~~~~~~~~~~~~~~~~~~~~~~~~~~~~~~~~~~~~~~~~~~NGTEEG
D1g ~~~~~~~~~~~~PMFEMRPFRFNPFGRKPFGGRPFDRR~~~~~~~~~~~~~~~~~~~~~~~~~~~~~~~~~~~~~~~~~~~~~~~~~~~~~~~~NGTEEG
D1b ~~~~~~~~~~~~PMFEMRPFRFNPFGRKPFGGRPFDRR~~~~~~~~~~~~~~~~~~~~~~~~~~~~~~~~~~~~~~~~~~~~~~~~~~~~~~~~NGTEEG
E2  ~~~~~~~~~~~~~~~~~~~~~~~~~~~~~~~~~~~~~~~~~~~~~~~~~~~~~~~~~~~~~~~~~~~~~~~~~~~~~~~~~~~~~~~~~~~~~NGTEEG
E2a ~~~~~~~~~~~~~~~~~~~~~~~~~~~~~~~~~~~~~~~~~~~~~~~~~~~~~~~~~~~~~~~~~~~~~~~~~~~~~~~~~~~~~~~~~~~~~NGTEEG
E2b ~~~~~~~~~~~~~~~~~~~~~~~~~~~~~~~~~~~~~~~~~~~~~~~~~~~~~~~~~~~~~~~~~~~~~~~~~~~~~~~~~~~~~~~~~~~~~NGTEEG
01  ~~~~~~~~~~~~PMFEMRPFRFNPFGRKPFGDRPFGRR~~~~~~~~~~~~~~~~~~~~~~~~~~~~~~~~~~~~~~~~~~~~~~~~~~~~~~~~NGTEEG

             510       520       530       540       550       560
    ....|....|....|....|....|....|....|....|....|....|....|....|....|...
A2  SPRRDGHPHPHGNRGRWGENESEEKEHPTTESVTTSSPLKVIEIAINEVDTNVVAEV*
A2a SPRRDGHPHPHGNRGRWGENESEEKEHPTTESVTTSSPLKVIEIAINEVDTNVVAEV*
B8a SPRRDGHRHPYGNRGRWGENESEEKEHPTTESITTSSPPEVVEIAVNEEDVNVVAEVYNLYKKRLLV*
B8  SPRRDGHRHPYGNRGRWGENESEEKEHPTTESVTTSSPPEVVEIAVNEEDVNVVAEVYNLYKKRLLV*
C4  SLRRDGHRRPYGNRGRWGENESEEKEHPTTESVTTSSPPDVVEIAVNEEDVNVVAEV*
C4a SLRRDGHRRPYGNRGRWGENESEEKEHPTTESVTTSSPPDVVEIAVNEEDVNVVAEV*
D1f SPRRDGQRRPYGNRGR*
D1h SPRRDGQRRPYGNRGR*
D1d SSRRDGHRRPYGNRGRWGENESEEKEHPTTESVTTSSPPEVV~~AINEEDINVVAEV*
D1e SPRRDGHRRPYGNRGRWGENESEEKEHPTTESVTTSSPLEVV~~AINEEDINVVAEV*
D1y SPRRDGHRRPYGNRGRWGENESEEKEHPTTESVTTSSPPEVV~~AINEEDINVVAEV*
D1g SPRRDGHRRPYGNRGRWGESESEEKEHPTTESVTTSSPPEVV~~AINEEDINVVAEV*
D1b SPRRDGHRRPYGNRGRWGENESEEKEHPTTESVTTSSPPEVV~~AINEEDINVVAEV*
E2  SPRRDGQRRPYGNRGRWGENESEEKEHPTTESVTTSSPP*
E2a SPRRDGQRRPYGNRGRWGENESEEKEHPTTESVTTSSPP*
E2b SPRRDGQRRPYGNRGRWGENESEEKEHPTTESVTTSSPP*
01  SPRRDGQRRPHGNRGRWGENESEEKEHPTTESVTTSSPPDVVEIAIND~~~~~VAEV*
```

Figure S6 | The repeat based amino acid alignment of the mature SpTrf proteins indicates that exon 2 is in frame for all genes. The amino acid sequence deduced from the genes in the clusters were aligned by hand in BioEdit (ver 7.2.5). The protein names are indicated to the left, and the ruler above each alignment indicates the amino acid position. The ~ indicates the insertion of artificial gaps in the alignment where the sequences do not match. The * indicates a stop. The blue highlighted region defines element 15 of the alignment, which is used to name the genes/proteins according to Terwilliger et al. (Ref. 12 in the main paper).

```
                10
     ....|....|....|...
A2   MEVKVTLIVAIVAALAIS
A2a  ....A........V....
B8   ....A.............
B8a  ....A.............
D1y  .~................
D1g  .~................
D1b  .~.............V..
D1f  ....A.............
D1h  ....A.............
D1d  .~................
D1e  .~................
C4   ..................
C4a  ..................
E2   ..................
E2a  ..................
E2b  .~................
01   .~............F...
```

Figure S7 | The alignment of the deduced amino acids from exon 1 for each SpTrf protein shows slight variations in the leader. The gene names are listed to the left and the numbers above indicate the amino acid position in the deduced proteins. The *A2* sequence was used for comparison to the other proteins and the dots indicate matching amino acids. The ~ indicates the insertion of artificial gaps in the alignment where the sequences do not match. The alignment was done manually in BioEdit (ver 7.2.5).

```
                                10        20        30        40        50        60        70        80        90       100
                        ....|....|....|....|....|....|....|....|....|....|....|....|....|....|....|....|....|....|....|....|
Alpha (B3) 10-017       GTAAGAAATCAAATTATTACTCGGTATTACTTGATAAGTGCTAAATACAGAGCCAACGAATAG~CTCGCAGAAGTATAT~~GATTATTT~CATATTTTAT
Alpha (B7) 10-054       GTAAGAAATCAAATTATTACTCGGTATTACTTGATAAGTGCTAAATATAGAGCCAACGAATAG~CTCACAGAAGTATATATGATTATTT~CATATTTTAT
Alpha (C5) 4-1536g      GTAAGAAATCAAATTATTACTCGGTATTACTTGATAAGTGCTAAATATAGAGCCAACGAATAG~CTCACAGAAGTATATATGATTATTT~CATATTTTAT
Alpha (C3) 10-050       GTAAGAAATCAAATTATTACTCGGTATTACTTGATAAGTGCTAAATATAGAGCCAACGAATAG~CTCACAGAAGTATATATGATTATTT~CATATTTTAT
Alpha (D6) 10-024       GTAAGAAATCAAATTATTACTCGGTATTACTTGATAAGTGCTAAATATAGAGCCAACGAATAG~CTCACAGAAGTATATATGATTATTT~CATATTTTAT
Alpha (C2) 4-1545       GTAAGAAATCAAATTATTACTCGGTATTACTTGATAAGTGCTAAATATAGAGCCAACGAATAG~CTCGCAGAAGTATATATGATTATTT~CATATTTTAT
Alpha (D7) 2-005        GTAAGAAATCAAATTATTACTCGGTATTACTTGATAAGTGCTAAATATAGAGCCAACGAATAG~CTCACAGAAGTATATATGATTATTT~CATATTTTAT
D1f intron              GTAAGAAATCAAATTATTACTCGGTATTCCTTGATAAGTGCTAAATATAGAGCCAACGAATAG~CTCACAGAAGTATATATGATTATTT~CATATTTTAT
D1h intron              GTAAGAAATCAAATTATTACTCGGTATTCCTTGATAAGTGCTAAATATAGAGCCAACGAATAG~CTCACAGAAGTATATATGATTATTT~CATATTTTAT
D1e intron              GTAAGAAATCAAATTATTACTCGGTATTACTTGATAAGTGCTAAATATAGAGCCAACGAATAG~CTCACAGAAGTATATATGATTATTT~CATATTTTAT
D1d intron              GTAAGAAATCAAATTATTACTCGGTATTACTTGATAAGTGCTAAATATAGAGCCAACGAATAG~CTCACAGAAGTATATATGATTATTT~CATATTTTAT
D1y intron              GTAAGAAATCAAATTATTACTCGGTATTACTTGATAAGTGCTAAATATAGAGCCAACGAATAG~CTCACAGAAGTATATATGATTATTT~CATATTTTAT
D1g intron              GTAAGAAATCAAATTATTACTCGGTATTACTTGATAAGTGCTAAATATAGAGCCAACGAATAG~CTCACAGAAGTATATATGATTATTT~CATATTTTAT
D1b intron              GTAAGAAATCAAATTATTACTCGGTATTACTTGATAAGTGCTAAATATAGAGCCAACGAATAG~CTCACAGAAGTATATATGATTATTT~CATATTTTAT
C4 intron               GTAAGAAATCAAATTATTACTCGGTATTACTTGATAAGTGCTAAATATAAAGCCAACGAAGGG~TTCACAGAAGTAAATATGATTATTT~CATAGTTTGT
B8 intron               GTAAGAAATCAAATTATTACTCGGTATTACTTGATAAGTGCTAAATATAAAGCCAACGAAGGG~CTCACAGAAGTAAATATGATTATTT~CATAGTTTGT
B8a intron              GTAAGAAATCAAATT~TTACTCGGTATTACTTCATAAGTGCTAAATATAAAGCCAATGAAGGG~CTCACAGAAGTAAATATGATTATTT~CATAGTTTGT
Beta (B5) 10-011        GTAAGAAATCAAATTATTACTCGGTATTACTTGATAAGTGCTAAATATAAAGCCAACGAAGGG~CTCACAGAAGTAAATATGATTATTT~CATAGTTTGT
Beta (B3) 4-2415        GTAAGAAATCAAATTATTAGTCGGTATTCCTTCATAAGTGCTAAATATAAAGCCAACGAAGGG~CTCACAGAAGTAAATATGATTATTT~CATAGTTTGT
Beta (B8) 10-042        GTAAGAAATCAAATTATTACTCGGTATTACTTGATAAGTGCTAAATATAAAGCCAACGAAGGG~CTCACAGAAGTAAATATGATTATTT~CATAGTTTGT
Beta (F1) 02-006        GTAAGAAATCAAATTATTACTCGGTATTACTTCATGAGTGCTAAATATAAAGCCAACGAAGGG~CTCACAGAAGTAAATATGATTATTT~CATAGTTTGT
Epsilon (B6) 10-007     GTAAGAAATCAAATTATTACTCGGTATTCCTTGATAAGTGCTAATTATAAAGCCAACGAAAGGGCGCACAGGAGTTT~~~~GATTATTT~CATTTTTTAT
Epsilon (D5) 10-004     GTAAGAAATCAAATTATTACTCGGTATTCCTTGATAAGTGCTAATTATAAAGCCAACGAAAGGGCGCACAGGAGTTT~~~~GATTATTT~CATTTTTTAT
Epsilon (01) 10-028     GTAAGAAATCAAATTATTACTCGGTATTCCTTGATAAGTGCTAATTATAAAGCCAACGAAA~~~~~~ACAGGAGTTT~~~~GATTATTT~CATTTTTTAT
A2 intron               GTAAGAAATCAAATTATTACTTGGTATTACTTGATAAGTGGCAAATATTAAGCCAACAAAAGG~CTCACAGGAGTATAT~~TATTATTT~CATTTATCAT
A2a intron              GTAAGAAATCAAATTATTACTTGGTATTACTTGATAAGTGGCAAATATTAAGCCAAGAAAGGG~CTCACAGGAGTATATT~~ATTATTT~AATTTATCAT
Gamma (A2) 2-036        GTAAGAAATCAAATTATTACTTGGTATTACTTGATAAGTGGCAAATATTAAGCCAACAAAAGG~CTCACAGGAGTATAT~T~ATTATTT~CATTTATCAT
Gamma (G2) 10-010       GTAAGAAATCAAATTATTACTTGGTATTACTTGATAAGTGGCAAATATTAAGCCAACAAAAGG~CTCACAGGAGTATAT~T~ATTATTT~CATTTATCAT
Gamma (G3) 10-013       GTAAGAAATCAAATTATTACTTGGTATTACTTGATAAGTGGCAAATATTAAGCCAACAAAAGG~CTCACAGGAGTATAT~T~ATTATTT~CATTTATCAT
Gamma (E10) 4-1528      GTAAGAAATCAAATTATTACTTGGTATTACTTGATAAGTGGCAAATATTAAGCCAACAAAAGG~CTCACAGGAGTATAT~T~ATTATTT~CATTTATCAT
Delta (E2) 2-020        GTAAGAAATCAAATTATTACTCGGTATTCCTTCATAAGTGGTA~~TATTGAGCCAACAAAATTACTCACATAAGTATAT~TG~TTATTTTGATTTATTAT
Delta (E3) 2-067        GTAAGAAATCAAATTATTACTCGGTATTCCTTCATAAGTGTTAAATATTAAGCCAACAAATGA~~~~~~~~~~~~~~~~~~~~~~~~~~~~~~~~~~~~~
Delta (E7) 2-073        GTAAGAAATCAAATTATTACTCGGTATTCCTTCATAAGTGGTA~~TATTGAGCCAACAAAATTACTCACATAAGTATAT~TG~TTATTTTGATTTATTAT
Delta (E9) 2-090        GTAAGAAATCAAATTATTACTCGGTATTCCTTCATAAGTGTTAAATATTAAGCCAACAAATGA~CTCACAGTAGTATAT~T~ATTATTTTGATTTATTAT
Delta (08) 2-050        GTAAGAAATCAAATTATTACTCGGTATTCCTTCATAAGTGGTA~~TATTGAGCCAACAAAATTACTCACATAAGTATAT~TG~TTATTTTGATTTATTAT
E2 intron               GTAAGAAATCAAATTATTACTCGGTATTCCTTCATAAGTGTTAAATATTAAGCCAACAAATGA~CTCACAGTAGCATATT~~ATTATTTTGATTTATTAT
E2a intron              GTAAGAAATCAAATTATTACTCGGTATTCCTTCATAAGTGTTAAATATTAAGCCAACAAATGA~CTCACAGTAGTATATT~~ATTATTTTGATTTATTAT
E2b intron              GTAAGAAATCAAATTATTACTCGGTATTCCTTCATAAGTGGTA~~TATTGAGCCAACAAAATTACTCACATAAGTATATT~G~TTATTTTGATTTATTAT
01 intron               GTAAGAAATCAAATTATTACTCGGTATTCTTTCATAAGTGG~~~~TATATTG~~~~~~~~~~~~~~~~~~~~~~~~~~~~~~~~~~~~~~~~~~~~~~~~
```

```
                            110       120       130       140       150       160       170       180       190       200
                     ....|....|....|....|....|....|....|....|....|....|....|....|....|....|....|....|....|....|....|....|
Alpha (B3) 10-017    AATATGCATTTCTAAATTGTTCGTTACACAATATAA~~~~~~~~~~~~~~~~~~~~~~~~~~~~~~~~~~~~~~~~~~~~TTTATATTATTTCTTAAGCC
Alpha (B7) 10-054    AATATGCATTTCTAAATTGTTCGTTACAAAATATAA~~~~~~~~~~~~~~~~~~~~~~~~~~~~~~~~~~~~~~~~~~~~TTTATATTATTTCTTAAGCC
Alpha (C5) 4-1536g   AATATGCATTTCTAAATTGTTCGTTACAAAATATAA~~~~~~~~~~~~~~~~~~~~~~~~~~~~~~~~~~~~~~~~~~~~TTTATATTATTTCTTAAGCC
Alpha (C3) 10-050    AATATGCATTTCTAAATTGTTCGTTACAAAATATAA~~~~~~~~~~~~~~~~~~~~~~~~~~~~~~~~~~~~~~~~~~~~TTTATATTATTTCTTAAGCC
Alpha (D6) 10-024    AATATGCATTTCTAAATTGTTCGTTACAAAATATAA~~~~~~~~~~~~~~~~~~~~~~~~~~~~~~~~~~~~~~~~~~~~TTTATATTATTTCTTAAGCC
Alpha (C2) 4-1545    AATATGCATTTCTAAATTGTTCGTTACACAATATAA~~~~~~~~~~~~~~~~~~~~~~~~~~~~~~~~~~~~~~~~~~~~TTTATATTATTTCTTAAGCC
Alpha (D7) 2-005     AATATGCATTTCTAAATTGTTCGTTACAAAATATAA~~~~~~~~~~~~~~~~~~~~~~~~~~~~~~~~~~~~~~~~~~~~TTTATATTATTTCTTAAGCC
D1f intron           AATATGCATTTCTAAATTGTTCGTTACACAATATAA~~~~~~~~~~~~~~~~~~~~~~~~~~~~~~~~~~~~~~~~~~~~TTTATATTATTTCTTAAGCC
D1h intron           AATATGCATTTCTAAATTGTTCGTTACACAATATAA~~~~~~~~~~~~~~~~~~~~~~~~~~~~~~~~~~~~~~~~~~~~TTTATATTATTTCTTAAGCC
D1e intron           AATATGCATTTCTAAATTGTTCGTTACACAAAATAA~~~~~~~~~~~~~~~~~~~~~~~~~~~~~~~~~~~~~~~~~~~~TTTATATTATTTCTTAAGCC
D1d intron           AATATGCATTTCTAAATTGTTCGTTACAAAATATAA~~~~~~~~~~~~~~~~~~~~~~~~~~~~~~~~~~~~~~~~~~~~TTTATATTATTTCTTAAGCC
D1y intron           AATATGCATTTCTAAATTGTTCGTTACAAAATATAA~~~~~~~~~~~~~~~~~~~~~~~~~~~~~~~~~~~~~~~~~~~~TTTATATTATTTCTTAAGCC
D1g intron           AATATGCATTTCTAAATTGTTCGTTACAAAATATAA~~~~~~~~~~~~~~~~~~~~~~~~~~~~~~~~~~~~~~~~~~~~TTTATATTATTTCTTAAGCC
D1b intron           AATATGCATTTCTAAATTGTTCGTTACAAAATATAA~~~~~~~~~~~~~~~~~~~~~~~~~~~~~~~~~~~~~~~~~~~~TTTATATTATTTCTTAAGCC
C4 intron            AATATGCATTTTTAAATTGTTCGTTACAAATTATAA~~~~~~~~~~~~~~~~~~~~~~~~~~~~~~~~~~~~~~~~~~~~TTAATTTTATTTCTTAAACC
B8 intron            AATATGCATTTTTAAATTGTTCGTTACACAATATAA~~~~~~~~~~~~~~~~~~~~~~~~~~~~~~~~~~~~~~~~~~~~TTAATTGTATTTATTAAGCC
B8a intron           AATATGCATTTTTAAATTGTTCGTTACACAATATAA~~~~~~~~~~~~~~~~~~~~~~~~~~~~~~~~~~~~~~~~~~~~TTAATTGTATTTATTAAGCC
Beta (B5) 10-011     AATATGCATTTTTAAATTGTTCGTTACACAATATAA~~~~~~~~~~~~~~~~~~~~~~~~~~~~~~~~~~~~~~~~~~~~TTAATTGTATTTATTAAGCC
Beta (B3) 4-2415     AATATGCATTTTTAAATTGTTCGTTACACAATATAA~~~~~~~~~~~~~~~~~~~~~~~~~~~~~~~~~~~~~~~~~~~~TTAATTGTATTTATTAAGCC
Beta (B8) 10-042     AATATGCATTTTTAAATTGTTCGTTACACAATATAA~~~~~~~~~~~~~~~~~~~~~~~~~~~~~~~~~~~~~~~~~~~~TTAATTGTATTTATTAAGCC
Beta (F1) 02-006     AATATGCATTTTTAAATTGTTCGTTACACAATATAA~~~~~~~~~~~~~~~~~~~~~~~~~~~~~~~~~~~~~~~~~~~~TTAATTGTATTTATTAAGCC
Epsilon (B6) 10-007  AATATGCATTTCTGAATTGTTTGTTACACAATATAA~~~~~~~~~~~~~~~~~~~~~~~~~~~~~~~~~~~~~~~~~~~~TTAACTTTATTTCTTAAACC
Epsilon (D5) 10-004  AATATGCATTTCTGAATTGTTTGTTACACAATATAA~~~~~~~~~~~~~~~~~~~~~~~~~~~~~~~~~~~~~~~~~~~~TTAACTTTATTTCTTAAACC
Epsilon (01) 10-028  AATATGCATTTCTGAATTGTTTGTTACACAATATAA~~~~~~~~~~~~~~~~~~~~~~~~~~~~~~~~~~~~~~~~~~~~TTAACTTTATTTCTTAAACC
A2 intron            AATATGTGTTTCTTACCTGTTTGTTACACAATACAC~AAAATATTTCTTCTGT~GGGCTT~~~~~~~~~~~~~~~~~~~~~~~~~~~~~~~~~~~~~~~~
A2a intron           AATATGTGTTTCTTACCTGTTTGTTACACAATACAA~AAAATATTTCTTCTGT~GGGCTT~~~~~~~~~~~~~~~~~~~~~~~~~~~~~~~~~~~~~~~~
Gamma (A2) 2-036     AATATGTGTTTCTTACCTGTTTGTTACACAATACAC~AAAATATTTCTTCTGT~GGGCTT~~~~~~~~~~~~~~~~~~~~~~~~~~~~~~~~~~~~~~~~
Gamma (G2) 10-010    AATATGTGTTTCTTACCTGTTTGTTACACAATACAC~AAAATATTTCTTCTGT~GGGCTT~~~~~~~~~~~~~~~~~~~~~~~~~~~~~~~~~~~~~~~~
Gamma (G3) 10-013    AATATGTGTTTCTTACCTGTTTGTTACACAATACAC~AAAATATTTCTTCTGT~GGGCTT~~~~~~~~~~~~~~~~~~~~~~~~~~~~~~~~~~~~~~~~
Gamma (E10) 4-1528   AATATGTGTTTCTTACCTGTTTGTTACACAATACAC~AAAATATTTCTTCTGT~GGGCTT~~~~~~~~~~~~~~~~~~~~~~~~~~~~~~~~~~~~~~~~
Delta (E2) 2-020     AATATGTGTTTCTAACTTGTTTGTGACACAATAAAAAACAACATTTATTCTG~CGGCCTGCAGCATTCTTGTTTTTTTT~~~~~~~~~~~~~~~~~~~~~
Delta (E3) 2-067     ~~~~~~~~~~~~~~~~~~~~~CGTAACACAATAAAAA~TAACATTTATTCTT~CGGCCTGCAGCATTCTGTTTTTTTTT~~~~~~~~~~~~~~~~~~~~~
Delta (E7) 2-073     AATATGTGTTTCTAACTTGTTTGTGACACAATAAAAAACAACATTTATTCTG~CGGCCTGCAGCATTCTTGTTTTTTTT~~~~~~~~~~~~~~~~~~~~~
Delta (E9) 2-090     AAAAGGTGTTTCTAACTTGTTTGTAACACAATAAAAA~TAACATTTATTCTT~CGGCCTGCAGCATTCTGTTTTTTTT~~~~~~~~~~~~~~~~~~~~~~
Delta (08) 2-050     AATATGTGTTTCTAACTTGTTTGTGACACAATAAAAAATAACATTTATTCTG~CGGCCTGCAGCATTCTGTTTTTTCTT~~~~~~~~~~~~~~~~~~~~~
E2 intron            AAAAGGTGTTTCTAACTTGTTTGTAACACAATAAAAA~TAACATTTATTCTT~CGGCCTGCAGCATTCTGTTTTTTTTTT~~~~~~~~~~~~~~~~~~~~
E2a intron           AAAAGGTGTTTCTAACTTGTTTGTAACACAATAAAAA~TAACATTTATTCTT~CGGCCTGCAGCATTCTGTTTTTTTTT~~~~~~~~~~~~~~~~~~~~~
E2b intron           AATATGTGTTTCTAACTTGTTTGTGACACAATAAAAAATAACATTTATTCTG~CGGCCTGCAGCATTCTGGTTTTTTT~~~~~~~~~~~~~~~~~~~~~~
01 intron            ~~~~~~~~~~~~~AACTTGTTTGTGACACAATAAAAAATAACATTTATTCTG~CGGCCTGCAGCATTCTGTTTTTTTT~~~~~~~~~~~~~~~~~~~~~~
```

```
                              210       220       230       240       250       260       270       280       290       300
                     ....|....|....|....|....|....|....|....|....|....|....|....|....|....|....|....|....|....|....|....|
Alpha (B3) 10-017    TACACCAATCTGTTGGT~CGAATGGCAAAAGATAAGAATTCTCTTTATGTTCAACCTGGTATTCAAGTTCAATTCAGTCAGAATTAGGCGTTTTGAATAT
Alpha (B7) 10-054    TACACCAATCCGTTGGG~CGAGTGGCAAAAGATAAGAATTATCTTTATGTTCAATCTGGTATTCAAGTTCAATTCAGTCAGAATTAGGCGTTTTGAATAT
Alpha (C5) 4-1536g   TACACCAATCCGTTGGG~CGAGTGGCAAAAGATAAGAATTATCTTTATGTTCAATCTGGTATTCAAGTTCAATTCAGTCAGAATTAGGCGTTTTGAATAT
Alpha (C3) 10-050    TACACCAATCCGTTGGG~CGAGTGGCAAAAGATAAGAATTATCTTTATGTTCAATCTGGTATTCAAGTTCAATTCAGTCAGAATTAGGCGTTTTGAATAT
Alpha (D6) 10-024    TACACCAATCCGTTGGG~CGAGTGGCAAAAGATAAGAATTATCTTTATGTTCAATCTGGTATTCAAGTTCAATTCAGTCAGAATTAGGCGTTTTGAATAT
Alpha (C2) 4-1545    TACACCAATCCGTTGGT~CGAATGGCAAAAGATAAGAATTATCTTTATGTTCAATCTGGTATTCAAGTTCAATTCAATCAGAATTAGGCGTTTTGAATAT
Alpha (D7) 2-005     TACACCAATCCGTTGGG~CGAGTGGCAAAAGATAAGAATTATCTTTATGTTCAATCTGGTATTCAAGTTCAATTCAGTCAGAATTAGGCGTTCTGAATAT
D1f intron           TACACCAATCCGTTGGT~CGAATGGCAAAAGATAAGAATTATCTTTATGTTCAATCTGGTATTCAAGTTCAATTCAGTCAGAATTAGGCGTTTTGAATAT
D1h intron           TACACCAATCCGTTGGT~CGAATGGCAAAAGATAAGAATTATCTTTATGTTCAATCTGGTATTCAAGTTCAATTCAGTCAGAATTAGGCGTTTTGAATAT
D1e intron           TACACCAATCCGTTGGT~CGAATGGCAAAAGATAAGAATTATCTTTATGTTCAATCTGGTATTCAAGTTCAATTCAGTCAGAATTAGGCGTTTTGAATAT
D1d intron           TACACCAATCCGTTGGG~CGAGTGGCAAAAGATAAGAATTATCTTTATATTCAATCTGGTTTTCAAGTTCAATTCAGTCAGAATTAGGCGTTTTGAATAT
D1y intron           TACACCAATCCGTTGGG~CGAGTGGCAAAAGATAAGAATTATCTTTATGTTCAATCTGGTATTCAAGTTCAATTCAGTCAGAATTAGGCGTTTTGAATAT
D1g intron           TACACCAATCCGTTGGG~CGAGTGGCAAAAGATAAGAATTATCTTTATGTTCAATCTGGTATTCAAGTTCAATTCAGTCAGAATTAGGCGTTTTGAATAT
D1b intron           TACACCAATCCGTTAGG~CGAGTGGCAAAAGATAAGAATTATCTTTATGTTCAATCTGGTATTCAAGTTCAATTCAGTCAGAATTAGGCGTTTTGAATAT
C4 intron            TACACCAATCCGTTGGT~CGAATGACAAAAGATGTGTATTATGTTTACGTTCAATCTGGTATTCAAATTTAATTCAGTCAGAATAGGGCATTTTGAAGGT
B8 intron            TACACCAATCCGTTTTC~TGAATGACAAAGGATGTGTATTATGTTTACGTTCAATCTGGTAATCAAATTTAATTCAGTCAGAATTAGGCGTTTTGAATAT
B8a intron           TACACCAATCCGTTTTC~TGAATGACAAAGGATGTGTATTATGTTTACGTTCAATCTGGTAATCAAATTTAATTCAGTCAGAATTAGGCGTTTTGAATAT
Beta (B5) 10-011     TACACCAATCCGTTTTC~TGAATGACAAAGGATGTGTATTATGTTTACGTTCAATCTGGTAATCAAATTTAATTCAGTCAGAATTAGGCGTTTTGAATAT
Beta (B3) 4-2415     TACACCAATCCGTTTTC~TGAATGACAAAGGATGTGTATTATGTTTACGTTCAATCTGGTAATCAAATTTAATTCAGTCAGAATTAGGCGTTTTGAATAT
Beta (B8) 10-042     TACACCAATCCGTTTTC~TGAATGACAAAGGATGTGTATTATGTTTACGTTCAATCTGGTAATCAAATTTAATTCAGTCAGAATTAGGCGTTTTGAATAT
Beta (F1) 02-006     TACACCAATCCGTTTTC~TGAATGACAAAGGATGTGTATTATGTTTACGTTCAATCTGGTAATCAAATTTAATTCAGTCAGAATTAGGCGTTTTGAATAT
Epsilon (B6) 10-007  TACACCAATCCGTTGGT~CGAATGACAAAAGATGTGTATTATCTTTATGTTCAATCTGGTATTCAAATTCAATTCAGTCAGAATTAGGCGTTTTGAATAT
Epsilon (D5) 10-004  TACACCAATCCGTTGGT~CGAATGACAAAAGATGTGTATTATCTTTATGTTCAATCTGGTATTCAAATTCAATTCAGTCAGAATTAGGCGTTTTGAATAT
Epsilon (01) 10-028  TACACCAATCCGTTGGT~CGAATGACAAAAGATGTGTATTATCTTTATGTTCAATCTGGTATTCAAATTCAATTCAGTCAGAATTAGGCGTTTTGAATAT
A2 intron            ~~CAGCATTCCGTTTATTCGAATGAAAAACACGTTTTA~~~TGTTTCTTTACCATCTGGTTTTCAAATTCATTTCGGTTGGAATTAGGCGTTTTGAATAT
A2a intron           ~~CAGCATTCCGTTTATTCGAATGAAAAACACGTTTTA~~~TGTTTCTTTACCATCTGGTTTTCAAATTCATTTCGGTTGGAATTAGGCGTTTTGAATAT
Gamma (A2) 2-036     ~~CAGCATTCCGTTTATTCGAATGAAAAACAAGTTTTA~~~TGTTTCTTTACCATCTGGTTTTCAAATTCATTTCGGTTGGAATTGGGCGTTTTGAATAT
Gamma (G2) 10-010    ~~CAGCATTCCGTTTATTCGAATGAAAAACACGTTTTA~~~TGTTTCTTTACCATCTGGTTTTCAAATTCATTTCGGTTGGAATTGGGCGTTTTGAATAT
Gamma (G3) 10-013    ~~CAGCATTCCGTTTATTCGAATGAAAAACACGTTTTA~~~TGTTTCTTTACCATCTGGTTTTCAAATTCATTTCGGTTGGAATTGGGCGTTTTGAATAT
Gamma (E10) 4-1528   ~~CAGCATTCCGTTTATTCGAATGAAAAACACGTTTTA~~~TGTTTCTTTACCATCTGGTTTTCAAATTCATTTCGGTTGGAATTGGGCGTTTTGAATAT
Delta (E2) 2-020     ~~~~~~~~~~~~~~~~~~~~ATGAAAAAA~ATGTTTCA~~~TGGTTCTTTACAATCTGGTTTTCAAATTCAATTCGGTTAAAATTAGGCGTTTTGAATAT
Delta (E3) 2-067     ~~~~~~~~~~~~~~~~~~~~ATGAAAAAA~ATGTTTCA~~~TGGTTCTTTACAATCTGGTTTTCAAATTCAATTCGTTTAGAATTAGGCGTTTTGAATAT
Delta (E7) 2-073     ~~~~~~~~~~~~~~~~~~~~ATGAAAAAA~ATGTTTCA~~~TGGTTCTTTACAATCTGGTTTTCAAATTCAATTCGGTTAAAATTAGGCGTTTTGAATAT
Delta (E9) 2-090     ~~~~~~~~~~~~~~~~~~~~ATGAAAAAA~ATGTTTCA~~~TGGTTCTTTACAATCTGGTTTTCAAATTCAATTCGTTTAGAATTAGGCGTTTTGAATAT
Delta (08) 2-050     ~~~~~~~~~~~~~~~~~~~~ATGAAAAAA~ATGTTTCA~~~TGGTACTTTACAATCTGGTTTTCAAATTCAATTCGGTTAAAATTAGGCGTTTTGAATAT
E2 intron            ~~~~~~~~~~~~~~~~~~~~ATGAAAAAA~ATGTTTCA~~~TGGTTCTTTACAATCTGGTTTTCATATTCAATTCGTTTAGAATTAGGCGTTTTGAATAT
E2a intron           ~~~~~~~~~~~~~~~~~~~~ATGAAAAAA~ATGTTTCA~~~TGGTTCTTTACAATCTGGTTTTCATATTCAATTCGTTTAGAATTAGGCGTTTTGAATAT
E2b intron           ~~~~~~~~~~~~~~~~~~~AATGAAAAAA~ATGTTTCA~~~TGGTACTTTACAATCTGGTTTTCAAATTCAATTCGGTTAAAATTAGGCGTTTTGAATAT
01 intron            ~~~~~~~~~~~~~~~~~~~~ATGAAAAAA~ATGTTTCA~~~TGGTACTTTACAATCTGGTTTTCAAATTCAATTCGGTTAAAATTAGGCGTTTTGAATAT
```

```
                             310       320       330       340       350       360       370       380       390       400
                    ....|....|....|....|....|....|....|....|....|....|....|....|....|....|....|....|....|....|....|....|
Alpha (B3) 10-017   CGAACCGA~~~~~~~~~~~~~~~~~~~~~~~~~~~~~~~~~~~~~~~~~~~~~~~~~~~~~~~~~~~~~~~~~~~~~~~~~TAGGGTTAAACATAAA~~GG
Alpha (B7) 10-054   CGAACCGA~~~~~~~~~~~~~~~~~~~~~~~~~~~~~~~~~~~~~~~~~~~~~~~~~~~~~~~~~~~~~~~~~~~~~~~~~TAGGGTTAAACACAAA~~GG
Alpha (C5) 4-1536g  CGAACCGA~~~~~~~~~~~~~~~~~~~~~~~~~~~~~~~~~~~~~~~~~~~~~~~~~~~~~~~~~~~~~~~~~~~~~~~~TAGGGTTAAACACAAA~~GG
Alpha (C3) 10-050   CGAACCGA~~~~~~~~~~~~~~~~~~~~~~~~~~~~~~~~~~~~~~~~~~~~~~~~~~~~~~~~~~~~~~~~~~~~~~~~TAGGGTTAAACACAAA~~GG
Alpha (D6) 10-024   CGAACCGA~~~~~~~~~~~~~~~~~~~~~~~~~~~~~~~~~~~~~~~~~~~~~~~~~~~~~~~~~~~~~~~~~~~~~~~~TAGGGTTAA~CACAAA~~GG
Alpha (C2) 4-1545   CGAACCGA~~~~~~~~~~~~~~~~~~~~~~~~~~~~~~~~~~~~~~~~~~~~~~~~~~~~~~~~~~~~~~~~~~~~~~~~TAGGGTTAAACACAAA~~GG
Alpha (D7) 2-005    CGAACCGA~~~~~~~~~~~~~~~~~~~~~~~~~~~~~~~~~~~~~~~~~~~~~~~~~~~~~~~~~~~~~~~~~~~~~~~~TAGGGTTAAACACAAAAAGG
D1f intron          CGAACCGA~~~~~~~~~~~~~~~~~~~~~~~~~~~~~~~~~~~~~~~~~~~~~~~~~~~~~~~~~~~~~~~~~~~~~~~~TAGGGTTAAACATAAA~~GG
D1h intron          CGAACCGA~~~~~~~~~~~~~~~~~~~~~~~~~~~~~~~~~~~~~~~~~~~~~~~~~~~~~~~~~~~~~~~~~~~~~~~~TAGGGTTAAACATAAA~~GG
D1e intron          CGAACCGA~~~~~~~~~~~~~~~~~~~~~~~~~~~~~~~~~~~~~~~~~~~~~~~~~~~~~~~~~~~~~~~~~~~~~~~~TAGGGTTAAACATAAA~~GG
D1d intron          CGAACCGA~~~~~~~~~~~~~~~~~~~~~~~~~~~~~~~~~~~~~~~~~~~~~~~~~~~~~~~~~~~~~~~~~~~~~~~~TAGGGTTAAACACAAA~~GG
D1y intron          CGAACCGA~~~~~~~~~~~~~~~~~~~~~~~~~~~~~~~~~~~~~~~~~~~~~~~~~~~~~~~~~~~~~~~~~~~~~~~~TAGGGTTAAACACAAA~~GG
D1g intron          CGAACCGA~~~~~~~~~~~~~~~~~~~~~~~~~~~~~~~~~~~~~~~~~~~~~~~~~~~~~~~~~~~~~~~~~~~~~~~~TAGGGTTAAACACAAA~GGG
D1b intron          CGAACCGA~~~~~~~~~~~~~~~~~~~~~~~~~~~~~~~~~~~~~~~~~~~~~~~~~~~~~~~~~~~~~~~~~~~~~~~~TAGGGTTAAACACAAA~~GG
C4 intron           CGAACCGC~~~~~~~~~~~~~~~~~~~~~~~~~~~~~~~~~~~~~~~~~~~~~~~~~~~~~~~~~~~~~~~~~~~~~~~~~ATGTACA~~~~~~~~~~~~~~~~~~~~~~~
B8 intron           CGAACCGC~~~~~~~~~~~~~~~~~~~~~~~~~~~~~~~~~~~~~~~~~~~~~~~~~~~~~~~~~~~~~~~~~~~~~~~~~ACTTACGGATTAGGATTAAACATAAA~~GG
B8a intron          CGAACCGC~~~~~~~~~~~~~~~~~~~~~~~~~~~~~~~~~~~~~~~~~~~~~~~~~~~~~~~~~~~~~~~~~~~~~~~~~ATTTACGGATTAGGATTAAACATAAA~~GG
Beta (B5) 10-011    CGAACCGC~~~~~~~~~~~~~~~~~~~~~~~~~~~~~~~~~~~~~~~~~~~~~~~~~~~~~~~~~~~~~~~~~~~~~~~~~ATTTACGGATTAGGATTAAACAATAT~~~~
Beta (B3) 4-2415    CGAACCGC~~~~~~~~~~~~~~~~~~~~~~~~~~~~~~~~~~~~~~~~~~~~~~~~~~~~~~~~~~~~~~~~~~~~~~~~~ATTTACGGATTAGGATTAAACATAGA~~GG
Beta (B8) 10-042    CGAACCGC~~~~~~~~~~~~~~~~~~~~~~~~~~~~~~~~~~~~~~~~~~~~~~~~~~~~~~~~~~~~~~~~~~~~~~~~~ATTTACGGATTAGGATTAAACAATAT~~~~
Beta (F1) 02-006    CGAACCGC~~~~~~~~~~~~~~~~~~~~~~~~~~~~~~~~~~~~~~~~~~~~~~~~~~~~~~~~~~~~~~~~~~~~~~~~~ATTTACGGATTAGGATTAAACATAAA~~GG
Epsilon (B6) 10-007 CGAACAAC~~~~~~~~~~~~~~~~~~~~~~~~~~~~~~~~~~~~~~~~~~~~~~~~~~~~~~~~~~~~~~~~~~~~~~~~~ATTTGAGGAGTAAAGTTAAACATAAA~~GG
Epsilon (D5) 10-004 CGAACAAC~~~~~~~~~~~~~~~~~~~~~~~~~~~~~~~~~~~~~~~~~~~~~~~~~~~~~~~~~~~~~~~~~~~~~~~~~ATTTGAGGAGTAAAGTTAAACATAAA~~GG
Epsilon (01) 10-028 CGAACAAC~~~~~~~~~~~~~~~~~~~~~~~~~~~~~~~~~~~~~~~~~~~~~~~~~~~~~~~~~~~~~~~~~~~~~~~~~ATTTGAGGAGTAAAGTTAAACATAAA~~GG
A2 intron           CCAACCGCATGTATTCTGA~~~~~~~~~~~~~~~~~~~~~~~~~~~~~~~~~~~~~~~~~~~~~~~~~~~~~~~~~~~~~TAGGGTTAAAC~TGAAC~AG
A2a intron          CCAACCGCATGTATTCTGA~~~~~~~~~~~~~~~~~~~~~~~~~~~~~~~~~~~~~~~~~~~~~~~~~~~~~~~~~~~~~TAGGGTTAAACTGAACA~~G
Gamma (A2) 2-036    CCAACCGCATGTATTCTGA~~~~~~~~~~~~~~~~~~~~~~~~~~~~~~~~~~~~~~~~~~~~~~~~~~~~~~~~~~~~~TAGGGTTAAACTGAAC~AGGAACAG
Gamma (G2) 10-010   CCAACCGCATGTATTCTGA~~~~~~~~~~~~~~~~~~~~~~~~~~~~~~~~~~~~~~~~~~~~~~~~~~~~~~~~~~~~~TAGGGTTAAACTGAAC~AGGAACAG
Gamma (G3) 10-013   CCAACCGCATGTATTCTGA~~~~~~~~~~~~~~~~~~~~~~~~~~~~~~~~~~~~~~~~~~~~~~~~~~~~~~~~~~~~~TAGGGTTAAACTGAAC~AGGAACAG
Gamma (E10) 4-1528  CCAACCGCATGTATTCTGA~~~~~~~~~~~~~~~~~~~~~~~~~~~~~~~~~~~~~~~~~~~~~~~~~~~~~~~~~~~~~TAGGGTTAAACTGAAC~AGGAACAG
Delta (E2) 2-020    CCAACCGCATGCATGCATTCTGGTAGAGTTAAACATAACGGGACCAGACATCGAAGAAGACAAATCTCCTA~~~~~~~~~~~~~~~~~~~~~~~~~~~~~
Delta (E3) 2-067    CCAACCGCATGCATGCATTCTGGTAGAGTTAAACATAACGGGACCAGACATCGAAGAAGACAAATCTCCTA~~~~~~~~~~~~~~~~~~~~~~~~~~~~~
Delta (E7) 2-073    CCAACCGCATGCATGCATTCTGGTAGAGTTAAACATAACGGGACCAGACATCGAAGAAGACAAATCTCCTA~~~~~~~~~~~~~~~~~~~~~~~~~~~~~
Delta (E9) 2-090    CCAACCGCATGCATGCATTCTGGTAGAGTTAAACATAACGGGACCAGACATCGAAGAAGACAAATCTCCTA~~~~~~~~~~~~~~~~~~~~~~~~~~~~~
Delta (08) 2-050    CCAACCGCATGCATGCATTCTGGTAGAGTTAAACATAACGGGACCAGACATCGAAGAAGACAAATCTCCTA~~~~~~~~~~~~~~~~~~~~~~~~~~~~~
E2 intron           CCAACCGCATGCATGCATTCTGGTAGAGTTAAACATAACGGGACCAGGCATCGAAGAAGACAAATCTCCTA~~~~~~~~~~~~~~~~~~~~~~~~~~~~~
E2a intron          CCAACCGCATGCATGCATTCTGGTAGAGTTAAACATAACGGGACCAGACATCGAAGAAGACAAATCTCCTA~~~~~~~~~~~~~~~~~~~~~~~~~~~~~
E2b intron          CCAACCGCATGCATGCATTCTGGTAGAGTTAAACATAACGGGACCAGACATCGAAGAAGACAAATCTCCTA~~~~~~~~~~~~~~~~~~~~~~~~~~~~~
01 intron           CCAACCGCATGCATGCATTCTGGTAGAGTTAAACATAACGGGACCAGACATCGAAGAAGACAAATCTCCTA~~~~~~~~~~~~~~~~~~~~~~~~~~~~~
```

```
                              410       420       430       440       450       460       470       480       490       500
                      ....|....|....|....|....|....|....|....|....|....|....|....|....|....|....|....|....|....|....|....|
Alpha (B3) 10-017     GAAAAA~CATCAAAGAAGGCGAGTTACCATTCTTATTTGTCACCAGCCATAACCCAATGTAGAGCTAAAGATAATAATGCAGAATGTGATCAT~~~~~TT
Alpha (B7) 10-054     GAAAAAACATCAAAGAAGGCGAGTTACCATTCTTATTTGTCACCTGCCATAACCCAATGTAGAGCTAAAGATAATAATGCAGAATGTGATCAC~~~~~TT
Alpha (C5) 4-1536g    GAAAAAACATCAAAGAAGGCGAGTTACCATTCTTATTTGTCACCTGCCATAACCCAATGTAGAGCTAAAGATAATAATGCAGAATGTGATCAC~~~~~TT
Alpha (C3) 10-050     GAAAAAACATCAAAGAAGGCGAGTTACCATTCTTATTTGTCACCTGCCATAACCCAATGTAGAGCTAAAGATAATAATGCAGAATGTGATCAC~~~~~TT
Alpha (D6) 10-024     GAAAAAACATCAAAGAAGGCGAGTTACCATTTTTATTTGTCACCTGCCATAACCCAATGTAGAGCTAAAGATAATAATGCAGAATGTGATCAC~~~~~TT
Alpha (C2) 4-1545     GAAAAAACATCAAAGAAGGCGAGTTACCATTCTTATTTGTCACCTGCCATAACCCAATGTAGAGCTAAAGATAATAATGCAGAATGTGATCAC~~~~~TT
Alpha (D7) 2-005      GAAAAAACATCAAAGAAGGCGAGTTACCATTCTTATTTGTCACCTGCCATAACCCAATGTAGAGCTAAAGATAATAATGCAGAATGTGATCAC~~~~~TT
D1f intron            GAAAAAACATCAAAGAAGGCGAGTTACCATTCTTATTTGTCACCTGCCATAACCCAATGTAGAGCTAAAGATAATAATGCAGAATGTGATCAC~~~~~TT
D1h intron            GAAAAAACATCAAAGAAGGCGAGTTACCATTCTTATTTGTCACCTGCCATAACCCAATGTAGAGCTAAAGATAATAATGCAGAATGTGATCAC~~~~~TT
D1e intron            GAAAAAACATCAAAGAAGGCGAGTTACCATTCTTATTTGTCACCTGCCATAACCCAATGTAGAGCTGAAGATAATAATGCAGAATGTGATCAC~~~~~TT
D1d intron            GAAAAAACATCAAAGAAGGCGAGTTACCATTCTTATTTGTCACCTGCCATAACCCAATGTAGAGCTAAAGATAATAATGCAGAATGTGATCAC~~~~~TT
D1y intron            GAAAAAACATCAAAGAAGGCGAGTTACCATTCTTATTTGTCACCTGCCATAACCCAATGTAGAGCTAAAGATAATAATGCAGAATGTGATCAC~~~~~TT
D1g intron            AAAAAAACATCAAAGAAGGCGAGTTACCATTCTTATTTGTCACCTGCCATAACCCAATGTAGAGCTAAAGATAATAATGCAGAATGTGATCAC~~~~~TT
D1b intron            GAAAAAACATCAAAGAAGGCGAGTTACCATTCTTATTTGTCACCTGCCATAACCCAATGTAGAGCTAAAGATAATAATGCAGAATGTGATCAC~~~~~TT
C4 intron             GAAAAA~~~~~~~~~GACAAGTGTTATCAATCTTATTTGTCACCGAGCATAAACCAATGTAGAGCCAAAGATAATAACGCAGAATGTGA~~~~~~~~~TT
B8 intron             GAAAAAA~~TATAAGAAGGCGAGTTACCATTCTTATTTGTCACCTGCTATAAACCAATGTAGAGCCAAATATAATAATGCAGAATGTGATTAC~~~~~TT
B8a intron            GAAAAA~~~TATAAGAAGGCGAGTTACCATTCTTATTTGTCACCTGCTATAAACCAATGTAGAGCAAAATATAATAATGCAGAATGTGATTAC~~~~~TT
Beta (B5) 10-011      ~~~~~~~~~~~~AAGAAGGCGACTTACCATTCTTATTTGTCACCTGCTATAAACCAATGTAGAGCCAAATATAATAATGCAGAATGTGATTAC~~~~~TT
Beta (B3) 4-2415      GAAAAAA~~TATAAGAAGGCGAGTTACCATTCTTATTTGTCACCTGCTATAAACCAATGTAGAGCCAAATATAATAATGCAGAATGTGATTAC~~~~~TT
Beta (B8) 10-042      ~~~~~~~~~~~~AAGAAGGCGACTTACCATTCTTATTTGTCACCTGCTATAAACCAATGTAGAGCCAAATATAATAATGCAGAATGTGATTAC~~~~~TT
Beta (F1) 02-006      GAAAAAA~~TATAAGAAGGCGAGTTACCATTCTTATTTGTCACCTGCTATAAACCAATGTAAAGCCAAATATAATAATGCAGAATGTGATTAC~~~~~TT
Epsilon (B6) 10-007   GAAAAAACATCAAAGAAGTCAAAGAAGG~~~~~~~~~~~~~~~~~~~~~~~~~~CAATGTAGAGCTAAATATAAAAATGCAGAATGTAATTAC~~~~~TT
Epsilon (D5) 10-004   GAAAAAACATCAAAGAAGTCAAAGAAGG~~~~~~~~~~~~~~~~~~~~~~~~~~CAATGTAGAGCTAAATATAAAAATGCAGAATGTAATTAC~~~~~TT
Epsilon (01) 10-028   GAAAAAACATCAAAGAAGTCAAAGAAGG~~~~~~~~~~~~~~~~~~~~~~~~~~CAATGTAGAGCTAAATATAAAAATGCAGAATGTAATTAC~~~~~TT
A2 intron             GAACAGACATCAAAGAAGGCGAGTTACCATTCTTATTTGTCACCTGCAAAAAACCAATGTAGAGCCAAATATAAAAATGCAGAATGTGATTAC~~~~~TT
A2a intron            GAACAGACATCGAAGAAGACAAGTGTTATCAATCATTTGTCACCTAGCATATCCCAATGTAGAGCTAAATATCATAATGCAGAATGTGA~~~~~~~~~TT
Gamma (A2) 2-036      ACATC~~~~~~AAAGAAGGCGAGTTACCATTCTTATTTGCCACCTGCAAAAAACCAATGTAGAGCCAAATATAAAAATGCAGAATGTGATTAC~~~~~TT
Gamma (G2) 10-010     ACATC~~~~~~GAAGAAGACAAGTGTTATCAATCATTTGTCACCTAGCATAACCCAATGTAGACCTAAATATCATAATGCAGAATGTGA~~~~~~~~~TT
Gamma (G3) 10-013     ACATC~~~~~~GAAGAAGACAAGTGTTATCAATCATTCGTCACCTAGCATAACCCAATGTAGACCTAAATATCATAATGCAGAATGTGA~~~~~~~~~TT
Gamma (E10) 4-1528    ACATC~~~~~~GAAGAAGACAAGTGTTATCAATCATTTGTCACCTAGCATAACCCAATGTAGACCTAAATATCATAATGCAGAATGTGA~~~~~~~~~TT
Delta (E2) 2-020      ~~~~~~~~~~~~~~~~~~~~~~~~~~~~~~~~~~~~~~~~~~~~~~GCATAAACCAATGTAGAGCTAAAGATAATAATGCAGAATGTGATTAATTTTATT
Delta (E3) 2-067      ~~~~~~~~~~~~~~~~~~~~~~~~~~~~~~~~~~~~~~~~~~~~~~GCATAAACCAATGTAGAGCTAAAGATAATAATGCAGAATGTGATTAATTTTATT
Delta (E7) 2-073      ~~~~~~~~~~~~~~~~~~~~~~~~~~~~~~~~~~~~~~~~~~~~~~GCATAAACCAATGTAGAGCTAAAGATAATAATGCAGAATGTGATTAATTTTATT
Delta (E9) 2-090      ~~~~~~~~~~~~~~~~~~~~~~~~~~~~~~~~~~~~~~~~~~~~~~GCATAAACCAATGTAGAGCTAAAGATAATAATGCAGAATTTGATTAATTTTATT
Delta (08) 2-050      ~~~~~~~~~~~~~~~~~~~~~~~~~~~~~~~~~~~~~~~~~~~~~~GCATAAACCAATGTAGAGCTAAAGATAATAATGCAGAATGTTATTAATTTTATT
E2 intron             ~~~~~~~~~~~~~~~~~~~~~~~~~~~~~~~~~~~~~~~~~~~~~~GCATAAACCAATGTAGAGCTAAAGATAATAATGCAGAATGTGATTAATTTTATT
E2a intron            ~~~~~~~~~~~~~~~~~~~~~~~~~~~~~~~~~~~~~~~~~~~~~~GCATAAACCAATGTAGAGCTAAAGATAATAATGCAGAATGTGATTAATTTTATT
E2b intron            ~~~~~~~~~~~~~~~~~~~~~~~~~~~~~~~~~~~~~~~~~~~~~~GCATAAACCAATGTAGAGCTAAAGATAATAATGCAGAATGTGATTAATTTTATT
01 intron             ~~~~~~~~~~~~~~~~~~~~~~~~~~~~~~~~~~~~~~~~~~~~~~GCATAAACCAATGTAGAGCTAAAGATAATAATGCAGAATGTGATTAATTATTTT
```

```
                              510       520       530
                        ....|....|....|....|....|....|....|..
Alpha (B3) 10-017       AATTAATTCGATTTTTTT~~CTTCATTATCCCCTACA
Alpha (B7) 10-054       AATTAATTCGATTTTTTT~~CTTCATTATCCCCTACA
Alpha (C5) 4-1536g      AATTAATTCGATTTTTTT~~CTTCATTATCCCCTACA
Alpha (C3) 10-050       GATTAATTCGATTTTATTT~~TTCATTATCCCCTACA
Alpha (D6) 10-024       AATTAATTCGATTTTTTT~~CTTCATTATCCCCTACA
Alpha (C2) 4-1545       AATTAATTCGATTTTTTT~~CTTCATTATCCCCTACA
Alpha (D7) 2-005        AATTAATTCGATTTTTTTT~CTTCATTATCCCCTACA
D1f intron              AATTAATTCGATTTTATT~~CTTCATTATCCCCTACA
D1h intron              AATTAATTCGATTTTATT~~CTTCATTATCCCCTACA
D1e intron              AATTAATTCGATTTGTATTT~TTCATTATCCCCTACA
D1d intron              AATTAATTCGATTTTTT~~~CTTCATTATCCCCTACA
D1y intron              AATTAATTCGATTTTTTT~~CTTCATTATCCCCTACA
D1g intron              AATTAACTCGATTTTTTT~~CTTCATTATCCCCTACA
D1b intron              AATTAATTCGATTTTTTT~~CTTCATTATCCCCTACA
C4 intron               AATTAGTTCGATGAT~~~~~~TTCATTACCCATTACA
B8 intron               AATTAATTCGATTAT~~~~~~TTCATTAACCCTTACA
B8a intron              AATTAATTCGATTAT~~~~~~TTCATTAACCCTTACA
Beta (B5) 10-011        AATTAATTCGATTAT~~~~~~TTCATTAACCCTTACA
Beta (B3) 4-2415        AATTAATTCGATTAT~~~~~~TTCATTAACCCTTACA
Beta (B8) 10-042        AATTAATTCGATTAT~~~~~~TTCATTAACCCTTACA
Beta (F1) 02-006        AATTAATTCGATTAT~~~~~~TTCATTAACCCTTACA
Epsilon (B6) 10-007     AATTAATTCGATTGT~~~~~~TTCATTACCCCTTACA
Epsilon (D5) 10-004     AATTAATTCGATTGT~~~~~~TTCATTACCCCTTACA
Epsilon (01) 10-028     AATTAATTCGATTGT~~~~~~TTCATTACCCCTTACA
A2 intron               AATTAACTCGATTTTT~~~~~~~CATTACCCCTTACA
A2a intron              AATTAGTTCGATGATTT~~~~~~CATTACCCCCTACA
Gamma (A2) 2-036        AATTAATTCGA~~~TTT~~~~TTCATTACCCCTTACA
Gamma (G2) 10-010       AATTAGTTCGATGAT~~~~~~TTCATTACCCCCTACA
Gamma (G3) 10-013       AATTAGTTCGATGAT~~~~~~TTCATTACCCCCTACA
Gamma (E10) 4-1528      AATTAGTTCGATGATTT~~~~~~CATTACCCCCTACA
Delta (E2) 2-020        AATAAATACGACTATTT~~~~~~CATTAT~CCCAACA
Delta (E3) 2-067        AATAAATTCGACTATTT~~~~~~CATTAT~CCCAACA
Delta (E7) 2-073        AATAAATTCGACTATTT~~~~~~CATTAT~CCCAACA
Delta (E9) 2-090        AATAAATTCGACTATTT~~~~~~CATTAT~CCCAACA
Delta (08) 2-050        AATAAATTCGACTATTC~~~~~~CATTAT~CCCAACA
E2 intron               AATAAATTCGACTATTT~~~~~~CATTAT~CCCAACA
E2a intron              AATAAATTCGACTATTT~~~~~~CATTAT~CCCAACA
E2b intron              AATAAATTCGACTATTT~~~~~~CATTAT~CCCAACA
01 intron               ATTAATAAATTCGACTATTC~~~CATTAT~CCCAACA
```

Figure S8 | The alignment of *SpTrf* introns. A representative number of introns of each intron type were selected from the set of genes reported by Buckley and Smith (Ref. 19 in the main paper) and used to generate an alignment with the introns from the genes from the four clusters. The intron types and the *SpTrf* genes from which they were obtained (indicated in brackets) and their numerical identification as found in (Buckley and Smith, Ref. 19 in the main paper) are listed to the left. The nucleotide position is indicated above the alignment. The ~ indicates the insertion of artificial gaps in the alignment where the sequences do not match. The alignment was done using PRANK.

```
              10        20        30        40        50         60        70        80        90
      ....|....|....|....|....|....|....|....|....|....|....| ....|....|....|....|....|....|....|....|....
A2    ATGGAGGTGAAAGTGACACTGATCGTTGCCATTGTGGCTGCTCTTGCTATCTCGG^GTAAGAAATCAAATTATTACTTGGTATTACTTGATAAGTGGCAA
A2a   .............CA.........................T..............^............................................

     100       110       120       130       140       150       160       170       180       190
      |....|....|....|....|....|....|....|....|....|....|....|....|....|....|....|....|....|....|....|....
A2    ATATTAAGCCAACAAAAGGCTCACAGGAGTATATTATTATTTCATTTATCATAATATGTGTTTCTTACCTGTTTGTTACACAATACACAAAATATTTCTT
A2a   ............G...G.........................A............................................A............

     200       210       220       230       240       250       260       270       280       290
      |....|....|....|....|....|....|....|....|....|....|....|....|....|....|....|....|....|....|....|....
A2    CTGTGGGCTTCAGCATTCCGTTTATTCGAATGAAAAACACGTTTTATGTTTCTTTACCATCTGGTTTTCAAATTCATTTCGGTTGGAATTAGGCGTTTTG
A2a   ....................................................................................................

     300       310       320       330       340       350       360       370       380       390
      |....|....|....|....|....|....|....|....|....|....|....|....|....|....|....|....|....|....|....|....
A2    AATATCCAACCGCATGTATTCTGATAGGGTTAAACTGAACAGGAACAGACATCAAAGAAGGCGAGTTACCATTCTTATTTGTCACCTGCAAAAAACCAAT
A2a   .....................................................G......A.A...GTTATCAA.C...........AGC.T.TC.....

     400       410       420       430       440       450       460        470       480       490
      |....|....|....|....|....|....|....|....|....|....|....|....|.  ...|....|....|....|....|....|....
A2    GTAGAGCCAAATATAAAAATGCAGAATGTGATTACTTAATTAACTCGATTTTTCATTACCCCTTACA^GCTCACGCACAAAGCGATTTCAATGAACGACGA
A2a   .......T......C.T.................A...G..CGA.~.....~~.........C....^..........G...A.............T....

     500       510       520       530       540       550       560       570       580       590
      |....|....|....|....|....|....|....|....|....|....|....|....|....|....|....|....|....|....|....|....
A2    GGAAAGGAGAATGGCAGAGAGAGAGGACAAGATCGCTTTGGAGGAAGGCCTGATGGAATGCAGATGGGTGGACCTAGGCAAGATGGCGGTCCGATGGGTG
A2a   ............A...................C...................................................................

     600       610       620       630       640       650       660       670       680       690
      |....|....|....|....|....|....|....|....|....|....|....|....|....|....|....|....|....|....|....|....
A2    GTAGGAGATTCGACGGACCTAGATTTGGTGCCCCGCAGATGGGTGGACCTAGGCAAAATGGTGGACCAATGGGTGGCAGAAGGTTCGATGGACCTGGATT
A2a   ............................................................................A.......................

     700       710       720       730       740       750       760       770       780       790
      |....|....|....|....|....|....|....|....|....|....|....|....|....|....|....|....|....|....|....|....
A2    TGGTGCCCCGCCGATGGGTGGACCAAGGCAAGATGGTGGACCAATGGGTGGAAGAAGGTTCGATGGACCTGGATTTGGTGCCCCGCAAATGGGTGGACCT
A2a   ....................................................................................................
```

```
     800       810       820       830       840       850       860       870       880       890
     |....|....|....|....|....|....|....|....|....|....|....|....|....|....|....|....|....|....|....|....
A2   AGGCAAAATGGCGGTCCGATGGGTGGTAGGAGATTCGACGGACCTCGATTTGGTGGCTCCAGACCAGATGGTGCTGGAGGGAGACCTTTCTTCGGCGAAG
A2a  ...........T.................................G..................................A...................

     900       910       920       930       940       950       960       970       980       990
     |....|....|....|....|....|....|....|....|....|....|....|....|....|....|....|....|....|....|....
A2   GAGGTAGGCGTGGTGATGGAGAAGAAGAAACTGATGCTGCCCGACAAATT~~~~~~~~~~~~~~~GGGCCTGGTCGGTTTGATGGTCCTGGACATGGTCA
A2a  ..................................................GGTGATGGTCTAGGA...................................

     1000      1010      1020      1030      1040      1050      1060      1070      1080      1090
     |....|....|....|....|....|....|....|....|....|....|....|....|....|....|....|....|....|....|....
A2   TTATGGTCATCATCAAGGTGCAGGAAGACCTTTCTTCGGCAATCCTCCTCCTTTTAACCCAGAACAGGAACCGCGCAACGACAGCAGCGAGGAGGATGGC
A2a  ....................................................................................................

     1100      1110      1120      1130      1140      1150      1160      1170      1180      1190
     |....|....|....|....|....|....|....|....|....|....|....|....|....|....|....|....|....|....|....
A2   CGTCATCACCGTCACCACGATCGCCACCACGCCCACCATGGCCACCATGGCCACCACGAACACCATCATCAACATCATAACCACACAGAAGGCCACCAAG
A2a  .............T.........................................................T............T...............

     1200      1210      1220      1230      1240      1250      1260      1270      1280      1290
     |....|....|....|....|....|....|....|....|....|....|....|....|....|....|....|....|....|....|....
A2   ATCATGACAGACCGATGTTTGAGATGAGGCCCTTCCGGTTCAACCCCTTGGGTAGAAAGCCTTTCGGAGACCATCCCTTCGGCAGACGCAATCACACAGA
A2a  ....................................................................................................

     1300      1310      1320      1330      1340      1350      1360      1370      1380      1390
     |....|....|....|....|....|....|....|....|....|....|....|....|....|....|....|....|....|....|....
A2   AGGTCACCAGGGTCATAATGAGACGGGAGATCACCCCCACCGTCATCACAGCAAAAACGTAGATGGAGATCAGGACACCGGCCACCACGGCCACCATGGC
A2a  ......................................................G.C...........................................

     1400      1410      1420      1430      1440      1450      1460      1470      1480      1490
     |....|....|....|....|....|....|....|....|....|....|....|....|....|....|....|....|....|....|....
A2   CACCACGAACACCATCATCATCAGCATGACCACAGAGAAGGCCACCAAGATCATGACAGACCGATGTTTGAGATGAGGCCCTTCCGGTTCAACCCCTTGG
A2a  ......................................................................G...........................C.

     1500      1510      1520      1530      1540      1550      1560      1570      1580      1590
     |....|....|....|....|....|....|....|....|....|....|....|....|....|....|....|....|....|....|....
A2   GTAGAAAGCCTTTCGGAGACCATCCCTTCGGCAGACGCAACCACACAGAAGGTCACCAGGGTCATAATGAGACGGGAGATCACCCCCACCGTCATCACAG
A2a  ....................................................................................................

     1600      1610      1620      1630      1640      1650      1660      1670      1680      1690
     |....|....|....|....|....|....|....|....|....|....|....|....|....|....|....|....|....|....|....
A2   CAAGACCGGAGATGGAGATCAGGACAGACCAATGTTTGAGACGAGGCCCTTCTGGGTCAACCCCTTCGGTAGAAAGCCTTTCGGAGACCGTCCCTTCGAC
A2a  ....................................................................................................
```

```
          1700      1710      1720      1730      1740      1750      1760      1770      1780      1790
          |....|....|....|....|....|....|....|....|....|....|....|....|....|....|....|....|....|....|....|....
A2        AGACGCAACGGAACCGAAGAAGGATCTCCCAGGCGTGATGGCCACCCTCATCCCCATGGTAACCGCGGACGTTGGGGTGAGAATGAAAGTGAGGAGAAGG
A2a       ....................................................................................................

          1800      1810      1820      1830      1840      1850      1860      1870      1880      1890
          |....|....|....|....|....|....|....|....|....|....|....|....|....|....|....|....|....|....|....|..
A2        AGCATCCAACGACGGAAAGCGTAACGACATCTTCACCACTTAAAGTGATCGAGATCGCAATCAATGAAGTAGACACCAATGTGGTCGCCGAGGTGTAG
A2a       ..................................................................................................
```

Figure S9 | The alignment of the *A2* genes shows minor sequence variations plus a small indel. The names of the genes are located to the left and above each alignment is a ruler indicating the nucleotide position. Dots in *A2*a indicate a matching nucleotide to *A2*. The ~ indicates the insertion of artificial gaps in the alignment where the sequences do not match. The ^ indicates the start and end of the intron. The alignment was done with ClustalW in BioEdit (ver 7.2.5).

```
               10        20        30        40        50         60        70        80        90
      ....|....|....|....|....|....|....|....|....|....|....| ....|....|....|....|....|....|....|....
B8a   ATGGAGGTGAAAGCAACATTGATCGTTGCCATTGTGGCTGCTCTTGCTATCTCGG^GTAAGAAATCAAATTTT~ACTCGGTATTACTTCATAAGTGCTAA
B8    .......................................................^...............A.T..............G...........

      100       110       120       130       140       150       160       170       180       190
      |....|....|....|....|....|....|....|....|....|....|....|....|....|....|....|....|....|....|....|....
B8a   ATATAAAGCCAATGAAGGGCTCACAGAAGTAAATATGATTATTTCATAGTTTGTAATATGCATTTTTAAATTGTTCGTTACACAATATAATTAATTGTAT
B8    ............C.......................................................................................

      200       210       220       230       240       250       260       270       280       290
      |....|....|....|....|....|....|....|....|....|....|....|....|....|....|....|....|....|....|....|....
B8a   TTATTAAGCCTACACCAATCCGTTTTCTGAATGACAAAGGATGTGTATTATGTTTACGTTCAATCTGGTAATCAAATTTAATTCAGTCAGAATTAGGCGT
B8    ....................................................................................................

      300       310       320       330       340       350       360       370       380       390
      |....|....|....|....|....|....|....|....|....|....|....|....|....|....|....|....|....|....|....
B8a   TTTGAATATCGAACCGCATTTACGGATTAGGATTAAACATAAAGGGAAAAA~TATAAGAAGGCGAGTTACCATTCTTATTTGTCACCTGCTATAAACCAA
B8    ..................C................................A................................................

      400       410       420       430       440       450       460       470       480       490
      |....|....|....|....|....|....|....|....|....|....|....|....|....  .|....|....|....|....|....|....
B8a   TGTAGAGCAAAATATAATAATGCAGAATGTGATTACTTAATTAATTCGATTATTTCATTAACCCTTACA^GCTCACGCACGAAGAGATTTCAATGAACGGC
B8    ........C............................................................^...............................

      500       510       520       530       540       550       560       570       580       590
      |....|....|....|....|....|....|....|....|....|....|....|....|....|....|....|....|....|....|....|....
B8a   GAGGAAATGAGAATGGCAGAGAGAGAGGACAAGGTCGCTTTGGAGGAAGGCCTGGTGGAATGCAGATGGGTGGATCTAGGCAAGATGGTGGACCAATGGG
B8    ....................................................................................................

      600       610       620       630       640       650       660       670       680       690
      |....|....|....|....|....|....|....|....|....|....|....|....|....|....|....|....|....|....|....|....
B8a   TGGAAGGAGGTTCGATGGACCTGGATTTGGTGCCCCACATATGGATGGACGCAGACAAAATGGCGGTCCGATGGGTGGTAGGAGATTCGACGGACCTGGA
B8    ....................................................................................................

      700       710       720       730       740       750       760       770       780       790
      |....|....|....|....|....|....|....|....|....|....|....|....|....|....|....|....|....|....|....|....
B8a   TTTGGTGGCTCCAGACCAGATGGTGCTGGAGGAAGACCTTTCTTCGGCCAAGGAGGAAGGCGTGGTGATGGAGAAGAAGAAACTGATGCTGCCCAACAAA
B8    ...........................................................A..............................T.........

      800       810       820       830       840       850       860       870       880       890
      |....|....|....|....|....|....|....|....|....|....|....|....|....|....|....|....|....|....|....|....
B8a   TTGGTGATGGTCTAGGAGGGCGCGGTCAGTTTGATGGTCATGGACGTCGTCATCATGGTCACCGTCAAGGTCCACCTCAGGACCGACCAGAGGAACAACC
B8    ....................................................................................................
```

```
        900       910       920       930       940       950       960       970       980       990
        |....|....|....|....|....|....|....|....|....|....|....|....|....|....|....|....|....|....|....|....
B8a     GTTCGGCCAGCGCAATGAACGCAACGAGGAGGATGGCCGTCCTCACCCTCACCACCATGGCCACCATGGCCACCATGGCCACCACCACCGTCATCATAAC
B8      .....T................................................T....................A........................

        1000      1010      1020      1030      1040      1050      1060      1070      1080      1090
        |....|....|....|....|....|....|....|....|....|....|....|....|....|....|....|....|....|....|....
B8a     CAGACAGAGGGTCACCAAGGACATAATGAGACGGGAGATCAAGATCAGGACAAACCAAATGATACGAGGCCCTTCCGGTTCAACCACTTCGGCAGACGCA
B8      ....................................................................................................

        1100      1110      1120      1130      1140      1150      1160      1170      1180      1190
        |....|....|....|....|....|....|....|....|....|....|....|....|....|....|....|....|....|....|....
B8a     ACCACACAGAAGGTCACCAGGGTCATAATGAGACGGGAGATCACCCCCATCGTCATCACAACAAGACCGGAGATGGAGATCAGGACAGACCAATGTTTGA
B8      ....................................................................................................

        1200      1210      1220      1230      1240      1250      1260      1270      1280      1290
        |....|....|....|....|....|....|....|....|....|....|....|....|....|....|....|....|....|....|....
B8a     GATGAGGCCCTTCTGGGTCAACCCCTTCGGTAGAAAGCCTTTCGGAGACCGTCCCTTCGACAGACGCAACGGAACCGAAGAAGGATCTCCCAGGCGTGAT
B8      ...........................................................G..........A.............................

        1300      1310      1320      1330      1340      1350      1360      1370      1380      1390
        |....|....|....|....|....|....|....|....|....|....|....|....|....|....|....|....|....|....|....
B8a     GGCCACCGTCATCCCTATGGTAACCGAGGACGTTGGGGTGAGAATGAAAGTGAGGAGAAGGAGCATCCAACGACGGAAAGCATAACGACATCTTCACCAC
B8      .................................................................................G..................

        1400      1410      1420      1430      1440      1450
        |....|....|....|....|....|....|....|....|....|....|...
B8a     CTGAAGTGGTTGAGATCGCAGTCAATGAAGAAGACGTCAATGTGGTCGCCGAGGTGTAC
B8      ...........................................................
```

Figure S10 | The alignment of *B8* genes shows single nucleotide variations throughout the sequences. The names of the genes are listed to the left and above each alignment is a ruler indicating the nucleotide position. Dots in *B8* represent a matching nucleotide to *B8*a. The ~ indicates the insertion of a few artificial gaps in the alignment where the sequences do not match. The ^ indicates the start and end of the intron. The alignment was done using ClustalW in BioEdit (ver 7.2.5).

```
                  10        20        30        40        50         60        70        80        90
          ....|....|....|....|....|....|....|....|....|....|.....| ....|....|....|....|....|....|....|....|....
CL4 C4a   ATGGAGGTGAAAGCAACATTGATCGTTGCCATTGTGGCTGCTCTTGCTATCTCGG^GTAAGAAATCAAATTATTACTCGGTATTACTTGATAAGTGCTAA
CL3 C4     .......................................................^............................................

          100       110       120       130       140       150       160       170       180       190
          |....|....|....|....|....|....|....|....|....|....|....|....|....|....|....|....|....|....|....|....
CL4 C4a   ATATAAAGCCAACGAAGGGTTCACAGAAGTAAATATGATTATTTCATAGTTTGTAATATGCATTTTTAAATTGTTCGTTACAAATTATAATTAATTTTAT
CL3 C4    ....................................................................................................

          200       210       220       230       240       250       260       270       280       290
          |....|....|....|....|....|....|....|....|....|....|....|....|....|....|....|....|....|....|....|....
CL4 C4a   TTCTTAAACCTACACCAATCCGTTGGTCGAATGACAAAAGATGTGTATTATGTTTACGTTCAATCTGGTATTCAAATTTAATTCAGTCAGAATAGGGCAT
CL3 C4    ....................................................................................................

          300       310       320       330       340       350       360       370       380       390
          |....|....|....|....|....|....|....|....|....|....|....|....|....|....|....|....|....|....|....|....
CL4 C4a   TTTGAAGGTCGAACCGCATGTACAGAAAAAGACAAGTGTTATCAATCTTATTTGTCACCGAGCATAAACCAATGTAGAGCCAAAGATAATAACGCAGAAT
CL3 C4    ....................................................................................................

          400       410       420       430        440       450       460       470       480       490
          |....|....|....|....|....|....|....|.. ..|....|....|....|....|....|....|....|....|....|....|....|....
CL4 C4a   GTGATTAATTAGTTCGATGATTTCATTACCCATTACA^GCTCACGCACGAAGAGATTTTAATGAACGACGAGGACGGGAGAATGGTAGAAAGAGGGGACAA
CL3 C4    .....................................^...............................................................

          500       510       520       530       540       550       560       570       580       590
          |....|....|....|....|....|....|....|....|....|....|....|....|....|....|....|....|....|....|....|....
CL4 C4a   GGTGGCTTTGGAGGAAGGCCTGATGGAATGCAGATGGGTGGTAGGAGATTCGACGGACCTGGATTTGGTGGCTCCAGACCAGATGGCGCTGGAAGACCTT
CL3 C4    ....................................................................................................

          600       610       620       630       640       650       660       670       680       690
          |....|....|....|....|....|....|....|....|....|....|....|....|....|....|....|....|....|....|....|....
CL4 C4a   TCTTCGGCCAAGGAGGCAAGCGTGGTGATGGAGAAGAAGAAACTGATGCTGCCCAACAAATTGGTGATGGTCTAGGAGGGTCCGATCGGTTTGATGGTCC
CL3 C4    ....................................................................................................

          700       710       720       730       740       750       760       770       780       790
          |....|....|....|....|....|....|....|....|....|....|....|....|....|....|....|....|....|....|....|....
CL4 C4a   TAGACGTGGTCATCATGGTCACCGTCAAGGTCCACCTCAGGATCGACCAGAGGAACAACCGTTCGGTCAGCGCAATGAAAGCAGCGAGGAGGATGGCCGT
CL3 C4    ....................................................................................................

          800       810       820       830       840       850       860       870       880       890
          |....|....|....|....|....|....|....|....|....|....|....|....|....|....|....|....|....|....|....|....
CL4 C4a   CCTCACCCTCACCACCACGGCCACCATCGCCACCATCGCCACCACCATCATCATAACCAGACAGAAGGTCACCAAGGTCATAATGACACGGGAGATCAAG
CL3 C4    ....................................................................................................
```

```
            900       910       920       930       940       950       960       970       980       990
            |....|....|....|....|....|....|....|....|....|....|....|....|....|....|....|....|....|....|....|....
CL4 C4a     ATCAGGACAAACCAAATGATACAAGGCCCTTCCGGTTCAACCACTTCGGTAGAAAGCCTTTCGGAGACCGTCCCTTCGGCAGACGCAACCACACAGAAGG
CL3 C4      ....................................................................................................

           1000      1010      1020      1030      1040      1050      1060      1070      1080      1090
            |....|....|....|....|....|....|....|....|....|....|....|....|....|....|....|....|....|....|....|....
CL4 C4a     TCACCAGGGTCATAATGAGACGGGAGATCACCCCCACCGTCATCACAACCAGACCGGAGATGGAGATCAGGACAGACCGATGTTTGAGATGAGGCCCTTC
CL3 C4      ....................................................................................................

           1100      1110      1120      1130      1140      1150      1160      1170      1180      1190
            |....|....|....|....|....|....|....|....|....|....|....|....|....|....|....|....|....|....|....|....
CL4 C4a     CGGTTCAACCCCTTGGGTAGAAAGCCTTTCGGAGACCGTCCCTTCGGCAGACGCAACGGAACCGAAGAAGGATCTCTCAGGCGTGATGGCCATCGTCGGC
CL3 C4      ....................................................................................................

           1200      1210      1220      1230      1240      1250      1260      1270      1280      1290
            |....|....|....|....|....|....|....|....|....|....|....|....|....|....|....|....|....|....|....|....
CL4 C4a     CCTATGGTAACCGAGGACGTTGGGGTGAGAATGAAAGTGAGGAGAAGGAGCATCCAACGACGGAAAGCGTAACGACATCTTCACCACCTGACGTGGTTGA
CL3 C4      ....................................................................................................

           1300      1310      1320      1330      1340
            |....|....|....|....|....|....|....|....|....|
CL4 C4a     GATCGCAGTCAATGAAGAAGACGTCAATGTGGTCGCCGAGGTGTAG
CL3 C4      ..............................................
```

Figure S11 | The alignment of the *C4* genes shows that the two genes are identical. The names of the genes are listed to the left with their cluster number is indicated by CL. Above each alignment is a ruler indicating the nucleotide position. Each nucleotide is represented in a distinct color and a corresponding dot in *C4* represents a matching nucleotide to *C4*a. The ^ indicates the start and end of the intron. The alignment was done with ClustalW in BioEdit (ver 7.2.5).

Score	Expect	Identities	Gaps	Strand
1646 bits(891)	0.0	942/966(98%)	6/966(0%)	Plus/Plus

```
Query  22   ATGGAGGTGAAAGCAACATTGATCGTTGCCATTGTGGCTGCTCTTGCTATCTCGGCTCAC  81
            ||||||||||||||||||||||||||||||||||||||||||||||||||||||||||||
Sbjct  1    ATGGAGGTGAAAGCAACATTGATCGTTGCCATTGTGGCTGCTCTTGCTATCTCGGCTCAC  60

Query  82   GCACGAAGAGATTTCAATGAACGACGAGGACGGGAGAATGGTAGAGAGAGGGGACAAGGT  141
            |||||||||||||| |||||||||||||||||||||||||||||| ||||||||||||||
Sbjct  61   GCACGAAGAGATTTTAATGAACGACGAGGACGGGAGAATGGTAGAAAGAGGGGACAAGGT  120

Query  142  GGGTTTGGAGGAAGGCCTGATGGAATGCAGATGGGTGGTAGGAGATTCGACGGACCTGGA  201
            || |||||||||||||||||||||||||||||||||||||||||||||||||||||||||
Sbjct  121  GGCTTTGGAGGAAGGCCTGATGGAATGCAGATGGGTGGTAGGAGATTCGACGGACCTGGA  180

Query  202  TTTGGTGGCTCCAGACCAGTTGGTGCTGGAGGAAGACCTGTCTTCGGCCAAGGAGGCAAG  261
            ||||||||||||||||||| ||| |||   ||||||||| ||||||||||||||||||||
Sbjct  181  TTTGGTGGCTCCAGACCAGATGGCGCT---GGAAGACCTTTCTTCGGCCAAGGAGGCAAG  237

Query  262  CGTGGTGATGGAGAAGAAGAAACTGATGCTGCCCAACAAATTGGTGATGGTCTAGGAGGG  321
            ||||||||||||||||||||||||||||||||||||||||||||||||||||||||||||
Sbjct  238  CGTGGTGATGGAGAAGAAGAAACTGATGCTGCCCAACAAATTGGTGATGGTCTAGGAGGG  297

Query  322  CCCGGTCAGTTTGATGGTCCTAGACGTGGTCATCATGGTCACCGTCAAGGTCCACCTCAG  381
             ||| || ||||||||||||||||||||||||||||||||||||||||||||||||||||
Sbjct  298  TCCGATCGGTTTGATGGTCCTAGACGTGGTCATCATGGTCACCGTCAAGGTCCACCTCAG  357

Query  382  GATCGACCAGAGGAACAACCGTTCGGTCAGCGCAATGAAAGCAGCGAGGAGGATGGCCGT  441
            ||||||||||||||||||||||||||||||||||||||||||||||||||||||||||||
Sbjct  358  GATCGACCAGAGGAACAACCGTTCGGTCAGCGCAATGAAAGCAGCGAGGAGGATGGCCGT  417

Query  442  CCtcaccctcaccaccacggccaccatcgccaccatcgccaccaccatcatcataaccaG  501
            ||||||||||||||||||||||||||||||||||||||||||||||||||||||||||||
Sbjct  418  CCTCACCCTCACCACCACGGCCACCATCGCCACCATCGCCACCACCATCATCATAACCAG  477

Query  502  ACAGAAGGTCACCAAGGTCATAATGAGACGGGAGATCAAGATCAGGACAACCCAAATGAT  561
            |||||||||||||||||||||||||| ||||||||||||||||||||||| |||||||||
Sbjct  478  ACAGAAGGTCACCAAGGTCATAATGACACGGGAGATCAAGATCAGGACAAACCAAATGAT  537

Query  562  ACAAGGCCCTTCAAGTTCAACCACTTCGGTAGAAAGCCTTTCGGAGACCGTCCTTTCGGC  621
            ||||||||||||  ||||||||||||||||||||||||||||||||||||||| ||||||
Sbjct  538  ACAAGGCCCTTCCGGTTCAACCACTTCGGTAGAAAGCCTTTCGGAGACCGTCCCTTCGGC  597
```

```
Query  622  AGACGCAACCACACAGAAGGTCACCAGGGTCATAATGAGACGGGAGATCACCCCCACCGT  681
            ||||||||||||||||||||||||||||||||||||||||||||||||||||||||||||
Sbjct  598  AGACGCAACCACACAGAAGGTCACCAGGGTCATAATGAGACGGGAGATCACCCCCACCGT  657

Query  682  CATCACAACCAGACCGGAGATGGAGATCAGGACAGACCGATGTTTGAGATGAGGCCCTTC  741
            ||||||||||||||||||||||||||||||||||||||||||||||||||||||||||||
Sbjct  658  CATCACAACCAGACCGGAGATGGAGATCAGGACAGACCGATGTTTGAGATGAGGCCCTTC  717

Query  742  CGGTTCAACCCCTTGGGTAGAAAGCCTTTCGGAGACCGTCCCTTCGGCAGACGCAACGGA  801
            ||||||||||||||||||||||||||||||||||||||||||||||||||||||||||||
Sbjct  718  CGGTTCAACCCCTTGGGTAGAAAGCCTTTCGGAGACCGTCCCTTCGGCAGACGCAACGGA  777

Query  802  ACCGAAGAAGGATCTCTCAGGCGTGATGGCCATCGTCGGCCCTATGGTAACCGAGGACGT  861
            ||||||||||||||||||||||||||||||||||||||||||||||||||||||||||||
Sbjct  778  ACCGAAGAAGGATCTCTCAGGCGTGATGGCCATCGTCGGCCCTATGGTAACCGAGGACGT  837

Query  862  TGGGGTGAGAATGAAAGTGAGGAGAAGGAGCATCCAACGACGGAAAACGTAATT---TCT  918
            |||||||||||||||||||||||||||||||||||||||||||||| |||||     |||
Sbjct  838  TGGGGTGAGAATGAAAGTGAGGAGAAGGAGCATCCAACGACGGAAAGCGTAACGACATCT  897

Query  919  TCACCAGCTGACGTGGTTGAGATCGCAGTCAATGAAGAAGACGTCAATGTGGTCGCCGAG  978
            |||||| |||||||||||||||||||||||||||||||||||||||||||||||||||||
Sbjct  898  TCACCACCTGACGTGGTTGAGATCGCAGTCAATGAAGAAGACGTCAATGTGGTCGCCGAG  957

Query  979  GTGTAG  984
            ||||||
Sbjct  958  GTGTAG  963
```

Figure S12 | The alignment of the *C4*a gene against the cDNA, *Sp0376*, shows a 98% identity with six gaps. NCBI BLAST analysis of *C4*a (Sbjct) from Cluster 4 is compared to *Sp0376* (query). The score, expect, identities, gaps, and strand are indicated at the top. The numbers at the end of each line indicate the nucleotide position for each sequence in the alignment. Areas of low-complexity in the sequence, as determined by the DustMasker program (Morgulis et al., 2006, see refence below), are indicated by lowercase letters. This low-complexity region encodes multiple histidines.

```
                         10        20        30        40        50        60        70        80        90       100
                ....|....|....|....|....|....|....|....|....|....|....|....|....|....|....|....|....|....|....|....|
A2a intron      GTAAGAAATCAAATTATTACTTGGTATTACTTGATAAGTGGCAAATATTAAGCCAAGAAAGGGCTCACAGGAGTATATT~~ATTATTTAATTTATCATAA
C4 intron       GTAAGAAATCAAATTATTACTCGGTATTACTTGATAAGTGCTAAATATAAAGCCAACGAAGGGTTCACAGAAGTAAATATGATTATTTCATAGTTTGTAA
B8a intron      GTAAGAAATCAAATT~TTACTCGGTATTACTTCATAAGTGCTAAATATAAAGCCAATGAAGGGCTCACAGAAGTAAATATGATTATTTCATAGTTTGTAA

                        110       120       130       140       150       160       170       180       190       200
                ....|....|....|....|....|....|....|....|....|....|....|....|....|....|....|....|....|....|....|....|
A2a intron      TATGTGTTTCTTACCTGTTTGTTACACAATACAAAAAATATTTCTTCTGTGGGCTT~~~~~~~~~~~~~~~~~~~~~~CAGCATTCCGTTTATTCGAATG
C4 intron       TATGCATTTTTAAATTGTTCGTTACAAATTATAA~~~~~~~~~~~~~~~~~~~~~~TTAATTTTATTTCTTAAACCTACACCAATCCGTTGG~TCGAATG
B8a intron      TATGCATTTTTAAATTGTTCGTTACACAATATAA~~~~~~~~~~~~~~~~~~~~~~TTAATTGTATTTATTAAGCCTACACCAATCCGTTTTCT~GAATG

                        210       220       230       240       250       260       270       280       290       300
                ....|....|....|....|....|....|....|....|....|....|....|....|....|....|....|....|....|....|....|....|
A2a intron      AAAAACACGTTTTA~~~TGTTTCTTTACCATCTGGTTTTCAAATTCATTTCGGTTGGAATTAGGCGTTTTGAATATCCAACCGCATGTATTCTGA~~~~~
C4 intron       ACAAAAGATGTGTATTATGTTTACGTTCAATCTGGTATTCAAATTTAATTCAGTCAGAATAGGGCATTTTGAAGGTCGAACCGCATGTA~~~~~~~~~~~
B8a intron      ACAAAGGATGTGTATTATGTTTACGTTCAATCTGGTAATCAAATTTAATTCAGTCAGAATTAGGCGTTTTGAATATCGAACCGCA~~~~~~~~~~TTTAC

                        310       320       330       340       350       360       370       380       390       400
                ....|....|....|....|....|....|....|....|....|....|....|....|....|....|....|....|....|....|....|....|
A2a intron      ~~~~TAGGGTTAAACTGAACAGGAACAGACATCGAAGAAGACAAGTGTTATCAATC~~ATTTGTCACCTAGCATATCCCAATGTAGAGCTAAATATCATA
C4 intron       ~~~~~~~~~~~~~~~~~~CA~~~~~~~~~~~~~GAAAAAGACAAGTGTTATCAATCTTATTTGTCACCGAGCATAAACCAATGTAGAGCCAAAGATAATA
B8a intron      GGATTAGGATTAAACATAAAGGGAAAAA~~~~~TATAAGAAGGCGAGTTACCATTCTTATTTGTCACCTGCTATAAACCAATGTAGAGCAAAATATAATA

                        410       420       430       440       450
                ....|....|....|....|....|....|....|....|....|....|
A2a intron      ATGCAGAATGTGA~~~~TTAATTAGTTCGATGATTTCATTACCCCCTACA
C4 intron       ACGCAGAATGTGA~~~~TTAATTAGTTCGATGATTTCATTACCCATTACA
B8a intron      ATGCAGAATGTGATTACTTAATTAATTCGATTATTTCATTAACCCTTACA
```

Figure S13 | The alignment of *C4* intron shows regions of sequence similarity with the introns from the *B8*a and the *A2*a genes. The gene names are listed to the left and the ruler above each alignment indicates the nucleotide position. Yellow highlights indicate positions of nucleotide mismatches. The ~ indicates the insertion of artificial gaps at the locations of putative indels. The alignment was done with ClustalW in BioEdit (ver 7.2.5).

```
             10        20        30        40        50        60        70        80        90       100
     ....|....|....|....|....|....|....|....|....|....|.....|....|....|....|....|....|....|....|....|....
D1f  ATGGAGGTGAAAGTGACACTGATCGTTGCCATTGTGGCTGCTCTTGCTATCTCAG^GTAAGAAATCAAATTATTACTCGGTATTCCTTGATAAGTGCTAA
D1h  .......................................................^............................................
D1d  ..~~~................................................G.^............................A...............
D1e  ..~~~..................................................^............................A...............
D1y  ..~~~..................................................^............................A...............
D1g  ..~~~..................................................^............................A...............
D1b  ..~~~.........................................T........^............................A...............

     100       110       120       130       140       150       160       170       180       190
     |....|....|....|....|....|....|....|....|....|....|....|....|....|....|....|....|....|....|....|....
D1f  ATATAGAGCCAACGAATAGCTCACAGAAGTATATATGATTATTTCATATTTTATAATATGCATTTCTAAATTGTTCGTTACACAATATAATTTATATTAT
D1h  ....................................................................................................
D1d  .....................................................................................A..............
D1e  ..................................................................................A.................
D1y  ..................................................................................A.................
D1g  ..................................................................................A.................
D1b  ..................................................................................A.................

     200       210       220       230       240       250       260       270       280       290
     |....|....|....|....|....|....|....|....|....|....|....|....|....|....|....|....|....|....|....|....
D1f  TTCTTAAGCCTACACCAATCCGTTGGTCGAATGGCAAAAGATAAGAATTATCTTTATGTTCAATCTGGTATTCAAGTTCAATTCAGTCAGAATTAGGCGT
D1h  ....................................................................................................
D1d  ....................................................................................................
D1e  ..........................G...G..........................A...........T..............................
D1y  ..........................G...G.....................................................................
D1g  ..........................G...G.....................................................................
D1b  ........................A.G...G.....................................................................

     300       310       320       330       340       350       360       370       380       390
     |....|....|....|....|....|....|....|....|....|....|....|....|....|....|....|....|....|....|....|....
D1f  TTTGAATATCGAACCGATAGGGTTAAACATAAAGGGAAAAAA~CATCAAAGAAGGCGAGTTACCATTCTTATTTGTCACCTGCCATAACCCAATGTAGAG
D1h  ..........................................~.........................................................
D1d  ..........................................~.........................................................
D1e  .............................C............~.........................................................
D1y  .............................C............~.........................................................
D1g  .............................C............A.........................................................
D1b  .............................C............~.........................................................
```

```
      400       410       420       430       440       450       460        470       480       490
      |....|....|....|....|....|....|....|....|....|....|....|....|....|. ...|....|....|....|....|....|....
D1f   CTAAAGATAATAATGCAGAATGTGATCACTTAATTAATTCGA~TTTTATTCTTCATTATCCCCTACA^GCTCACGCACAAAGAGATTACAATGAACGACGA
D1h   ..........................................~........................^.................................
D1d   ..G.......................................T..G....T................^.................................
D1e   ..........................................~~...T...................^............................T....
D1y   ..........................................~....T...................^............................T....
D1g   .....................................C....~....T...................^............................T....
D1b   ..........................................~....T...................^............................T....

      500       510       520       530       540       550       560       570       580       590
      |....|....|....|....|....|....|....|....|....|....|....|....|....|....|....|....|....|....|....|....
D1f   GGAAATGAGAATGGCAGAGAGAGAGGACAAGGTCGCTTTGGAGGAAGGCCTGGTGGAATGCAGACGGGTGGACCGAGGCAAGATGGTGGACCAATGGGTG
D1h   ....................................................................................................
D1d   ..........................G.....................................T.......T.T.........................
D1e   ......A...........................................G.............T.......T...........................
D1y   ......A...........................................G.............T.......T...........................
D1g   ......A...........................................G.............T.A.....T...........................
D1b   ......A...........................................G.............T.......T...........................

      600       610       620       630       640       650       660       670       680       690
      |....|....|....|....|....|....|....|....|....|....|....|....|....|....|....|....|....|....|....|....
D1f   GAAGAAGGTTCGATGGACCTGACTCTGGTGCCCCACAAATGGATGGACGGAGACAAGATGGTGGACCAATGGGTGGAAGGAGGTTCGATGGACCTGGATT
D1h   ....................................................................................................
D1d   ...A................................................................................................
D1e   ....................................................................................................
D1y   .G..................................................................................................
D1g   .G..................................................................................................
D1b   .G..................................................................................................

      700       710       720       730       740       750       760       770       780       790
      |....|....|....|....|....|....|....|....|....|....|....|....|....|....|....|....|....|....|....|....
D1f   TGGTGCCCCGGAGATGGATGGACGGAGACAAAATGGCGGTCCGATGGGTGGAAGGAGATTCGACGGACCTGGATTTGGTGGCTCCAGACCAGTTGGTGCT
D1h   ....................................................................................................
D1d   ....................................................................................................
D1e   ............................................................................................A.......
D1y   ............................................................................................A.......
D1g   ............................................................................................A.......
D1b   ............................................................................................A.......
```

```
      800       810       820       830       840       850       860       870       880       890
      |....|....|....|....|....|....|....|....|....|....|....|....|....|....|....|....|....|....|....|....
D1f   GGAGGAAGACCTGTCTTCGGCCAAGGAGGCAGGCGTGGTGATGGAGAAGAAGAAACTGATGCTGCCCAACAAATTGGTGATGGTCTAGGAGGGCCCGGTC
D1h   ....................................................................................................
D1d   ............T.......................................................................................
D1e   ............T.................................................................................G.....
D1y   ............T.......................................................................................
D1g   ............T.......................................................................................
D1b   ............T.......................................................................................

      900       910       920       930       940       950       960       970       980       990
      |....|....|....|....|....|....|....|....|....|....|....|....|....|....|....|....|....|....|....|....
D1f   AGTTTGATGGTCCTGGACGTCGTCATCATGGTCACCGTCAAGGTCATCCTCAGGACCAAGCAGAGGAACAACCGTTTGGTCAGCGCAACGAAAGCAGCGA
D1h   ....................................................................................................
D1d   .........................................................................................A..........
D1e   ............A................................CA..........G.C........................................
D1y   ....................................................................................................
D1g   ....................................................................................................
D1b   ....................................................................................................

      1000      1010      1020      1030      1040      1050      1060      1070      1080      1090
      |....|....|....|....|....|....|....|....|....|....|....|....|....|....|....|....|....|....|....|....
D1f   GGAGGATGGCCGTCCTCACCCTCACCACCATCGCCACCATGGCCACCACCACCGTCATCATAACCACACAGAAGGTCACCAAGGTCATAATGAGACGGGA
D1h   ....................................................................................................
D1d   ..................................G.................................................................
D1e   ....................................................................................................
D1y   ....................................................................................................
D1g   ............................................G..................................A....................
D1b   ............................................G.......................................................

      1100      1110      1120      1130      1140      1150      1160      1170      1180      1190
      |....|....|....|....|....|....|....|....|....|....|....|....|....|....|....|....|....|....|....|....
D1f   GATCAAGATCAGGACAAACTACATGATACAAGGCCCTTCCGGTACAACCACTTCGGTAGAAAGCCTTTCGGAGACCGTCCCTTCGGCAGACGCAACCATA
D1h   ....................................................................................................
D1d   ....................................................................................................
D1e   ......................................................................................A.............
D1y   ....................................................................................................
D1g   ....................................................................................................
D1b   ....................................................................................................
```

```
      1200      1210      1220      1230      1240      1250      1260      1270      1280      1290
      |....|....|....|....|....|....|....|....|....|....|....|....|....|....|....|....|....|....|....|....
D1f   CAGAAGGTCACCAGGGTCATAATGAGACGGGAGACCACCCCCATCGTCATCACAACAAGACCAGAGATGGAGATCAGGACAGACCAATGTTCGAGATGAG
D1h   ....................................................................................................
D1d   ...............................................................................................TC...
D1e   ....................................................................................................
D1y   ....................................................................................................
D1g   ..................................A.................................................................
D1b   ....................................................................................................

      1300      1310      1320      1330      1340      1350      1360      1370      1380      1390
      |....|....|....|....|....|....|....|....|....|....|....|....|....|....|....|....|....|....|....|....
D1f   GCCCTTCCGGTTCAACCCCTTGGGTAGAAAGCCTTTCGGAGACCGTCCCTTCGGCAGACGCAACGGAACCGAGGAAGGATCTCCCAGGCGTGATGGCCAA
D1h   ....................................................................................................
D1d   .....................C.........................T........................A.........T................C
D1e   .....................C...................G...........A..................A..........................T
D1y   .....................CC..................G...........A..................A..........................T
D1g   ...T.................C...................G...........A..................A..........................T
D1b   ...T.................C...................G...........A..................A..........................T

      1400      1410      1420      1430      1440      1450      1460      1470      1480      1490
      |....|....|....|....|....|....|....|....|....|....|....|....|....|....|....|....|....|....|....|....
D1f   CGTCGGCCCTATGGTAACCGAGGACGATGAGGTGAGAATGAAAGTGAGGAGAAGGAGCATCCAACGACGGAAAGCGTAACGACATCTTCACCACCTGAAG
D1h   ....................................................................................................
D1d   .............................G......................................................................
D1e   .............................G................................................................T.....
D1y   .............................G......................................................................
D1g   .............................G.......G..............................................................
D1b   ..........................T..G......................................................................

      1500      1510      1520      1530      1540
      |....|....|....|....|....|....|....|....|....|.
D1f   TGGTCGCAATCAATGAAGAAGACATCAATGTGGTCGCCGAGGTGTAG
D1h   ...............................................
D1d   ...............................................
D1e   ...............................................
D1y   ...............................................
D1g   ...............................................
D1b   ...............................................
```

Figure S14 | The alignment of the *D1* genes shows nucleotide variations that are present throughout the sequences. The gene names are listed to the left and nucleotide position is indicated above. All sequences are compared to *D1*f and the dots in the sequences below indicate matching nucleotides. The ~ indicates gaps in the alignment where the sequences do not match and artificial gaps are inserted. The ^ indicates the start and end of the intron. The alignment was done using ClustalW in BioEdit (ver. 7.2.5).

```
              10        20        30        40        50         60        70        80        90
E2     ATGGAGGTGAAAGTGACACTGATCGTTGCCATTGTGGCTGCTCTTGCTATCTCGG^GTAAGAAATCAAATTATTACTCGGTATTCCTTCATAAGTGTTAA
E2a    ......................................................."^"............................................
E2b    ...~~~.................................................^........................................G..T
01     ...~~~....................................T............^.............................T..........G..T

            110       120       130       140       150       160       170       180       190
E2     ATATTAAGCCAACAAATGACTCACAGT~AGCATATTATTATTTTGATTTATTATAAAAGGTGTTTCTAACTTGTTTGTAACACAATAAAAA~TAACATTT
E2a    ...........................~..T............................................................~........
E2b    ..T~~G..........ATTACTCACA.A..T.....G...................T.T...................G............A........
01     ..T~~~~~~~~~~~~~~~~~~~~~~~~~~~~~~~~~~~~~~~~~~~~~~~~~~~~~~~~~~~~~~G...........G............A........

       200       210       220       230       240       250       260       270       280       290
E2     ATTCTTCGGCCTGCAGCATTCTGTTTTTTTTTTATGAAAAAAATGTTTCATGGTTCTTTACAATCTGGTTTTCATATTCAATTCGTTTAGAATTAGGCGT
E2a    ................................~...................................................................
E2b    .....G.................G.......A~.....................A...................A..........G...A..........
01     .....G.........................~~.....................A...................A..........G...A..........

       300       310       320       330       340       350       360       370       380       390
E2     TTTGAATATCCAACCGCATGCATGCATTCTGGTAGAGTTAAACATAACGGGACCAGGCATCGAAGAAGACAAATCTCCTAGCATAAACCAATGTAGAGCT
E2a    ........................................................A...........................................
E2b    ........................................................A...........................................
01     ........................................................A...........................................

       400       410       420       430       440       450       460       470       480       490
E2     AAAGATAATAATGCAGAATGTGATTAATT~~~TTATTAATAAATTCGACTATTTCATTATCCCAACA^GCTCACGCACAAAGAGATTTCAATGAACGACGA
E2a    .............................~~~...................................^.................................
E2b    .............................~~~...................................^..........G......................
01     .............................ATT.....................C.............^..........G......................

       500       510       520       530       540       550       560       570       580       590
E2     GGAAAGGAGAATGACACAGAGAGAGGACAAGGTGGCTTTGGAGGAAGGCCTGGTGGAATGCAGATGGGTAGTCCTAGGCAAGATGGTGGACAAATGGGTG
E2a    .....................................................................G.....................C........
E2b    .............G..................................................C.......................~~~~~~~~~~~~
01     .............G..G...............................................C..........................C........
```

```
      600       610       620       630       640       650       660       670       680       690
      |....|....|....|....|....|....|....|....|....|....|....|....|....|....|....|....|....|....|....|....
E2    GAAGGAGGTTCGATGGACCTGAATCTGGTGCCCCACAAATGGAAGGACGCAGACAAAATGGCGGTCCGATGGGTGGTAGGAGATTCGACGGACCTCGATT
E2a   ....................................................................................................
E2b   ...........................................T........................................................
01    ...T.......................................T...................................................G....

      700       710       720       730       740       750       760       770       780       790
      |....|....|....|....|....|....|....|....|....|....|....|....|....|....|....|....|....|....|....|....
E2    TGGTGGCTCCAGACCAGATGGTGCTGGAGGAAGACCTTTCTTCGGCCAAGGAGGCAGGCGTGGTGATGGAGAAGAAGAAACTGATGCTGCCCAACAAATT
E2a   ..............................G.....................................................................
E2b   ..............................G.....................................................................
01    ....................................................................................................

      800       810       820       830       840       850       860       870       880       890
      |....|....|....|....|....|....|....|....|....|....|....|....|....|....|....|....|....|....|....|....
E2    GGTGATGGTCTAGGAGGGCGCGGTCAGTTTGATGGTCATGGACGTGGACATCATGGTCACCGTCAAGGTCCTCCTCAGGACCGACCAGAGGAACAACCGT
E2a   ....................................................................................................
E2b   ..................TC.................C.A.......T....................................................
01    ...................C.................C.......C.T.........A~~~~~~~~~~~~~~~~~~~~~~~~~~~~~~~~~~~~~~~~~~

      900       910       920       930       940       950       960       970       980       990
      |....|....|....|....|....|....|....|....|....|....|....|....|....|....|....|....|....|....|....|....
E2    TCGGTCAGCGCAACGAAAGCAGCGATGAGGATGGCCGTCCTCACCCTCGCCACCATGGCCGCCACCACCAGCATCATCATCGCAACCACACAGAAGGTCA
E2a   ....................................................................................................
E2b   ....................................................................................................
01    ~~~~~~~~~~~~~~~~~~~~~~~~~~~~~~~~~~~~~~~~~~~~~~~~.AA.G.C.TT.G.AG...GT.CCTTCGGCAGA........T...........

      1000      1010      1020      1030      1040      1050      1060      1070      1080      1090
      |....|....|....|....|....|....|....|....|....|....|....|....|....|....|....|....|....|....|....|....
E2    CCAAGGTCACAATGAGACAGGAGATCACCCCCACCGTCATCACAACAAGACCGGAGATGGAGATCAGGACAGACCAATGTTTGAGATGAGGCCCTTCCGG
E2a   ....................................................................................................
E2b   ....................................................................................................
01    ...G.....T........G.....C........T..................A............................C..............T...

      100       1110      1120      1130      1140      1150      1160      1170      1180      1190
      |....|....|....|....|....|....|....|....|....|....|....|....|....|....|....|....|....|....|....|....
E2    TTCAACCCGTTCGGTAGAAAGCCTTTCGGAGACCGTCCCTTCGGCAGACGCAACGGAACCGAGGAAGGATCTCCCAGGCGTGATGGCCAACGTCGGCCCT
E2a   ........C..................................A..................A.....................................
E2b   ........C...........................................................................................
01    ........C..........................................................................................C
```

```
       1200      1210      1220      1230      1240      1250      1260      1270      1280      1290
        |....|....|....|....|....|....|....|....|....|....|....|....|....|....|....|....|....|....|....|....
E2      ATGGTAACCGAGGACGTTGGGGTGAGAATGAAAGTGAGGAGAAGGAGCATCCAACGACGGAAAGCGTAACGACATCTTCACCACCTTAAGTGGTCGAGAT
E2a     ....................................................................................................
E2b     ....................................................................................................
01      ......................................................................................G.C...........

       1300      1310      1320      1330      1340
        |....|....|....|....|....|....|....|....|...
E2      CGCAGTCAATGAAGAAGACATCAATGTGGTCGCCGAGGTATAG
E2a     ...........................................
E2b     ...GA.........~~~~~~~~~~~~~~~..........G...
01      ...GA.......T.~~~~~~~~~~~~~~~..........G...
```

Figure S15 | The alignment of the *E2* genes and *01* gene shows sequence similarity among these genes. The names of the genes are listed to the left and above each alignment is a ruler indicating the nucleotide position. The *E2* gene in Cluster 1 is used for the comparison to the other sequences. Dots represent a matching nucleotide to the top sequence. The ~ indicates the insertion of artificial gaps in the alignment where the sequences do not match. The alignment includes sequence from both exons and the ^ indicates the start and end of the intron. The highlighted regions indicate large indels in the intron and in exon 2 of the *01* gene. The alignment was done with ClustalW in BioEdit (ver 7.2.5).

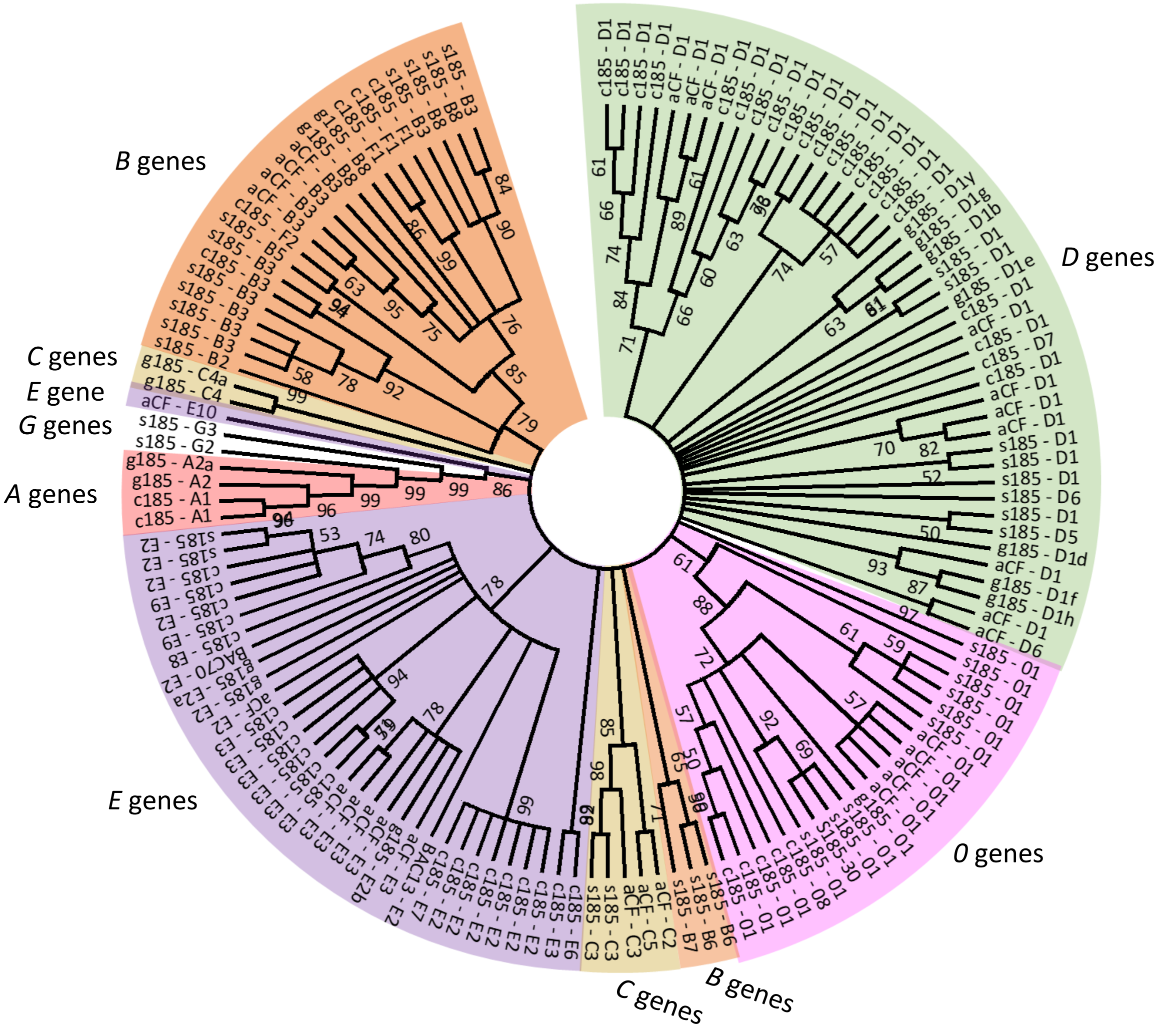

Figure S16 | A maximum likelihood phylogenetic tree of exon 2 shows a loss of tree structure with the addition of 121 *SpTrf* genes. The phylogenetic tree was used to evaluate the 17 *SpTrf* genes from the clusters in the BAC insert sequences (labeled g185) with 121 additional *SpTrf* exon 2 sequences (labeled s185, c185, and aCF) from (Buckley and Smith, Ref. 19 in the main paper). Highlighted regions of the same color indicate genes with the same element pattern based on the cDNA alignment (Figure 3; Terwilliger et al., Ref. 12 in the main paper). The tree was constructed in MEGA7 using an alignment generated in PRANK. Bootstrap values from 500 iterations are indicated at most nodes and those below 50 were collapsed. The accession numbers for the sequences used to generate this tree can be found in the materials and methods.

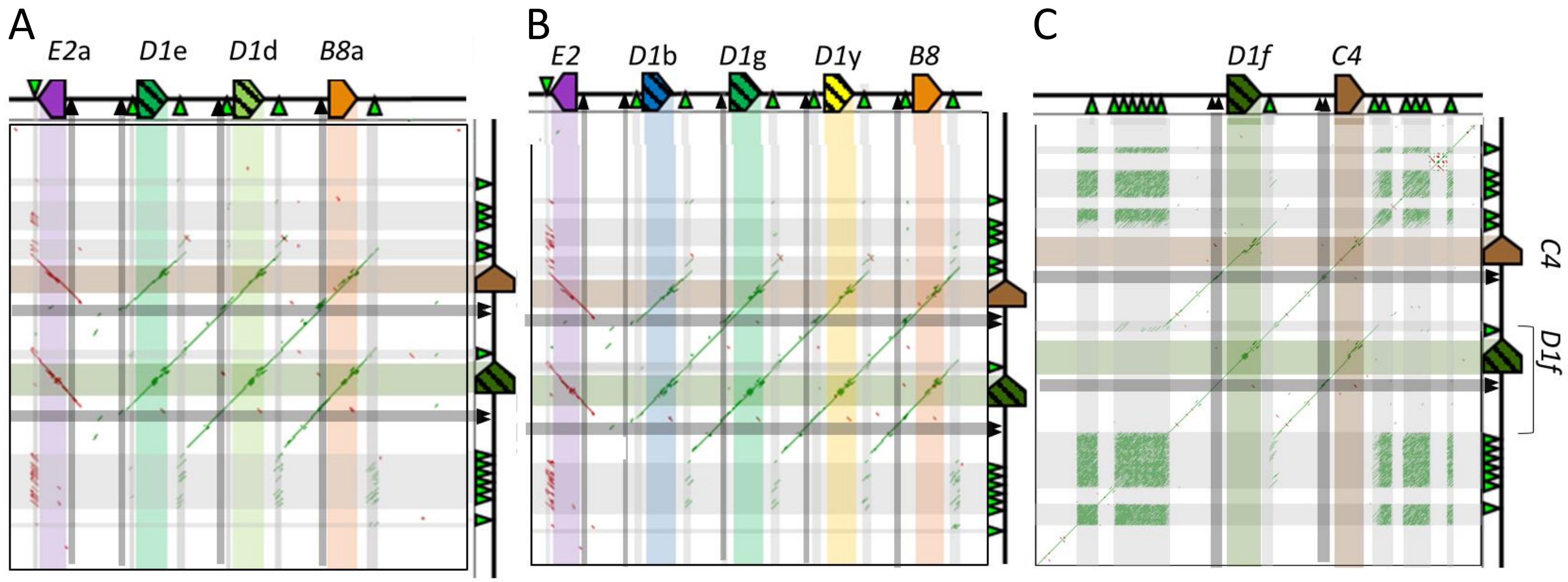

Figure S17 | Dot plots of Cluster 3 *vs.* other *SpTrf* Clusters shows the GA STRs as edges of gene duplications. Representative clusters are located to the right and top of the dot plots in which the polygons indicate genes and directionality, the green triangles represent GA STRs and black triangles represent GAT STRs. The central green diagonal in **(C)** indicates the main alignment of Cluster 3 *vs.* Cluster 3. Diagonal lines in green outside of and parallel to the central diagonal indicate repeat regions in the same orientation. Red diagonal lines that are perpendicular to the central diagonal indicate regions of sequence similarity that are in opposite orientation. Highlighted horizontal and vertical areas in each plot (shown in multiple colors that match to the genes above or to the right) are added to aid in comparisons among the clusters. Dark green areas in both horizontal and vertical orientations indicate the locations of GA STRs while dark gray lines indicate the locations of the GAT STRs. **(A)** Cluster 2 *vs.* Cluster 3. **(B)** Cluster 1 *vs.* Cluster 3. **(C)** Cluster 3 *vs.* Cluster 3. Dot plots were done in YASS (http://bioinfo.lifl.fr/yass/index.php) using standard parameters (scoring matrix = +5, -4, -3 -4: composition bias correction: gap costs = -16, -4: e-value threshold = 10: X-drop threshold = 30).

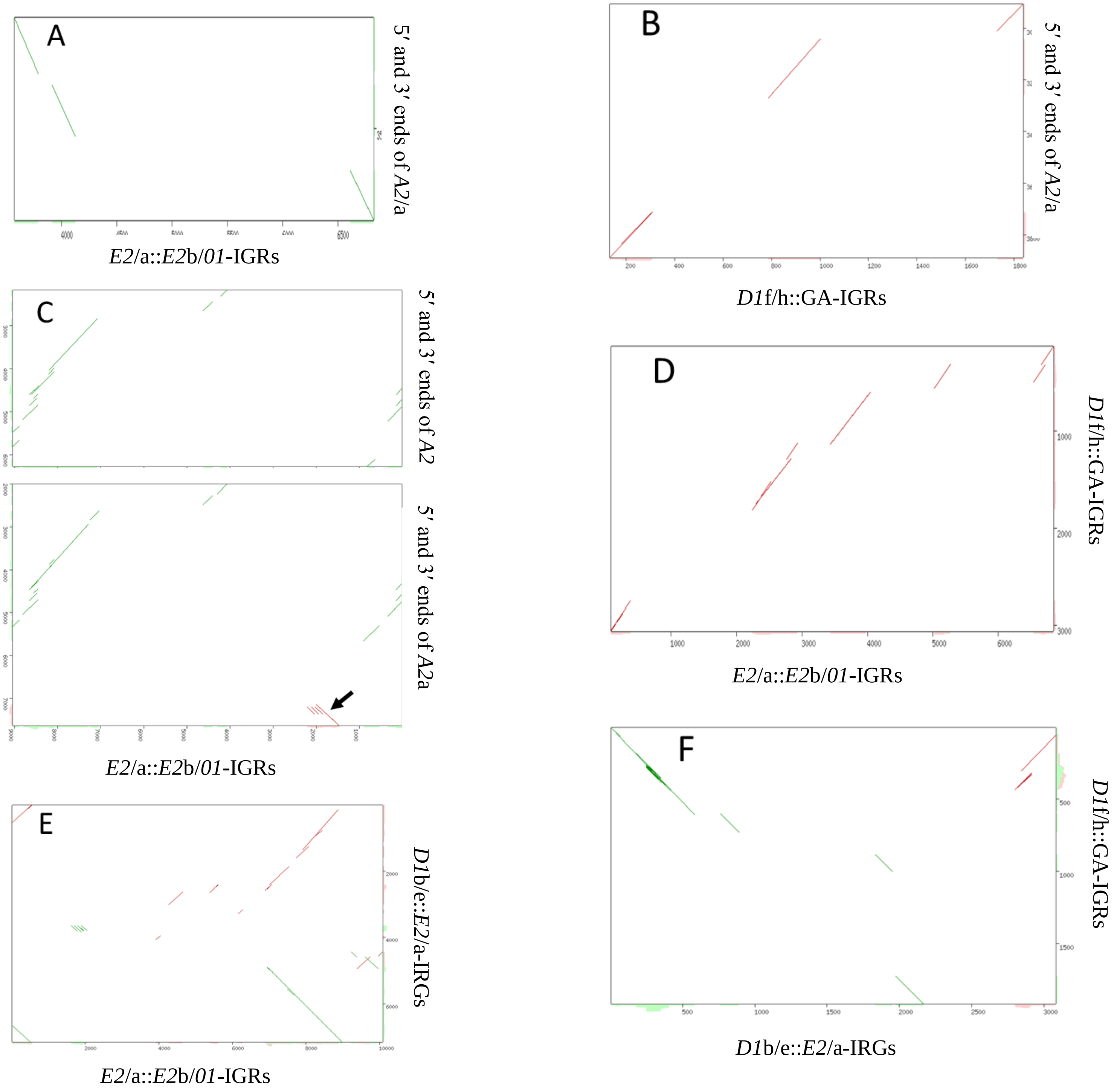
A
5′ and 3′ ends of *A2*/a
E2/a::*E2*b/*01*-IGRs
B
5′ and 3′ ends of *A2*/a
*D1*f/h::GA-IGRs
C
5′ and 3′ ends of *A2*
5′ and 3′ ends of *A2*a
E2/a::*E2*b/*01*-IGRs
D
*D1*f/h::GA-IGRs
E2/a::*E2*b/*01*-IGRs
E
*D1*b/e::*E2*/a-IRGs
E2/a::*E2*b/*01*-IGRs
F
*D1*f/h::GA-IGRs
*D1*b/e::*E2*/a-IRGs

Figure S18 | Comparisons among IGRs identify regions of sequence similarity. The diagonal lines in each dotplot indicate regions of similar sequence. The green diagonals indicate regions in the same orientation, whereas the red diagonals indicate regions in opposite orientations. **(A)** The 5′ and 3′ ends of the *A2*/a gene *vs*. *E2*/a::*E2*b/*01*-IGRs. **(B)** The 5′ and 3′ ends of the *A2*/a gene *vs*. *D1*f/h::GA-IGRs. **(C)** The *E2*/a::*E2*b/*01*-IGRs *vs*. *D1*f/h::GA-IGRs. **(D)** The *E2*/a::*E2*b/*01*-IGRs *vs*. *D1*b/e::*E2/*a-IGRs. **(E)** The *D1*b/e::*E2/*a-IGRs *vs*. *D1*f/h::GA-IGRs. **(F)** The 5′ and 3′ ends of the *A2* and *A2*a genes *vs. E2*/a::*E2*b/*01*-IGRs. The black arrow in **(C)** indicates variation between the two sequences showing an inverted region that includes repeats. YASS genomic similarity search tool (http://bioinfo.lifl.fr/yass/index.php) was used to identify sequence similarities with the following parameters (scoring matrix = +5, -4, -3 -4: composition bias correction: gap costs = -16, -4: e-value threshold = e^{-20} : X-drop threshold = 30).

Supplemental Figure Reference

Morgulis A, Gertz EM, Schäffer AA, Agarwala R. A fast and symmetric DUST implementation to mask low-complexity DNA sequences. *J Comp Biol* (2006) 13(5):1028–1040. doi: 10.1089/cmb.2006.13.1028
