# Supplementary material for: Sequence Diversity, Locus Structure, and Evolutionary History of the SpTransformer Genes in the Sea Urchin Genome
Source: Front Immunol. 2021 Nov 15;12:744783. doi: 10.3389/fimmu.2021.744783 (PMC8634487; doi:10.3389/fimmu.2021.744783)
Supplement: Supplementary file 2 [file DataSheet_2.pdf]

Supplementary Tables

Table S1 | Primers used to identify allelic BACs and verify BAC insert assembly

Name	Sequence	T_m (ºC)	Annealing temperature (ºC)
**Cluster 10k 1 R**	TGTTGAGAAGAAGCGAAGCGAG	56	60*
**Cluster 10k 1 F**	GTAGACCTGCACTATG	56	60*
**GA1F**	TCCATAAGAGAGTTCTATTTCC	58	60*
**GA1R**	ATTTACTCAGAGGTACCCA	59	60*
**GA2F**	TTTGAGTTAACGCCCTTC	59	60*
**GA2R**	CCAGCTGCATAAGGAAA	59	60*
**GA3F**	TACAAACTTCCTACTTCGTG	59	60*
**GA3R**	AATCTTTCATCTGTGGTAGG	59	60*
**R1**	TCTSCATTCCAYCMGGCC	64	56
**F2**	AAGMGATTWCAATGAACKRCGA	58	55
**F5**	GGAACYGARGAMGGATCTC	59	56
**F6**	GAAGAAGAAACTGATGCTGCC	64	55
**R9**	CTTHARGTGGTGAARATGTCG	59	55
**5′UTR**	YTDTAGCATCGCAGAKACCT	60	55
**3′UTR**	WAATTCTACACCTCRGCGAC	61	55

*The annealing temperature is based on the PrimeStar GLX Protocol as recommended by the manufacturer (Takara Bio).

Table S2 | Most single nucleotide changes among genes of the same element pattern result is nonsynonymous changes in the amino acids of the deduced proteins

Deduced proteins compared		1aa[1]	2aa[2]	Change[3]	Δcharge[4]	ΔpI[5]	Number[6]
A2	A2a	Q	R	*	PU / EC+	*	1
A2	A2a	S	R	*	PU / EC+	*	2
A2	A2a	R	L	*	EC+ / H	*	3
A2	A2a	G	S	*	SC / PU		4
A2	A2a	R	G	*	EC+ / SC		5
A2	A2a	Q	H	*	PU / EC+	*	6
A2	A2a	T	S		PU / PU		7
A2	A2a	N	T		PU / PU		8
A2	A2a	E	G	*	EC- / SC		9
A2	A2a	L	F		H / H		10
B8	B8a	S	A	*	PU / H		1
B8	B8a	Q	H	*	PU / EC+	*	2
B8	B8a	G	D	*	SC / EC-	*	3
B8	B8a	R	G	*	EC+ / SC	*	4
B8	B8a	V	I		H / H		5
D1f/h	D1d	T	M	*	PU / H		1
D1f/h	D1d	P	S	*	SC / PU		2
D1f/h	D1d	R	K		EC+ / EC+		3
D1f/h	D1d	V	F		H / H		4
D1f/h	D1d	E	K	*	EC- / EC+	*	5
D1f/h	D1d	H	D	*	EC+ / EC-	*	6
D1f/h	D1d	M	S	*	H / PU		7
D1f/h	D1d	L	F		H / H		8
D1f/h	D1d	P	L		SC / H		9
D1f/h	D1d	P	S	*	SC / PU		10
D1f/h	D1d	Q	H	*	PU / EC+	*	11

D1f/h	D1d	stop	W	*	STOP / H		12
D1f/h	D1e	R	L	*	EC+ / H	*	1
D1f/h	D1e	E	K	*	EC- / EC+	*	2
D1f/h	D1e	T	M	*	PU / H		3
D1f/h	D1e	P	S	*	SC / PU		4
D1f/h	D1e	V	D	*	H - / EC-	*	5
D1f/h	D1e	V	F		H / H		6
D1f/h	D1e	P	R	*	SC / EC+		7
D1f/h	D1e	P	H	*	SC / EC+	*	8
D1f/h	D1e	H	P	*	H / SC		9
D1f/h	D1e	Q	R	*	PU / EC+	*	10
D1f/h	D1e	A	P		H / SC		11
D1f/h	D1e	L	F		H / H		12
D1f/h	D1e	D	G	*	EC- / SC		13
D1f/h	D1e	G	D	*	SC / EC-	*	14
D1f/h	D1e	Q	H	*	PU / EC+	*	15
D1f/h	D1e	stop	W	*	STOP / H		16
D1f/h	D1y	R	L	*	EC+ / H		1
D1f/h	D1y	E	K	*	EC- / EC+	*	2
D1f/h	D1y	T	M	*	PU / H		3
D1f/h	D1y	P	S	*	SC / PU		4
D1f/h	D1y	V	D	*	H / EC-		5
D1f/h	D1y	V	F		H / H		6
D1f/h	D1y	L	F		H / H		7
D1f/h	D1y	G	R	*	SC / EC+		8
D1f/h	D1y	D	G	*	EC- / SC	*	9
D1f/h	D1y	G	D	*	SC / EC-	*	10
D1f/h	D1y	Q	H	*	PU / EC+	*	11
D1f/h	D1y	stop	W	*	STOP / H		12
D1f/h	D1g	R	L	*	EC+ / H	*	1
D1f/h	D1g	E	K	*	EC- / EC+	*	2

D1f/h	D1g	T	M	*	PU / H		3
D1f/h	D1g	G	S	*	SC / PU		4
D1f/h	D1g	P	S	*	SC / PU		5
D1f/h	D1g	V	D	*	H / EC-	*	6
D1f/h	D1g	V	F		H / H		7
D1f/h	D1g	H	R		EC+ / EC+		8
D1f/h	D1g	Q	K	*	PU / EC+	*	9
D1f/h	D1g	D	E		EC- / EC-		10
D1f/h	D1g	L	F		H / H		11
D1f/h	D1g	D	G	*	EC- / SC	*	12
D1f/h	D1g	G	D	*	SC / EC-	*	13
D1f/h	D1g	Q	H	*	PU / EC+	*	14
D1f/h	D1g	stop	W	*	STOP / H		15
D1f/h	D1b	R	L	*	EC+ / H	*	1
D1f/h	D1b	E	K	*	EC- / EC+	*	2
D1f/h	D1b	T	M	*	PU / H		3
D1f/h	D1b	P	S	*	SC / PU		4
D1f/h	D1b	V	D	*	H / EC-	*	5
D1f/h	D1b	V	F		H / H		6
D1f/h	D1b	H	R		EC+ / EC+		7
D1f/h	D1b	L	F		H / H		8
D1f/h	D1b	D	G	*	EC- / SC	*	9
D1f/h	D1b	G	D	*	SC / EC-	*	10
D1f/h	D1b	Q	H	*	PU / EC+	*	11
D1f/h	D1b	stop	W	*	STOP / H		12
D1d	D1e	R	L	*	EC+ / H		1
D1d	D1e	E	K	*	EC- / EC+	*	2
D1d	D1e	K	R		EC+ / EC+		3
D1d	D1e	V	D	*	H / EC-		4
D1d	D1e	P	R	*	SC / EC+	*	5
D1d	D1e	P	H	*	SC / EC+	*	6

D1d	D1e	H	P	*	EC+ / SC	*	7
D1d	D1e	Q	R	*	PU / EC+	*	8
D1d	D1e	A	P		H / SC		9
D1d	D1e	K	E	*	EC+ / EC-	*	10
D1d	D1e	D	H	*	EC- / EC+	*	11
D1d	D1e	S	M	*	PU / H		12
D1d	D1e	D	G	*	EC- / SC	*	13
D1d	D1e	L	P		H / SC		14
D1d	D1e	G	D	*	SC / EC-	*	15
D1d	D1e	S	P	*	PU / SC		16
D1d	D1e	P	L		SC / H		17
D1d	D1y	R	L	*	EC+ / H		1
D1d	D1y	E	K	*	EC- / EC+	*	2
D1d	D1y	K	R		EC+ / EC+	*	3
D1d	D1y	V	D	*	H / EC-		4
D1d	D1y	K	E	*	EC+ / EC-	*	5
D1d	D1y	D	H	*	EC- / EC+	*	6
D1d	D1y	S	M	*	PU / H		7
D1d	D1y	G	R	*	SC / EC+	*	8
D1d	D1y	D	G	*	EC- / SC	*	9
D1d	D1y	L	P		H / SC		10
D1d	D1y	G	D	*	SC / EC-	*	11
D1d	D1y	S	P	*	PU / SC		12
D1d	D1g	R	L	*	EC+ / H		1
D1d	D1g	E	K	*	EC- / EC+	*	2
D1d	D1g	G	S	*	SC / PU		3
D1d	D1g	K	R		EC+ / EC+		4
D1d	D1g	V	D	*	H / EC-	*	5
D1d	D1g	K	E	*	EC+ / EC-	*	6
D1d	D1g	D	H	*	EC- / EC+	*	7
D1d	D1g	H	R		EC+ / EC+	*	8

D1d	D1g	Q	K	*	PU / EC+		9
D1d	D1g	D	E		EC- / EC-		10
D1d	D1g	S	M	*	PU / H		11
D1d	D1g	D	G	*	EC- / SC	*	12
D1d	D1g	L	P		H / SC		13
D1d	D1g	G	D	*	SC / EC-	*	14
D1d	D1g	S	P	*	PU / SC		15
D1d	D1g	N	S		PU / PU		16
D1d	D1b	R	L	*	EC+ / H	*	1
D1d	D1b	E	K	*	EC- / EC+	*	2
D1d	D1b	K	R		EC+ / EC+		3
D1d	D1b	V	D	*	H / EC-	*	4
D1d	D1b	K	E	*	EC+ / EC-	*	5
D1d	D1b	D	H	*	EC- / EC+	*	6
D1d	D1b	H	R		EC+ / EC+		7
D1d	D1b	S	M	*	PU / H		8
D1d	D1b	D	G	*	EC- / SC	*	9
D1d	D1b	L	P		H / SC		10
D1d	D1b	G	D	*	SC / EC-	*	11
D1d	D1b	S	P	*	PU / SC		12
D1e	D1y	R	P	*	EC+ / SC	*	1
D1e	D1y	H	P	*	EC+ / SC	*	2
D1e	D1y	P	H	*	SC / EC+	*	3
D1e	D1y	R	Q	*	EC+ / PU	*	4
D1e	D1y	P	A		SC / H		5
D1e	D1y	G	R	*	SC / EC+	*	6
D1e	D1y	L	P		H / SC		7
D1e	D1g	G	S	*	SC / PU		1
D1e	D1g	R	P	*	EC+ / SC	*	2
D1e	D1g	H	P	*	EC+ / SC	*	3
D1e	D1g	P	H	*	SC / EC+	*	4

D1e	D1g	R	Q	*	EC+ / PU	*	5
D1e	D1g	P	A		SC / H		6
D1e	D1g	H	R		EC+ / EC+	*	7
D1e	D1g	Q	K	*	PU / EC+	*	8
D1e	D1g	D	E		EC- / EC-		9
D1e	D1g	N	S		PU / PU		10
D1e	D1g	L	P		H / SC		11
D1e	D1b	R	P	*	EC+ / SC	*	1
D1e	D1b	H	P	*	EC+ / SC	*	2
D1e	D1b	P	H	*	SC / EC+	*	3
D1e	D1b	R	Q	*	EC+ / PU	*	4
D1e	D1b	P	A		SC / H		5
D1e	D1b	H	R		EC+ / EC+		6
D1e	D1b	L	P		H / SC		7
D1y	D1g	G	S	*	SC / PU		1
D1y	D1g	H	R		EC+ / EC+		2
D1y	D1g	Q	K	*	PU / EC+	*	3
D1y	D1g	D	E		EC- / EC-		4
D1y	D1g	R	G	*	EC+ / SC	*	5
D1y	D1g	N	S		PU / PU		6
D1y	D1b	H	R		EC+ / EC+		1
D1y	D1b	R	G	*	EC+ / SC	*	2
D1g	D1b	S	G	*	PU / SC		1
D1g	D1b	K	Q	*	EC+ / PU	*	2
D1g	D1b	E	D		EC- / EC-		3
D1g	D1b	S	N		PU / PU		4
E2	E2a	S	G	*	PU / SC		1
E2	E2a	Q	P	*	PU / SC		2
E2	E2a	G	D	*	SC / EC-		3
E2	E2b	Q	R	*	PU / EC+		1
E2	E2b	D	G	*	EC- / SC	*	2

E2	E2b	M	T	*	H / PU		3
E2	E2b	E	D		EC- / EC-		4
E2	E2b	R	S	*	EC+ / PU	*	5
E2	E2b	H	P	*	EC+ / SC	*	6
E2	E2b	G	R	*	SC / EC+	*	7
E2a	E2b	Q	R	*	PU / EC+	*	1
E2a	E2b	D	G	*	EC- / SC		2
E2a	E2b	M	T	*	H / PU		3
E2a	E2b	G	S	*	SC / PU		4
E2a	E2b	E	D		EC- / EC-		5
E2a	E2b	R	S	*	EC+ / PU	*	6
E2a	E2b	H	P	*	EC+ / SC	*	7
E2a	E2b	G	R	*	SC / EC+	*	8
E2a	E2b	D	G	*	EC- / SC	*	9

[1]Amino acid associated with the protein listed in the first column.

[2]Amino acid associated with the protein listed in the second column.

[3]The asterisk in this column indicates a difference in amino acid properties between the two proteins that are compared.

[4]PU, polar uncharged; SC, special cases; EC+, electrically charged positive; EC-, electrically charged negative; H, hydrophobic

[5]The asterisk in this column indicates a difference in the pI of the amino acid R group between the two proteins that are compared.

Table S3 | Percent identity for each gene region shows similarity among genes with the same element pattern*

Genes Compared		5'FR	Exon 1	Intron	Exon 2	3'FR
A2	*A2*a	81	95	88	98	86
A2	*B8*	67	95	70	42	55
A2	*B8*a	68	95	69	42	58
A2	*C4*	69	95	63	46	54
A2	*D1*y	72	93	70	55	51
A2	*D1*g	72	93	70	55	52
A2	*D1*b	72	91	70	55	52
A2	*D1*d	70	95	70	55	58
A2	*D1*e	72	93	70	55	54
A2	*D1*f	83	98	70	55	55
A2	*E2*	71	100	54	45	57
A2	*E2*a	70	100	55	45	57
A2	*E2*b	74	95	54	43	58
A2	*01*	74	93	42	48	57
*A2*a	*B8*	69	96	63	42	50
*A2*a	*B8*a	70	96	62	43	55
*A2*a	*C4*	71	96	61	47	52
*A2*a	*D1*y	72	87	64	56	49
*A2*a	*D1*g	72	87	64	56	50
*A2*a	*D1*b	72	85	64	56	50
*A2*a	*D1*d	67	89	64	56	56
*A2*a	*D1*e	72	87	64	56	52
*A2*a	*D1*f	77	93	64	56	53
*A2*a	*E2*	69	95	55	46	55
*A2*a	*E2*a	68	95	55	46	55
*A2*a	*E2*b	67	89	55	44	55

*A2*a	*01*	66	87	43	49	55
B8	*A2*	67	95	70	42	55
B8	*A2*a	69	96	63	42	50
B8	*B8*a	96	100	99	99	86
B8	*C4*	94	100	80	59	84
B8	*D1*y	89	87	86	57	63
B8	*D1*g	89	87	86	57	65
B8	*D1*b	89	85	86	57	65
B8	*D1*d	82	89	86	57	74
B8	*D1*e	90	87	87	57	68
B8	*D1*f	72	93	87	57	70
B8	*E2*	69	95	44	73	70
B8	*E2*a	68	95	45	73	70
B8	*E2*b	65	89	44	69	72
B8	*01*	66	87	34	42	72
*B8*a	*A2*	68	95	69	42	58
*B8*a	*A2*a	70	96	62	43	55
*B8*a	*B8*	96	100	99	99	86
*B8*a	*C4*	96	100	79	59	92
*B8*a	*D1*y	89	87	85	57	75
*B8*a	*D1*g	89	87	85	57	77
*B8*a	*D1*b	89	85	85	57	77
*B8*a	*D1*d	83	89	85	57	85
*B8*a	*D1*e	90	87	86	57	80
*B8*a	*D1*f	74	93	86	57	81
*B8*a	*E2*	71	95	44	73	83
*B8*a	*E2*a	70	89	45	73	83
*B8*a	*E2*b	67	95	44	69	84
*B8*a	*01*	67	87	34	42	83
*D1*y	*A2*	72	93	70	55	51
*D1*y	*A2*a	72	87	64	56	49

*D1*y	*B8*	89	87	86	57	63
*D1*y	*B8*a	89	87	85	57	75
*D1*y	*C4*	91	87	79	76	70
*D1*y	*D1*g	99	100	99	99	90
*D1*y	*D1*b	99	98	100	100	90
*D1*y	*D1*d	88	98	99	98	87
*D1*y	*D1*e	99	100	98	99	94
*D1*y	*D1*f	75	95	98	99	92
*D1*y	*E2*	70	93	45	62	73
*D1*y	*E2*a	70	98	46	62	73
*D1*y	*E2*b	70	93	45	59	74
*D1*y	*01*	71	96	35	67	74
*D1*g	*A2*	72	93	70	55	52
*D1*g	*A2*a	72	87	64	56	50
*D1*g	*B8*	89	87	86	57	65
*D1*g	*B8*a	89	87	85	57	77
*D1*g	*C4*	91	87	78	76	72
*D1*g	*D1*y	99	100	99	99	90
*D1*g	*D1*b	99	98	99	100	100
*D1*g	*D1*d	88	98	99	98	89
*D1*g	*D1*e	99	100	97	99	95
*D1*g	*D1*f	75	95	97	98	94
*D1*g	*E2*	70	93	45	62	75
*D1*g	*E2*a	70	98	45	62	75
*D1*g	*E2*b	70	93	45	59	74
*D1*g	*01*	71	96	35	66	73
*D1*b	*A2*	72	91	70	55	52
*D1*b	*A2*a	72	85	64	56	50
*D1*b	*B8*	89	85	86	57	65
*D1*b	*B8*a	89	85	85	57	77
*D1*b	*C4*	91	85	78	76	72

*D1*b	*D1*y	99	98	100	100	90
*D1*b	*D1*g	99	98	99	100	100
*D1*b	*D1*d	88	96	99	98	89
*D1*b	*D1*e	99	98	97	99	95
*D1*b	*D1*f	75	93	98	98	94
*D1*b	*E2*	71	91	45	62	75
*D1*b	*E2*a	70	96	46	62	75
*D1*b	*E2*b	70	91	45	59	74
*D1*b	*01*	70	94	35	67	73
*D1*d	*A2*	70	95	70	55	58
*D1*d	*A2*a	67	89	64	56	56
*D1*d	*B8*	82	89	86	57	74
*D1*d	*B8*a	83	89	85	57	85
*D1*d	*C4*	84	89	78	76	80
*D1*d	*D1*y	88	98	99	98	87
*D1*d	*D1*g	88	98	99	98	89
*D1*d	*D1*b	88	96	99	98	89
*D1*d	*D1*e	89	98	97	98	93
*D1*d	*D1*f	78	93	97	99	95
*D1*d	*E2*	68	95	45	62	83
*D1*d	*E2*a	68	100	46	62	83
*D1*d	*E2*b	68	95	45	59	82
*D1*d	*01*	69	98	35	67	81
*D1*e	*A2*	72	93	70	55	54
*D1*e	*A2*a	72	87	64	56	52
*D1*e	*B8*	90	87	87	57	68
*D1*e	*B8*a	90	87	86	57	80
*D1*e	*C4*	91	87	79	76	75
*D1*e	*D1*y	99	100	98	99	94
*D1*e	*D1*g	99	100	97	99	95
*D1*e	*D1*b	99	98	97	99	95

*D1*e	*D1*d	89	98	97	98	93
*D1*e	*D1*f	75	95	99	98	98
*D1*e	*E2*	71	93	45	62	78
*D1*e	*E2*a	71	98	46	62	78
*D1*e	*E2*b	71	93	46	59	77
*D1*e	*01*	71	96	34	67	77
*D1*f	*A2*	83	98	70	55	55
*D1*f	*A2*a	77	93	64	56	53
*D1*f	*B8*	72	93	87	57	70
*D1*f	*B8*a	74	93	86	57	81
*D1*f	*C4*	74	93	78	76	76
*D1*f	*D1*y	75	95	98	99	92
*D1*f	*D1*g	75	95	97	98	94
*D1*f	*D1*b	75	93	98	98	94
*D1*f	*D1*d	78	93	97	99	95
*D1*f	*D1*e	75	95	99	98	98
*D1*f	*E2*	75	98	46	62	79
*D1*f	*E2*a	75	93	46	62	79
*D1*f	*E2*b	76	98	46	59	79
*D1*f	*01*	76	91	35	67	79
E2	*A2*	71	100	54	45	57
E2	*A2*a	69	95	55	46	55
E2	*B8*	69	95	44	73	70
E2	*B8*a	71	95	44	73	83
E2	*C4*	71	95	47	56	77
E2	*D1*y	70	93	45	62	73
E2	*D1*g	70	93	45	62	75
E2	*D1*b	71	91	45	62	75
E2	*D1*d	68	95	45	62	83
E2	*D1*e	71	93	45	62	78
E2	*D1*f	75	98	46	62	79

E2	*E2*a	99	100	99	99	100
E2	*E2*b	66	95	93	94	79
E2	*01*	67	93	77	55	78
*E2*a	*A2*	70	100	55	45	57
*E2*a	*A2*a	68	95	55	46	55
*E2*a	*B8*	68	95	45	73	70
*E2*a	*B8*a	70	95	45	73	83
*E2*a	*C4*	70	95	47	56	77
*E2*a	*D1*y	70	93	46	62	73
*E2*a	*D1*g	70	93	45	62	75
*E2*a	*D1*b	70	91	46	62	75
*E2*a	*D1*d	68	95	46	62	83
*E2*a	*D1*e	71	93	46	62	78
*E2*a	*D1*f	75	98	46	62	79
*E2*a	*E2*	99	100	99	99	100
*E2*a	*E2*b	65	95	94	94	79
*E2*a	*01*	67	93	77	55	78
*E2*b	*A2*	74	95	54	43	58
*E2*b	*A2*a	67	89	55	44	55
*E2*b	*B8*	65	89	44	69	72
*E2*b	*B8*a	67	89	44	69	84
*E2*b	*C4*	67	89	47	55	79
*E2*b	*D1*y	70	98	45	59	74
*E2*b	*D1*g	70	98	45	59	74
*E2*b	*D1*b	70	96	45	59	74
*E2*b	*D1*d	68	100	45	59	82
*E2*b	*D1*e	71	98	46	59	77
*E2*b	*D1*f	76	93	46	59	79
*E2*b	*E2*	66	95	93	94	79
*E2*b	*E2*a	65	95	94	94	79
*E2*b	*01*	97	98	79	56	98

01	*A2*	74	93	42	48	57
01	*A2*a	66	87	43	49	55
01	*B8*	66	87	34	42	72
01	*B8*a	67	87	34	42	83
01	*C4*	67	87	36	60	78
01	*D1*y	71	96	35	67	74
01	*D1*g	71	96	35	66	73
01	*D1*b	70	94	35	67	73
01	*D1*d	69	98	35	67	81
01	*D1*e	71	96	34	67	77
01	*D1*f	76	91	35	67	79
01	*E2*	67	93	77	55	78
01	*E2*a	67	93	77	55	78
01	*E2*b	97	98	79	56	98

*These results were generated by NCBI BLAST.

Table S4 | Percent identity matrix for full-length genes highlights similarities among genes

	A2	***A2*a**	***B8***	***B8*a**	***C4***	***D1*y**	***D1*g**	***D1*b**	***D1*d**	***D1*e**	***D1*f**	***E2***	***E2*a**	***E2*b**	***01***
A2		88	53	56	53	61	60	61	61	61	62	49	50	46	44
*A2*a	88		51	54	50	59	57	58	58	58	58	46	47	45	42
B8	53	51		90	77	77	77	77	77	77	77	79	79	74	68
*B8*a	56	54	90		81	81	81	81	82	82	81	74	74	70	64
C4	53	50	77	81		76	75	76	76	76	76	67	67	65	57
*D1*y	61	59	77	81	76		97	100	98	99	98	69	69	66	61
*D1*g	60	57	77	81	75	97		97	95	96	95	69	69	65	61
*D1*b	61	58	77	81	76	100	97		97	98	98	69	69	66	61
*D1*d	61	58	77	82	76	98	95	97		97	97	69	69	65	61
*D1*e	61	58	77	82	76	99	96	98	97		97	69	70	66	61
*D1*f	62	58	77	81	76	98	95	98	97	97		70	70	66	62
E2	49	46	79	74	67	69	69	69	69	69	70		99	91	72
*E2*a	50	47	79	74	67	69	69	69	69	70	70	99		92	72
*E2*b	46	45	74	70	65	66	65	66	65	66	66	91	92		72
01	44	42	68	64	57	61	61	61	61	61	62	72	72	72	
**Ave***	**57**	**55**	**74**	**75**	**69**	**79**	**78**	**79**	**79**	**79**	**79**	**71**	**71**	**68**	**61**

*The average percent identity score for all analyzed *SpTrf* genes is 72

Table S5 | The raw data from the synonymous / nonsynonymous analysis program (SNAP) indicate that some genes are undergoing positive selection while others are undergoing purifying section

Genes compared[1]		Sd[2]	Sn[3]	S[4]	N[5]	pS[6]	pN[7]	dS[8]	dN[8]	dS/dN[9]	pS/pN	dN/dS[10]	pN/pS	Av*
A2	*A2*a	6	10	300.6667	1112.333	0.0200	0.0090	0.0202	0.0090	**2.2363**	2.2197	**0.445545**	0.4500	**0.44**
*B8*a	*B8*	3	5	223.3333	796.6667	0.0134	0.0063	0.0136	0.0063	**2.1506**	2.1403	**0.463235**	0.4701	**0.46**
C4	*C4*a	0	0	200.3333	708.6667	0.0000	0.0000	0.0000	0.0000	**N/A**	N/A	**N/A**	N/A	**N/A**
*D1*f	*D1*h	0	0	216.0000	744.0000	0.0000	0.0000	0.0000	0.0000	**N/A**	**N/A**	**N/A**	**N/A**	**N/A**
*D1*f/h	*D1*d	3	13	215.5000	747.5000	0.0139	0.0174	0.0141	0.0176	**0.7986**	0.8005	**1.248227**	1.2518	**1.10**
*D1*f/h	*D1*e	3.5	16.5	215.3333	747.6667	0.0163	0.0221	0.0164	0.0224	**0.7336**	0.7365	**1.365854**	1.3558	
*D1*f/h	*D1*y	3	12	214.8333	748.1667	0.014	0.016	0.0141	0.0162	**0.8694**	0.8706	**1.148936**	1.1429	
*D1*f/h	*D1*g	4	15	214.8333	748.167	0.0186	0.2000	0.0189	0.0203	**0.9278**	0.9287	**1.074074**	1.0753	
*D1*f/h	*D1*b	5	12	215.0000	748.0000	0.0233	0.0160	0.0236	0.0162	**1.4568**	1.4496	**0.686441**	0.6867	
*D1*d	*D1*e	5.5	18.5	240.1667	839.8333	0.0229	0.0220	0.0233	0.0224	**1.0402**	1.0396	**0.961373**	0.9607	**0.85**
*D1*d	*D1*y	5	13	239.6667	840.3333	0.0209	0.0155	0.0212	0.0156	**1.3535**	1.3486	**0.735849**	0.7416	
*D1*d	*D1*g	6	17	239.6667	840.3333	0.0250	0.0202	0.0255	0.0205	**1.2416**	1.2375	**0.803922**	0.8080	
*D1*d	*D1*b	7	13	239.8333	840.1667	0.0292	0.0255	0.0298	0.0156	**1.9041**	18863	**0.52349**	0.8733	
*D1*e	*D1*y	2.5	7.5	239.5000	840.5000	0.0104	0.0089	0.0105	0.0090	**1.171**	1.1698	**0.857143**	0.8558	**0.92**
*D1*e	*D1*g	3.5	11.5	239.5000	840.5000	0.0146	0.0237	0.0148	0.0138	**1.0688**	1.0681	**0.932432**	1.6233	
*D1*e	*D1*b	4.5	7.5	239.6667	840.3333	0.0188	0.0089	0.0190	0.0090	**2.1178**	2.1038	**0.473684**	0.4734	
*D1*y	*D1*g	1	6	239.0000	841.0000	0.0042	0.0071	0.0042	0.0072	**0.5853**	0.5865	**1.714286**	1.6905	**0.95**
*D1*y	*D1*b	2	2	239.1667	840.8333	0.0084	0.0024	0.0084	0.0024	**3.5298**	3.5157	**0.285714**	0.2857	
*D1*g	*D1*b	1	4	239.1667	840.8333	0.0042	0.0048	0.0042	0.0048	**0.8786**	0.8789	**1.142857**	1.1429	**1.13**
E2	*E2*a	3	3	196.0000	680.0000	0.0153	0.0044	0.0155	0.0044	**3.495**	3.4694	**0.283871**	0.2879	**0.54**
E2	*E2*b	5	9	191.0000	655.0000	0.0262	0.0137	0.0266	0.0139	**1.9214**	1.9052	**0.522556**	0.5229	
*E2*a	*E2*b	4	11	191.0000	655.0000	0.0209	0.0168	0.0212	0.0170	**1.2506**	1.247	**0.801887**	0.8038	

[1]Genes with shared element patterns were analyzed for synonymous vs. nonsynonymous single nucleotide polymorphisms using SNAP v 2.1.1 (https://www.hiv.lanl.gov/content/sequence/SNAP/SNAP.html)

[2]Sd indicates the number of synonymous substitutions.

[3]Sn indicates the number of nonsynonymous substitutions.

[4]S is the potential synonymous substitutions observed as calculated by the average of the compared sequences.

[5]N is the number of potential nonsynonymous substitutions as calculated by the average of the compared sequences.

[6]pS is Sd/S and indicates the proportion of observed synonymous substitutions.
[7]pN is Sn/N. pS/pN and pN/pS are the ratios of synonymous and nonsynonymous substitutions without corrections.
[8]dS and dN are both Jukes-Cantor corrections for the pS and pN
[9]The dS/dN is the ratio of synonymous to nonsynonymous substitutions.
[10]dN/dS is the ratio of nonsynonymous vs. synonymous substitutions. The dN/dS ratio equal to one suggests neutral evolution. The dN/dS ratio of greater than one suggests positive selection while the dN/dS ratio of less than one suggests purifying selection.
*Note that the *D1*b average, while not on the table, is 0.62.

Table S6 | Pairwise distance scores used to calculate percent mismatch for regions of the genes highlight variation in the 5′FR and 3′FR*

Genes compared		5'FR	Exon 1	Intron	Exon 2	3'FR
A2	*A2*a	0.088	0.058	0.048	0.012	0.052
A2	*B8*	0.218	0.058	0.202	0.110	0.397
A2	*B8*a	0.225	0.058	0.216	0.106	0.378
A2	*C4*	0.228	0.058	0.273	0.091	0.461
A2	*D1*y	0.184	0.02	0.220	0.101	0.411
A2	*D1*g	0.182	0.02	0.220	0.106	0.429
A2	*D1*b	0.181	0.04	0.220	0.101	0.429
A2	*D1*d	0.205	0	0.216	0.102	0.408
A2	*D1*e	0.174	0.02	0.212	0.102	0.421
A2	*D1*f	0.118	0.019	0.212	0.097	0.407
A2	*E2*	0.214	0	0.225	0.100	0.395
A2	*E2*a	0.225	0	0.220	0.094	0.395
A2	*E2*b	0.122	0	0.212	0.106	0.333
A2	*01*	0.122	0.02	0.309	0.105	0.360
*A2*a	*B8*	0.200	0.038	0.245	0.110	0.412
*A2*a	*B8*a	0.206	0.038	0.252	0.105	0.453
*A2*a	*C4*	0.210	0.038	0.282	0.090	0.523
*A2*a	*D1*y	0.185	0.084	0.256	0.098	0.464
*A2*a	*D1*g	0.183	0.084	0.260	0.102	0.480
*A2*a	*D1*b	0.182	0.107	0.256	0.098	0.480
*A2*a	*D1*d	0.254	0.061	0.251	0.101	0.443
*A2*a	*D1*e	0.175	0.084	0.242	0.099	0.470
*A2*a	*D1*f	0.172	0.079	0.243	0.099	0.456
*A2*a	*E2*	0.244	0.058	0.199	0.104	0.450
*A2*a	*E2*a	0.252	0.058	0.194	0.101	0.450

*A2*a	*E2*b	0.207	0.061	0.191	0.110	0.390
*A2*a	*01*	0.213	0.084	0.274	0.098	0.404
B8	*A2*	0.218	0.058	0.202	0.110	0.397
B8	*A2*a	0.200	0.038	0.245	0.110	0.412
B8	*B8*a	0.013	0	0.010	0.008	0.011
B8	*C4*	0.026	0	0.096	0.065	0.026
B8	*D1*f	0.187	0.079	0.104	0.065	0.127
B8	*D1*d	0.135	0.061	0.120	0.062	0.128
B8	*D1*e	0.071	0.084	0.104	0.055	0.137
B8	*D1*y	0.079	0.084	0.113	0.062	0.153
B8	*D1*g	0.079	0.084	0.120	0.068	0.149
B8	*D1*b	0.077	0.107	0.113	0.062	0.149
B8	*E2*	0.202	0.058	0.294	0.061	0.146
B8	*E2*a	0.209	0.061	0.288	0.058	0.146
B8	*E2*b	0.209	0.058	0.291	0.075	0.138
B8	*01*	0.201	0.084	0.348	0.070	0.138
*B8*a	*A2*	0.225	0.058	0.216	0.106	0.378
*B8*a	*A2*a	0.206	0.038	0.252	0.105	0.453
*B8*a	*C4*	0.031	0	0.104	0.062	0.080
*B8*a	*D1*f	0.190	0.079	0.111	0.062	0.120
*B8*a	*D1*d	0.145	0.061	0.127	0.059	0.121
*B8*a	*D1*e	0.089	0.084	0.111	0.052	0.129
*B8*a	*D1*y	0.098	0.084	0.121	0.059	0.142
*B8*a	*D1*g	0.092	0.084	0.127	0.065	0.138
*B8*a	*D1*b	0.095	0.107	0.121	0.059	0.138
*B8*a	*E2*	0.194	0.058	0.296	0.059	0.132
*B8*a	*E2*a	0.201	0.061	0.289	0.054	0.132
*B8*a	*E2*b	0.219	0.058	0.293	0.074	0.126
*B8*a	*01*	0.212	0.084	0.341	0.068	0.130
C4	*A2*	0.228	0.058	0.273	0.091	0.461
C4	*A2*a	0.210	0.038	0.282	0.090	0.523

C4	*B8*	0.026	0	0.096	0.065	0.026
C4	*B8*a	0.031	0	0.104	0.062	0.080
C4	*D1*y	0.077	0.084	0.143	0.070	0.222
C4	*D1*g	0.075	0.084	0.149	0.075	0.216
C4	*D1*b	0.075	0.107	0.146	0.070	0.216
C4	*D1*d	0.132	0.061	0.150	0.075	0.189
C4	*D1*e	0.069	0.084	0.139	0.069	0.202
C4	*D1*f	0.194	0.079	0.140	0.069	0.192
C4	*E2*	0.206	0.058	0.321	0.069	0.200
C4	*E2*a	0.213	0.058	0.314	0.069	0.200
C4	*E2*b	0.228	0.061	0.318	0.079	0.196
C4	*01*	0.220	0.084	0.385	0.076	0.196
*D1*y	*A2*	0.184	0.02	0.220	0.101	0.411
*D1*y	*A2*a	0.185	0.084	0.256	0.098	0.464
*D1*y	*B8*	0.079	0.084	0.113	0.062	0.153
*D1*y	*B8*a	0.098	0.084	0.121	0.059	0.142
*D1*y	*C4*	0.077	0.084	0.143	0.070	0.222
*D1*y	*D1*g	0.003	0	0.005	0.007	0.014
*D1*y	*D1*b	0.008	0.02	0.002	0.004	0.014
*D1*y	*D1*d	0.099	0.02	0.005	0.017	0.021
*D1*y	*D1*e	0.008	0	0.023	0.009	0.003
*D1*y	*D1*f	0.176	0	0.015	0.014	0.014
*D1*y	*E2*	0.201	0.02	0.299	0.055	0.143
*D1*y	*E2*a	0.202	0.02	0.293	0.054	0.143
*D1*y	*E2*b	0.172	0.02	0.290	0.069	0.136
*D1*y	*01*	0.172	0.04	0.364	0.055	0.148
*D1*g	*A2*	0.182	0.02	0.220	0.106	0.429
*D1*g	*A2*a	0.183	0.084	0.260	0.102	0.480
*D1*g	*B8*	0.079	0.084	0.120	0.068	0.149
*D1*g	*B8*a	0.092	0.084	0.127	0.065	0.138
*D1*g	*C4*	0.075	0.084	0.149	0.075	0.216

*D1*g	*D1*y	0.003	0	0.005	0.007	0.014
*D1*g	*D1*b	0.010	0.02	0.007	0.005	0.000
*D1*g	*D1*d	0.096	0.02	0.010	0.022	0.027
*D1*g	*D1*e	0.010	0	0.028	0.014	0.010
*D1*g	*D1*f	0.174	0	0.020	0.019	0.020
*D1*g	*E2*	0.198	0.02	0.299	0.058	0.147
*D1*g	*E2*a	0.199	0.02	0.293	0.057	0.147
*D1*g	*E2*b	0.169	0.02	0.290	0.072	0.155
*D1*g	*01*	0.169	0.04	0.374	0.059	0.167
*D1*b	*A2*	0.181	0.04	0.220	0.101	0.429
*D1*b	*A2*a	0.182	0.107	0.256	0.098	0.480
*D1*b	*B8*	0.077	0.107	0.113	0.062	0.149
*D1*b	*B8*a	0.095	0.107	0.121	0.059	0.138
*D1*b	*C4*	0.075	0.107	0.146	0.070	0.216
*D1*b	*D1*y	0.008	0.02	0.002	0.004	0.014
*D1*b	*D1*g	0.010	0.02	0.007	0.005	0.000
*D1*b	*D1*d	0.096	0.04	0.007	0.019	0.027
*D1*b	*D1*e	0.005	0.02	0.025	0.011	0.010
*D1*b	*D1*f	0.173	0.02	0.018	0.016	0.020
*D1*b	*E2*	0.197	0.04	0.299	0.052	0.147
*D1*b	*E2*a	0.198	0.04	0.293	0.051	0.147
*D1*b	*E2*b	0.176	0.04	0.290	0.066	0.155
*D1*b	*01*	0.176	0.061	0.364	0.053	0.167
*D1*d	*A2*	0.205	0	0.216	0.102	0.408
*D1*d	*A2*a	0.254	0.061	0.251	0.101	0.443
*D1*d	*B8*	0.135	0.061	0.120	0.062	0.128
*D1*d	*B8*a	0.145	0.061	0.127	0.059	0.121
*D1*d	*C4*	0.132	0.061	0.150	0.075	0.189
*D1*d	*D1*y	0.099	0.02	0.005	0.017	0.021
*D1*d	*D1*g	0.096	0.02	0.010	0.022	0.027
*D1*d	*D1*b	0.096	0.04	0.007	0.019	0.027

*D1*d	*D1*e	0.090	0.02	0.028	0.023	0.016
*D1*d	*D1*f	0.156	0.02	0.020	0.015	0.006
*D1*d	*E2*	0.221	0	0.294	0.057	0.126
*D1*d	*E2*a	0.221	0	0.287	0.055	0.126
*D1*d	*E2*b	0.183	0	0.284	0.072	0.139
*D1*d	*01*	0.172	0.02	0.347	0.052	0.150
*D1*e	*A2*	0.174	0.02	0.212	0.102	0.421
*D1*e	*A2*a	0.175	0.084	0.242	0.099	0.470
*D1*e	*B8*	0.071	0.084	0.104	0.055	0.137
*D1*e	*B8*a	0.089	0.084	0.111	0.052	0.129
*D1*e	*C4*	0.069	0.084	0.139	0.069	0.202
*D1*e	*D1*y	0.008	0	0.023	0.009	0.003
*D1*e	*D1*g	0.010	0	0.028	0.014	0.010
*D1*e	*D1*b	0.005	0.02	0.025	0.011	0.010
*D1*e	*D1*d	0.090	0.02	0.028	0.023	0.016
*D1*e	*D1*f	0.166	0	0.012	0.020	0.010
*D1*e	*E2*	0.190	0.02	0.297	0.051	0.133
*D1*e	*E2*a	0.191	0.02	0.291	0.050	0.133
*D1*e	*E2*b	0.169	0.02	0.287	0.072	0.140
*D1*e	*01*	0.169	0.04	0.400	0.057	0.152
*D1*f	*A2*	0.118	0.019	0.212	0.097	0.407
*D1*f	*A2*a	0.172	0.079	0.243	0.099	0.456
*D1*f	*B8*	0.187	0.079	0.104	0.065	0.127
*D1*f	*B8*a	0.190	0.079	0.111	0.062	0.120
*D1*f	*C4*	0.194	0.079	0.140	0.069	0.192
*D1*f	*D1*y	0.176	0	0.015	0.014	0.014
*D1*f	*D1*g	0.174	0	0.020	0.019	0.020
*D1*f	*D1*b	0.173	0.02	0.018	0.016	0.020
*D1*f	*D1*d	0.156	0.02	0.020	0.015	0.006
*D1*f	*D1*e	0.166	0	0.012	0.020	0.010
*D1*f	*E2*	0.193	0.019	0.286	0.051	0.125

*D1*f	*E2*a	0.196	0.019	0.280	0.052	0.125
*D1*f	*E2*b	0.115	0.02	0.277	0.063	0.127
*D1*f	*01*	0.115	0.04	0.355	0.045	0.139
E2	*A2*	0.214	0	0.225	0.100	0.395
E2	*A2*a	0.244	0.058	0.199	0.104	0.450
E2	*B8*	0.202	0.058	0.294	0.061	0.146
E2	*B8*a	0.194	0.058	0.296	0.059	0.132
E2	*C4*	0.206	0.058	0.321	0.069	0.200
E2	*D1*y	0.201	0.02	0.299	0.055	0.143
E2	*D1*g	0.198	0.02	0.299	0.058	0.147
E2	*D1*b	0.197	0.04	0.299	0.052	0.147
E2	*D1*d	0.221	0	0.294	0.057	0.126
E2	*D1*e	0.190	0.02	0.297	0.051	0.133
E2	*D1*f	0.193	0.019	0.286	0.051	0.125
E2	*E2*					
E2	*E2*a	0.013	0	0.005	0.007	0.000
E2	*E2*b	0.218	0	0.048	0.029	0.156
E3	*01*	0.206	0.02	0.092	0.050	0.160
*E2*a	*A2*	0.225	0	0.220	0.094	0.395
*E2*a	*A2*a	0.252	0.058	0.194	0.101	0.450
*E2*a	*B8*	0.209	0.058	0.288	0.058	0.146
*E2*a	*B8*a	0.201	0.058	0.289	0.054	0.132
*E2*a	*C4*	0.213	0.058	0.314	0.069	0.200
*E2*a	*D1*y	0.202	0.02	0.293	0.054	0.143
*E2*a	*D1*g	0.199	0.02	0.293	0.057	0.147
*E2*a	*D1*b	0.198	0.04	0.293	0.051	0.147
*E2*a	*D1*d	0.221	0	0.287	0.055	0.126
*E2*a	*D1*e	0.191	0.02	0.291	0.050	0.133
*E2*a	*D1*f	0.196	0.019	0.280	0.052	0.125
*E2*a	*E2*	0.013	0	0.005	0.007	0.000
*E2*a	*E2*a					

*E2*a	*E2*b	0.231	0	0.042	0.030	0.156
*E2*a	*01*	0.210	0.02	0.089	0.055	0.160
*E2*b	*A2*	0.122	0	0.212	0.106	0.333
*E2*b	*A2*a	0.207	0.061	0.191	0.110	0.390
*E2*b	*B8*	0.209	0.061	0.291	0.075	0.138
*E2*b	*B8*a	0.219	0.061	0.293	0.074	0.126
*E2*b	*C4*	0.228	0.061	0.318	0.079	0.196
*E2*b	*D1*y	0.172	0.02	0.290	0.069	0.136
*E2*b	*D1*g	0.169	0.02	0.290	0.072	0.155
*E2*b	*D1*b	0.176	0.04	0.290	0.066	0.155
*E2*b	*D1*d	0.183	0	0.284	0.072	0.139
*E2*b	*D1*e	0.169	0.02	0.287	0.072	0.140
*E2*b	*D1*f	0.115	0.02	0.277	0.063	0.127
*E2*b	*E2*	0.218	0	0.048	0.029	0.156
*E2*b	*E2*a	0.231	0	0.042	0.030	0.156
*E2*b	*01*	0.028	0.02	0.067	0.019	0.009
01	*A2*	0.122	0.02	0.309	0.105	0.360
01	*A2*a	0.213	0.084	0.274	0.098	0.404
01	*B8*	0.201	0.084	0.348	0.070	0.138
01	*B8*a	0.212	0.084	0.341	0.068	0.130
01	*C4*	0.220	0.084	0.385	0.076	0.196
01	*D1*y	0.172	0.04	0.364	0.055	0.148
01	*D1*g	0.169	0.04	0.374	0.059	0.167
01	*D1*b	0.176	0.061	0.364	0.053	0.167
01	*D1*d	0.172	0.02	0.347	0.052	0.150
01	*D1*e	0.169	0.04	0.400	0.057	0.152
01	*D1*f	0.115	0.04	0.355	0.045	0.139
01	*E2*	0.206	0.02	0.092	0.050	0.160
01	*E2*a	0.210	0.02	0.089	0.055	0.160
01	*E2*b	0.028	0.02	0.067	0.019	0.009

*Pairwise distances were generated using Molecular Evolutionary Genetics Analysis (MEGA7)

Table S7 | Percent mismatch scores show similarities for all five regions among genes of the same element pattern*

Genes compared		5'FR	Exon 1	Intron	Exon 2	3'FR
A2	*A2a*	9	9	5	2	4
A2	*B8*	30	9	18	16	57
A2	*B8a*	31	9	19	15	54
A2	*C4*	30	3	22	14	53
A2	*C4*a	30	3	22	14	52
A2	*D1*y	25	3	18	14	53
A2	*D1*g	25	3	17	15	51
A2	*D1*b	25	6	17	14	52
A2	*D1*d	29	0	17	14	52
A2	*D1*e	24	3	17	14	51
A2	*D1*f	18	9	17	14	48
A2	*D1*h	18	9	17	14	47
A2	*E2*	24	0	16	16	58
A2	*E2*a	25	0	15	15	55
A2	*E2*b	18	0	15	15	48
A2	*01*	18	3	16	14	50
*A2*a	*B8*	32	6	21	16	58
*A2*a	*B8a*	33	6	21	15	55
*A2*a	*C4*	31	12	21	14	54
*A2*a	*C4*a	30	12	21	14	53
*A2*a	*D1*y	28	12	19	14	53
*A2*a	*D1*g	28	12	20	15	52
*A2*a	*D1*b	28	16	19	14	53
*A2*a	*D1*d	40	9	19	14	50
*A2*a	*D1*e	27	12	19	14	52
*A2*a	*D1*f	25	6	19	14	49
*A2*a	*D1*h	25	6	19	14	47

*A2*a	*E2*	29	9	14	16	55
*A2*a	*E2*a	30	9	14	16	54
*A2*a	*E2*b	26	9	14	15	49
*A2*a	*01*	27	12	13	14	50
B8	*A2*	30	9	18	16	57
B8	*A2*a	32	6	21	16	58
B8	*B8*	2	0	1	1	1
B8	*C4*	4	12	8	9	4
B8	*C4*a	4	12	8	9	3
B8	*D1*h	26	0	8	9	18
B8	*D1*f	26	0	8	9	18
B8	*D1*d	19	9	9	9	19
B8	*D1*e	10	12	9	8	19
B8	*D1*y	11	12	9	9	20
B8	*D1*g	11	12	9	10	19
B8	*D1*b	11	16	9	9	19
B8	*E2*	24	9	22	8	20
B8	*E2*a	25	9	21	8	20
B8	*E2*b	29	9	22	9	18
B8	*01*	27	12	22	7	18
*B8*a	*A2*	31	9	19	15	54
*B8*a	*A2*a	33	6	21	15	55
*B8*a	*C4*	4	12	9	9	2
*B8*a	*C4*a	5	12	9	9	2
*B8*a	*D1*h	26	0	9	9	16
*B8*a	*D1*f	26	0	9	9	16
*B8*a	*D1*d	20	9	10	9	17
*B8*a	*D1*e	12	12	9	8	17
*B8*a	*D1*y	13	12	9	9	18
*B8*a	*D1*g	13	12	10	9	17
*B8*a	*D1*b	14	16	9	9	17
*B8*a	*E2*	24	9	21	8	19

*B8*a	*E2*a	25	9	21	8	19
*B8*a	*E2*b	30	9	21	9	16
*B8*a	*01*	28	12	21	7	16
C4	*A2*	30	3	22	14	53
C4	*A2*a	31	12	21	14	54
C4	*B8*	4	12	8	9	4
C4	*B8*a	4	12	9	9	2
C4	*C4*a	0	0	0	0	0
C4	*D1*h	26	12	11	10	16
C4	*D1*y	11	0	11	10	19
C4	*D1*g	11	0	11	11	17
C4	*D1*b	11	3	11	10	18
C4	*D1*d	19	3	12	11	17
C4	*D1*e	10	0	11	10	18
C4	*D1*f	26	12	11	10	16
C4	*E2*	24	3	24	10	19
C4	*E2*a	25	3	24	10	19
C4	*E2*b	28	3	24	9	17
C4	*01*	28	6	25	9	17
*C4*a	*A2*	30	3	22	14	52
*C4*a	*A2*a	30	12	21	14	53
*C4*a	*B8*	4	12	8	9	3
*C4*a	*B8*a	5	12	9	9	2
*C4*a	*C4*	0	0	0	0	0
*C4*a	*D1*h	25	12	11	10	16
*C4*a	*D1*y	11	0	11	10	18
*C4*a	*D1*g	11	0	11	11	17
*C4*a	*D1*b	11	3	11	10	17
*C4*a	*D1*d	19	3	12	11	17
*C4*a	*D1*e	10	0	11	10	17
*C4*a	*D1*f	24	12	11	10	16
*C4*a	*E2*	25	3	24	10	19

*C4*a	*E2*a	26	3	24	10	19
*C4*a	*E2*b	28	3	24	9	17
*C4*a	*01*	28	6	25	9	17
*D1*y	*A2*	25	3	18	14	53
*D1*y	*A2*a	28	12	19	14	53
*D1*y	*B8*	11	12	9	9	20
*D1*y	*B8*a	13	12	9	9	18
*D1*y	*C4*	11	0	11	10	19
*D1*y	*C4*a	11	0	11	10	18
*D1*y	*D1*y	24	12	1	2	2
*D1*y	*D1*g	0	0	0	1	1
*D1*y	*D1*b	2	3	0	1	1
*D1*y	*D1*d	14	3	0	2	3
*D1*y	*D1*e	1	0	2	1	0
*D1*y	*D1*f	24	12	1	2	2
*D1*y	*E2*	23	3	21	8	22
*D1*y	*E2*a	23	3	20	8	22
*D1*y	*E2*b	24	3	20	8	18
*D1*y	*01*	23	6	21	6	18
*D1*g	*A2*	25	3	17	15	51
*D1*g	*A2*a	28	12	20	15	52
*D1*g	*B8*	11	12	9	10	19
*D1*g	*B8*a	13	12	10	9	17
*D1*g	*C4*	11	0	11	11	17
*D1*g	*C4*a	11	0	11	11	17
*D1*g	*D1*h	23	12	1	3	2
*D1*g	*D1*y	0	0	0	1	1
*D1*g	*D1*b	2	3	0	1	0
*D1*g	*D1*d	13	3	1	3	2
*D1*g	*D1*e	1	0	2	2	0
*D1*g	*D1*f	23	12	1	3	1
*D1*g	*E2*	23	3	21	9	21

*D1*g	*E2*a	23	3	20	8	21
*D1*g	*E2*b	23	3	20	8	18
*D1*g	*01*	23	6	21	6	19
*D1*b	*A2*	25	6	17	14	52
*D1*b	*A2*a	28	16	19	14	53
*D1*b	*B8*	11	16	9	9	19
*D1*b	*B8*a	14	16	9	9	17
*D1*b	*C4*	11	3	11	10	18
*D1*b	*C4*a	11	3	11	10	17
*D1*b	*D1*h	23	16	1	2	2
*D1*b	*D1*y	2	3	0	1	1
*D1*b	*D1*g	0	0	0	0	0
*D1*b	*D1*d	14	6	1	3	2
*D1*b	*D1*e	1	3	2	2	0
*D1*b	*D1*f	23	16	1	2	1
*D1*b	*E2*	23	6	21	8	21
*D1*b	*E2*a	24	6	20	7	21
*D1*b	*E2*b	25	6	20	7	19
*D1*b	*01*	25	9	21	6	19
*D1*e	*A2*	24	3	17	14	51
*D1*e	*A2*a	27	12	19	14	52
*D1*e	*B8*	10	12	9	8	19
*D1*e	*B8*a	12	12	9	8	17
*D1*e	*C4*	10	0	11	10	18
*D1*e	*C4*a	10	0	11	10	17
*D1*e	*D1*h	22	12	1	3	1
*D1*e	*D1*y	1	0	2	1	0
*D1*e	*D1*b	1	3	2	2	0
*D1*e	*D1*d	14	6	1	3	2
*D1*e	*D1*g	1	0	2	2	0
*D1*e	*D1*f	22	12	1	3	1
*D1*e	*E2*	22	3	20	7	20

*D1*e	*E2*a	23	3	19	8	18
*D1*e	*E2*b	23	3	19	8	18
*D1*e	*01*	23	6	20	6	18
*D1*d	*A2*	29	0	17	14	52
*D1*d	*A2*a	28	12	20	15	52
*D1*d	*B8*	19	9	9	9	19
*D1*d	*B8*a	20	9	10	9	17
*D1*d	*C4*	19	3	12	11	17
*D1*d	*C4*a	19	3	12	11	17
*D1*d	*D1*h	24	9	1	2	1
*D1*d	*D1*y	14	3	0	2	3
*D1*d	*D1*b	14	6	1	3	2
*D1*d	*D1*g	13	3	1	3	2
*D1*d	*D1*e	13	3	2	3	2
*D1*d	*D1*f	24	9	1	2	1
*D1*d	*E2*	27	0	20	8	20
*D1*d	*E2*a	27	0	20	8	20
*D1*d	*E2*b	27	0	19	8	17
*D1*d	*01*	26	3	20	5	18
*D1*f	*A2*	18	9	17	14	48
*D1*f	*A2*a	25	6	19	14	49
*D1*f	*B8*	26	0	8	9	18
*D1*f	*B8*a	26	0	9	9	16
*D1*f	*C4*	26	12	11	10	16
*D1*f	*C4*a	24	12	11	10	16
*D1*f	*D1*h	0	0	0	0	0
*D1*f	*D1*y	24	12	1	2	2
*D1*f	*D1*b	23	16	1	2	1
*D1*f	*D1*d	24	9	1	2	1
*D1*f	*D1*e	22	12	1	3	1
*D1*f	*D1*g	23	12	1	3	1
*D1*f	*E2*	22	9	19	7	19

*D1*f	*E2*a	22	9	19	8	19
*D1*f	*E2*b	14	9	18	7	16
*D1*f	*01*	14	12	19	4	17
*D1*h	*A2*	18	9	17	14	47
*D1*h	*A2*a	25	6	19	14	47
*D1*h	*B8*	26	0	8	9	18
*D1*h	*B8*a	26	0	9	9	16
*D1*h	*C4*	26	12	11	10	16
*D1*h	*C4*a	25	12	11	10	16
*D1*h	*D1*g	23	12	1	3	2
*D1*h	*D1*y	24	12	1	2	2
*D1*h	*D1*b	23	16	1	2	2
*D1*h	*D1*d	24	9	1	2	1
*D1*h	*D1*e	22	12	1	3	1
*D1*h	*D1*f	0	0	0	0	0
*D1*h	*E2*	23	9	19	7	19
*D1*h	*E2*a	23	9	19	8	19
*D1*h	*E2*b	13	9	18	7	16
*D1*h	*01*	14	12	19	4	17
E2	*A2*	24	0	16	16	58
E2	*A2*a	29	9	14	16	55
E2	*B8*	24	9	22	8	20
E2	*B8*a	24	9	21	8	19
E2	*C4*	24	3	24	10	19
E2	*C4*a	25	3	24	10	19
E2	*D1*h	23	9	19	7	19
E2	*D1*y	23	3	21	8	22
E2	*D1*b	23	6	21	8	21
E2	*D1*d	27	0	20	8	20
E2	*D1*e	22	3	20	7	20
E2	*D1*f	22	9	19	7	19
E2	*D1*g	23	3	21	9	21

E2	*E2*a	2	0	0	1	0
E2	*E2*b	23	0	4	2	21
E2	*01*	19	3	3	5	22
*E2*a	*A2*	25	0	15	15	55
*E2*a	*A2*a	30	9	14	16	54
*E2*a	*B8*	25	9	21	8	20
*E2*a	*B8*a	25	9	21	8	19
*E2*a	*C4*	25	3	24	10	19
*E2*a	*C4*a	26	3	24	10	19
*E2*a	*D1*h	23	9	19	8	19
*E2*a	*D1*y	23	3	20	8	22
*E2*a	*D1*b	24	6	20	7	21
*E2*a	*D1*d	27	0	20	8	20
*E2*a	*D1*e	23	3	20	7	21
*E2*a	*D1*f	22	9	19	8	19
*E2*a	*D1*g	23	3	20	8	21
*E2*a	*E2*a	2	0	0	1	0
*E2*a	*E2*b	23	0	3	3	22
*E2*a	*01*	20	3	2	5	22
*E2*b	*A2*	18	0	15	15	48
*E2*b	*A2*a	26	9	14	15	49
*E2*b	*B8*	29	9	22	9	18
*E2*b	*B8*a	30	9	21	9	16
*E2*b	*C4*	28	3	24	9	17
*E2*b	*C4*a	28	3	24	9	17
*E2*b	*D1*h	13	9	18	7	16
*E2*b	*D1*y	24	3	20	8	18
*E2*b	*D1*b	25	6	20	7	19
*E2*b	*D1*d	27	0	19	8	17
*E2*b	*D1*e	23	3	19	8	18
*E2*b	*D1*f	14	9	18	7	16
*E2*b	*D1*g	23	3	20	8	18

*E2*b	*E2*	23	0	4	2	21
*E2*b	*E2*a	23	0	3	3	22
*E2*b	*01*	5	3	1	3	1
01	*A2*	18	3	16	14	50
01	*A2*a	27	12	13	14	50
01	*B8*	27	12	22	7	18
01	*B8*a	28	12	21	7	16
01	*C4*	28	6	25	9	17
01	*C4*a	28	6	25	9	17
01	*D1*h	14	12	19	4	17
01	*D1*y	23	6	21	6	18
01	*D1*b	25	9	21	6	19
01	*D1*d	26	3	20	5	18
01	*D1*e	23	6	20	6	18
01	*D1*f	14	12	19	4	17
01	*D1*g	23	6	21	6	19
01	*E2*	19	3	3	5	22
01	*E2*a	20	3	2	5	22
01	*E2*b	5	3	1	3	1

*****The percent mismatch results between all gene pairs for the five regions were calculated based on the data in Table S5. These data are shown in graphical format in Figure 5 in the main paper.
